# Decomposing Vehicle Production and Consumption Impacts in Japan under Mobility and Production Network Interventions

**DOI:** 10.1021/acs.est.6c06919

**Published:** 2026-09-13

**Authors:** Jacob Fry, Ziyan He, Hiroyuki Ishikawa, Takamichi Iwata, Minami Kito, Kenichi Nakajima, Keisuke Nansai

**Affiliations:** † 13585 National Institute for Environmental Studies, Tsukuba, Ibaraki 305-8506, Japan; ‡ Toyota Central R&D Laboratories. Inc., Nagakute, Aichi 480-1192, Japan; § Fukuoka Institute of Technology, Wajiro-Higashi, Higashi-ku, Fukuoka 811-0295, Japan

**Keywords:** vehicles, emissions, materials, MRIO, demand-side, mobility

## Abstract

Passenger vehicles contribute approximately 8% to global energy-related emissions and drive other environmental impacts through the extraction of raw materials. In this study, a hybrid LCA approach is used to investigate emissions and material life-cycle impacts arising from vehicle production and consumption in Japan. By embedding a physical vehicle production model within a global MRIO, we find that 53% of emissions occur in Japan, while almost all material extraction impacts occur internationally. Vehicle size, PHEV utility factor, and battery capacity all strongly influence life-cycle impacts. Demand-side scenarios of increased occupancy, downsizing, and lifetime extension have the collective potential to reduce emissions by approximately 33% at the cohort level, while providing comparable mobility services. Lifetime extension requires additional material inputs with associated impacts; however, these impacts are repaid over time, resulting in an overall average emissions intensity (gCO_2_e/km) reduction of 7%. Vehicle electrification alone is unlikely to be sufficient to meet climate targets; therefore, demand-side measures should be implemented concurrently to decarbonize passenger transport while maintaining access to mobility services. While some opportunity exists to substitute upstream suppliers for lower-impact providers, the existence of low-pollution production havens is limited, further underscoring the importance of demand-side measures.

## Graphical abstract

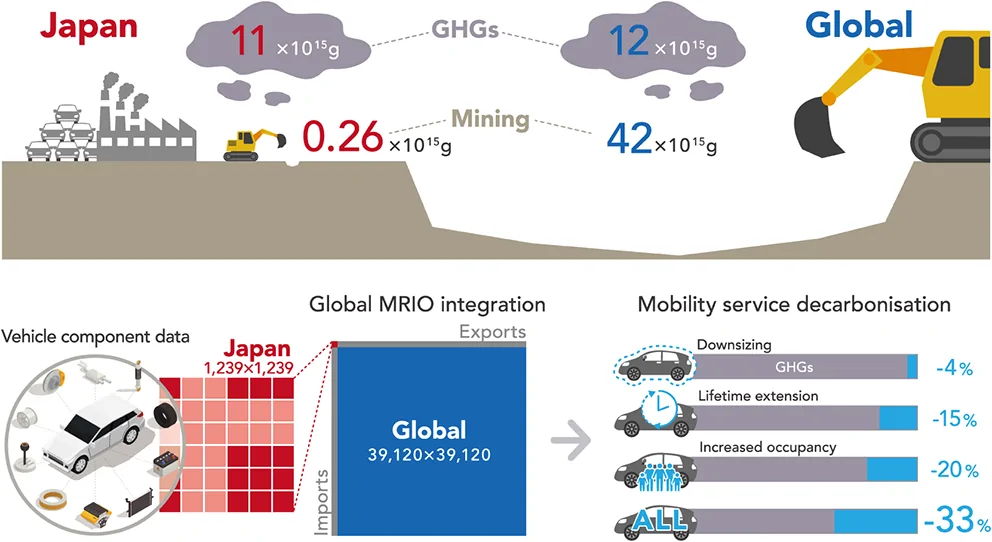

## 1 Introduction

Emissions from passenger vehicles contributed approximately 8% to global energy-related emissions in 2022.^1^ The transition away from internal combustion vehicles (ICVs) is a key decarbonization strategy for the transport sector, with some nations planning and implementing ICV sale bans.^2^ Alternate vehicle technologies include Hybrid Electric Vehicles (HEVs), Plug-in Hybrid Electric Vehicles (PHEVs) and battery electric vehicles (BEVs) and hydrogen fuel-cell vehicles (FCVs). Comparing the environmental impacts of these vehicle technologies can be complicated for several reasons. First, vehicles are composed of many thousands of components, making tracing total environmental impacts difficult and requiring analyses with global scope.^3^ Second, vehicle production and use affects can be measured using multiple indicators, such as GHG emissions, materials, biodiversity, complicating comparisons between vehicle technologies. Furthermore, comparisons between vehicle impact studies can be difficult because of differing assumptions.^4^

The Industrial Ecology literature of Lifecycle Assessment (LCA) studies of passenger vehicles is extensive, with many studies calculating the environmental impacts of producing and using vehicles, and end-of-life. Impacts from manufacturing HEVs and BEVs are generally higher than ICVs, for example^5^ found that BEV production emissions were 1.5–2 times higher than ICVs. Impacts associated with battery production are the main contributor to the higher impacts associated with BEVs.^6^ Battery production is also fraught with land-use conflicts in the extraction of minerals, energy requirements of production, and availability of raw materials.^7^ Biodiversity impacts resulting from mining are also high for EVs and are associated with the extraction of lithium, nickel and cobalt.^8^

Impacts from vehicles are distributed over time, occurring during the production, use, maintenance and end-of-life phases^9^ found that use-phase emissions for ICVs are approximately 70–90% of vehicle lifetime emissions, while for EVs use-phase emissions are on the order of 40–65% of lifecycle impacts. Crucial to the emissions reduction potential of electric vehicle use-phase is the decarbonization of the electricity grid,^10^ though forecasts of future electricity grid emissions intensity are uncertain. Emissions from PHEVs are sensitive to the relative utilization of the internal combustion engine and electric motor, which is dictated by real-world driving behavior.^11^

Reducing the number of vehicles in the fleet and extending vehicle lifetimes can reduce total vehicle impacts.^12^ Whether lifetime extension is beneficial depends on the additional material requirements (i.e., repair and maintenance) and forfeited efficiency gains from upgrading to newer vehicles.^13^ Reducing the vehicle fleet size can increase the lifetime distance traveled by the remaining vehicles, therefore to model such scenarios it is necessary to widen the analysis to the vehicle fleet.^14^ Increasing the vehicle lifetime can prevent or delay the production of new vehicles, therefore vehicle fleet dynamics over time must also be considered. Linking demand-side “mobility-service” interventions at the fleet level, such as vehicle downsizing and increasing vehicle occupancy, with global emissions and material use can also provide important insights.^15^

Materials production contributes significantly to vehicle lifecycle impacts. Demand-side measures, such as reducing the number of vehicles in the fleet and extending vehicle lifetimes, can reduce these material requirements.^12^ Whether lifetime extension is beneficial depends on the additional material requirements (i.e., repair and maintenance) and forfeited efficiency gains from upgrading to newer vehicles.^13^ Reducing the vehicle fleet size can increase the lifetime distance traveled by the remaining vehicles, therefore it is necessary to model the vehicle fleet and overall mobility task.^14^ Increasing the vehicle lifetime can prevent or delay the production of new vehicles, reducing new production while requiring more maintenance. Other circular economy and material efficiency strategies, such as lightweighting, remanufacturing and recycling, can also reduce vehicle material use.^16^ The transition to electric vehicles will particularly require strong circular economy policies to offset battery material requirements.^17^ Meeting climate targets will likely require reducing overall material use and demand-side measures are required to achieve this.^18^

Process-based LCA is a bottom-up technique for assessing the environmental impacts resulting from product lifecycles i.e., extraction, production, use-phase and waste disposal.^19^ PLCA is used to study very specific processes and products and guidelines exist to that define the assessment scope and evaluate significance of the results.^20–22^ However, PLCA can suffer from system boundary incompleteness when higher-order production stages are excluded from the assessment.^23,24^ Input-output analysis (IOA) is widely employed to calculate environmental impacts occurring along the supply chains that deliver goods and services to final consumption.^25^ IOA attributes all impacts produced by the economy to the final consumption of good and services,^26^ however the resolution of MRIO models is usually limited to aggregate sectors and hence it is difficult to calculate the impact of very specific products or services.^27^

PLCA and IO methods can be combined in hybrid assessments that link the microstructure of individual extraction and manufacturing processes with activities and processes in the larger macro-scale economy. The aim of this hybridization is to reduce the error inherent in both methods; PLCA allows specific products to be studied, while IOA maximizes system boundary completeness.^28^ Hybridization techniques include tiered-hybrid analysis,^27^ path-exchange,^29^ matrix augmentation,^30^ and the integrated approach.^31^ While the intent is to reduce error, hybrid methods can introduce new issues, such as double counting at the interface between the process and IO models when process inputs are not removed from the IO system.^32^ In the matrix augmentation approach, disaggregated sectors may have different output structures than the original sectors, which should be accounted for.^33^ In addition, the price data used to link the physical and monetary components of the model can also introduce uncertainty.^34^ For example, prices for the same commodity may vary across industries, and average prices for commodity categories may mask heterogeneous collections of products and prices.

Global MRIOs are constructed by combining national statistics and global trade data to produce a harmonized account.^35^ However, this harmonization can result in a representation of a domestic economy less detailed than official IO tables for that nation. One solution is to “nest” the national IO table (IOT) within the global model, replacing the lower-detail segments.^36^ Such nested models can link subnational with global regions^37,38^ and also integrate firms or enterprises into larger models, such that the set of IO accounts remains consistent.^39^

This study follows a hybrid-LCA approach to assess environmental impacts associated with passenger vehicle production and consumption in Japan. First, physical component data for Japanese vehicles are compiled by vehicle technology and size (sections 2.1–2.2). These physical data are embedded first within a high-resolution Japanese national IO model, and then within a global MRIO (Section 2.3). Vehicle components are fully interlinked with the Japanese and global economies so that all inputs to production, including services and infrastructure, are captured (Sections 2.4). Emissions and material extraction impacts are assessed over the vehicle lifecycle (Section 2.6) for a range of parameter values, including: future grid emissions intensity (Section 2.7.1), biofuels (Section 2.7.2) and PHEV electric motor utilization (Section 2.7.3). The emissions implications of vehicle lifetime extension are also examined (Section 2.7.4), including the material requirements for repair and maintenance. Widening the analysis to the vehicle cohort, the decarbonization potential of demand-side mobility strategies (vehicle downsizing and increased occupancy) is modeled (Section 2.7.5). Japan is a hub for vehicle manufacturing and associated industries, though many vehicle production impacts occur internationally. The ability of Japanese vehicle producers to reduce upstream impacts is tested by searching upstream paths and testing whether substitution for lower impact paths is possible (Section 2.8).

## 2 Method

### 2.1 Vehicle Material Composition

This study considers internal combustion (ICV), hybrid (HEV), plug-in hybrid (PHEV), and battery electric (BEV) vehicle powertrain technologies. The GREET database^40^ was used to define the material composition by powertrain technology, with vehicle weights scaled for the Japanese context (see SI-1.1–1.2). GREET provides the material compositions of each component, including the vehicle body, powertrain system, transmission system, chassis, traction motor, generator, and electronic controller. It was assumed HEV, PHEV and BEV vehicles use NMC (lithium nickel manganese cobalt oxide) batteries. Battery composition was sourced from^41^ and assumed to scale linearly with vehicle weight. Vehicle weight by component is shown in Figure 1 (panel (iii). Vehicle weight is dominated by the body and chassis components (54%–78% of total vehicle weight), which comprise mostly of steel and aluminum. For PHEV and BEVs, the battery cell and case also contribute significantly to total weight (14%–22%). The PHEV powertrain is proportionately larger since these vehicles require both electric and internal combustion powertrains. The BEV powertrain and transmission is comparatively lighter due to simpler design and fewer parts.

**Figure 1:**
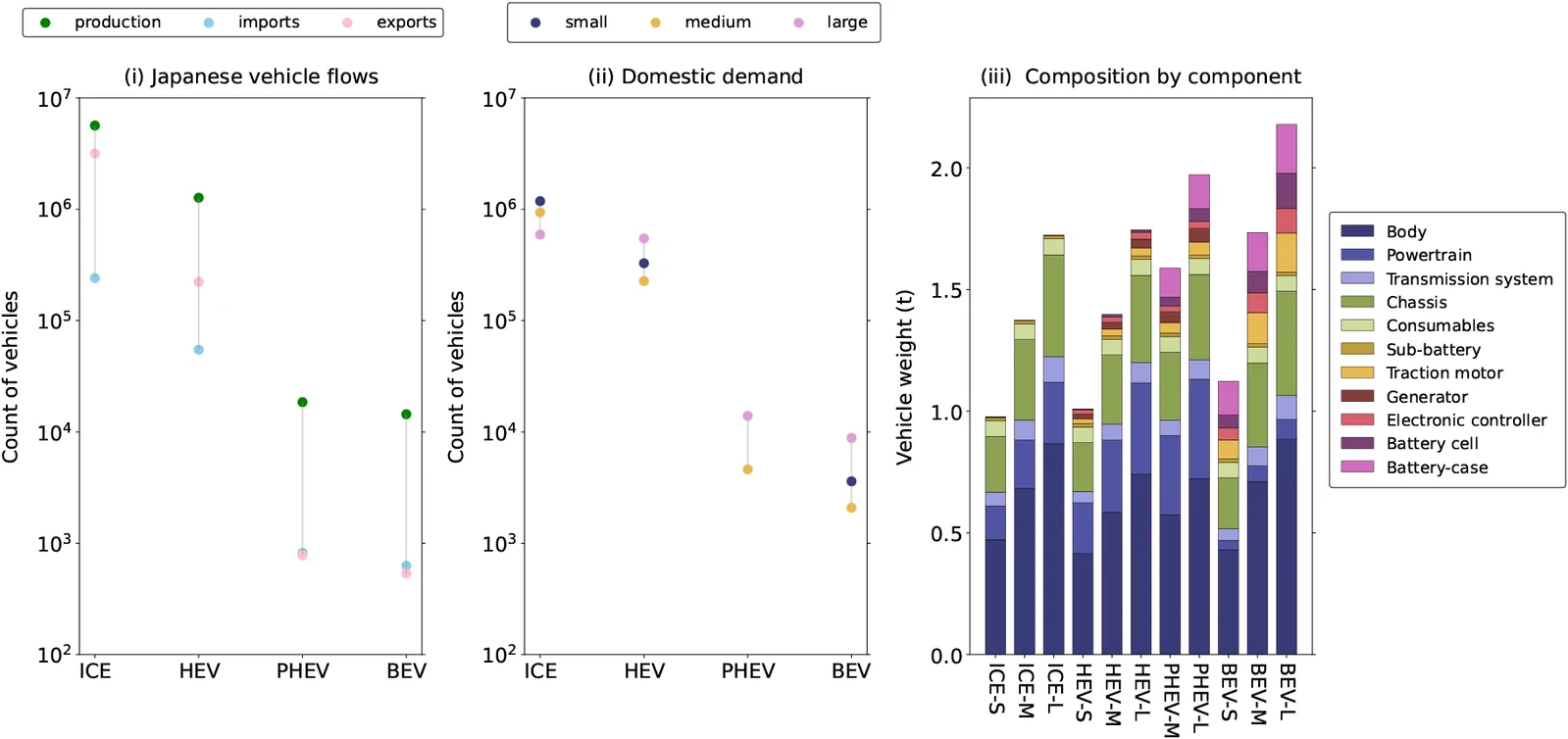
Panel (i) Japanese vehicle production and trade, (ii) domestic consumption of vehicles by size and powertrain, (iii) vehicle weight by component.

### 2.2 Vehicle Cohort

Japanese vehicle production and consumption statistics were estimated from^42–44^ and shown in Figure 1 panels (i) and (ii). In 2020, Japan produced 6.9 million vehicles, with consumption split between ICV (70%), HEV (27%), PHEV (0.3%) and BEV (1%). Exports are about 49% of total vehicle production. In 2020, domestic vehicle demand was split between large (35%), medium (29%), and small (35%) vehicles, where sizes are equivalent to large: D-segment SUVs (and includes large sedans and people movers), medium: C-segment sedans, small: A-segment minicar (or *kei car*). Small sized PHEVs are not considered as there are currently no vehicles sold in this category (see SI-1.3).

### 2.3 Constructing a Japan-Global Nested MRIO

The GLORIA MRIO contains 164 regions comprised of 120 sectors with the vehicle sector represented by *Motor vehicles, trailers and semi trailers*.^45^ To increase the resolution of this MRIO, the official 390-sector Japan IOT^46^ is nested inside the GLORIA MRIO, using the year 2020 for both models. The Japan IOT is represented by **T̃** ^*rr*^ in eq 1, with import and export vectors **M** and **X**. In the nested MRIO, the Japan IOT replaces the Japan blocks in the global model (eq 2), where region *r* refers to Japan and *s* is the set of 163 global regions (excluding Japan). The final demand matrix is also nested (eq 3) where for example **y** ^*sr*^ stores consumption of goods in region *r* produced in region *s*. A detailed heatmap of the nested MRIO is shown in.
1

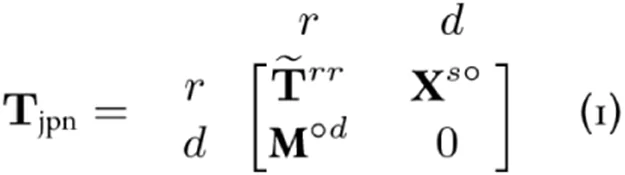



2

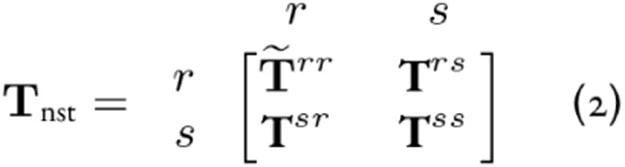



3

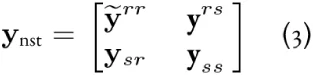




To improve the alignment between the nonferrous metal categories in the Japan IOT and GLORIA, the Japanese nonferrous metals category is disaggregated using production data^47^ and the Japan WIO-MFA data set^48,49^ see SI-2.3 for more detail.

### 2.4 Component Model Augmentation

Vehicle production is modeled in detail by embedding a process model as a subsystem of the larger economy.^50^ This process model contains all materials and components required for automobile production in physical units (tonnes), as well as direct impacts such as emissions. Following the matrix augmentation approach, the process model is represented by the matrix **T** ^*pp*^ in eq 4. The matrices **T** ^*rp*^ and **T** ^*sp*^ describe upstream inputs to the process model from the Japanese and global economies, with imports from the domestic economy defined in **T** ^*rp*^ and imports from global regions defined in **T** ^*sp*^. These matrices lie at the interface of the two models, linking the process model with the IO model and wider economy and are analogous to the upstream and downstream cutoff matrices in tiered-hybrid analysis.^51^ Japanese vehicle production supplies finished vehicles to Japanese domestic final demand only, therefore the matrices **T** ^*pr*^ and **T** ^*ps*^ are zero.
4

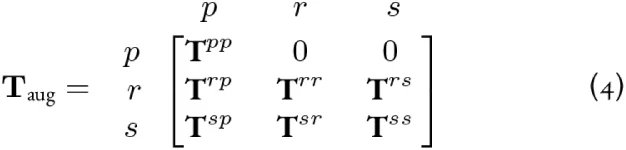




The process model **T** ^*pp*^ is expanded in eq 5 to detail vehicle technologies *k* (*ICV, HEV, BEV, PHEV*), vehicle sizes *z* (*small, medium, large*), components *h* (e.g., *body, powertrain, transmission*, etc.) and materials *m* (e.g., *steel*, *glass*, *polyurethane*, etc.). While only one vehicle technology is shown (e.g., *k*_1_ = *ICV*, *k*_2_ = *HEV*, etc.), this is easily extended to *n* technology types. The inclusion of physical units results in a mixed-unit input-output table containing both physical and monetary rows.^52,53^


5

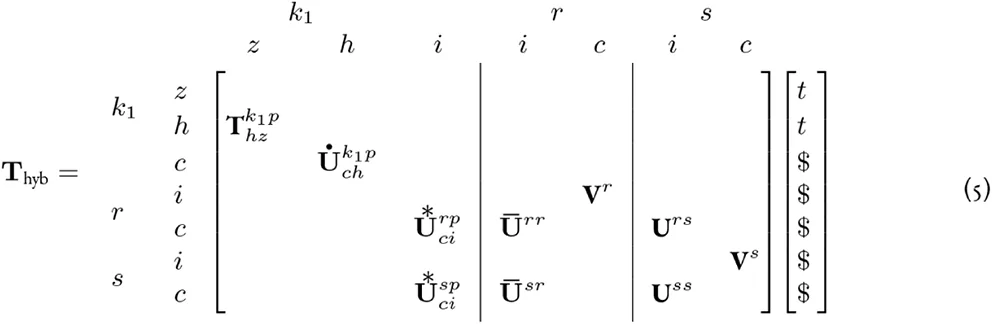




In eq 5, **U̅** is the posthybridization Japan Use matrix and also contains the remainder of the vehicle industry, for example production of vehicles for export markets. $\overset{*}{U}$ contains exogenous monetary inputs to the vehicle production industry, from both Japan ${\overset{*}{U}}^{rp}$ and the global economy ${\overset{*}{U}}^{sp}$ . The matrix U̇ bridges inputs of commodities from domestic and foreign sources to vehicle components, in monetary units. The matrix **y** ^*kz*^ contains final consumption of vehicles in physical units (full structure shown in SI-2.4).

### 2.5 Reconciling Inputs to Vehicle Production

Physical vehicle production data are then linked to the MRIO monetary inputs. Often physical and monetary data are linked using prices, however this conversion is influenced by the uncertainty and variation in prices. The physical data may also be incomplete, for example the assembly phase of vehicle production includes many nonmaterial inputs, such as electricity, freight, financial and legal services. Nonessential materials are also missing from the vehicle data, such as textiles for vehicle interiors. Ideally, both the materials balance of the component data and the monetary balance of the MRIO should be preserved. To reconcile these issues, the monetary and physical flows are reconciled simultaneously (following^54^ by framing model construction as a constrained optimization problem and solving by the RAS algorithm.^55,56^ First, the vehicle material composition data **G** is used to construct the process model block:

6 $$\underset{m}{\sum }G_{mh}^{kz}=T_{h}^{kz}$$

An initial estimate of relationships between physical vehicle components *h* and monetary inputs *i* is defined by a qualitative prior (QP)^57^ where $0\leq C_{ih}^{QP}\leq 1\forall i,h$ . The QP allows some links (e.g., steel into body parts) while prohibiting other links (e.g., inputs of internal combustion engines to BEVs). Physical materials are related to commodities using a vector of prices **ρ**, derived from the Comtrade database^58^ and aggregated using a trade volume weighted mean. Materials are then linked to monetary inputs by

7 $$\underset{m}{\sum }G_{mh}^{kz}\rho_{m}C_{mi}C_{ih}^{QP}=\underset{i}{\sum }{\mathrm{U̇}}_{ih}^{k}$$

where the normalized map **C**_*mi*_ distributes materials across commodities. Monetary inputs from the MRIO system are preserved across the process model inputs:

8 $$\underset{kh}{\sum }{\mathrm{U̇}}_{ih}^{k}=\underset{r}{\sum }\overset{*}{U}{\;}_{i}^{r}\forall i$$

Inputs to vehicle production are conserved over both regions and commodities, by ensuring all original inputs are allocated either to $\overset{*}{U}$ or **U̅**:

9 $${\mathrm{U̅}}_{i}^{rv}=U_{i}^{rv}-\overset{*}{U}{\;}_{i}^{rv}\forall i,r$$

The proportions of commodity *c* allocated among components *h* are set according to the ratio constraints:

10 $$\underset{k}{\sum }{\mathrm{U̇}}_{hc}^{k}/\underset{kh}{\sum }{\mathrm{U̇}}_{hc}^{k}\approx \underset{kzm}{\sum }G_{mh}^{kz}/\underset{kzmh}{\sum }G_{mh}^{kz}\forall h$$

### 2.6 Environmental Impact Assessment

Environmental impacts resulting from vehicle production can be estimated using the IO footprint calculus *F* = **qLy**.^59^ Here the emissions intensity is **q** = **Q x̂** ^–1^ where **x** is a vector of total output and **Q** is a matrix of environmental impacts, defining impacts by industry and emitting region. The Leontief inverse is given by **L** = (**I** – **A**)^−1^ where **A** is a matrix of input coefficients **A** = **Tx̂** ^–1^ and the hat symbol denotes diagonalization. In this study, the final demand segment **y** ^*pr*^ contains consumer demand for vehicles produced and consumed in Japan. Note that segments: imported vehicles (**y** ^*sr*^), vehicles produced outside the process model (**y** ^*rr*^) and exported vehicles (**y** ^*rs*^) are excluded. The footprint *F*_ρ_ = **qLy** ^*ps*^ then quantifies impacts occurring both domestically in Japan and globally resulting from the consumption of vehicles produced and consumed in Japan. The matrix **Q** can be partitioned by producing region: **Q** = [**Q** ^*p*^ **Q** ^*r*^ **Q** ^*s*^], where **Q** ^*p*^ and **Q** ^*r*^ are constructed from Japanese environmental accounts^60^ and **Q** ^*s*^ originates from GLORIA. Here **Q** contains emissions (t CO_2_e) and materials (tonnes material extraction) rows. While material footprints do not directly measure environmental impacts, such as biodiversity loss and deforestation, the indicator is a useful proxy for pressure on the natural environment.^61^

Fuel-related emissions from the vehicle use-phase can be written $F_{u}^{kz}=\underset{t}{\sum }\left(1/e_{kz}\right)df$ , where *e* is the vehicle fuel efficiency (km/L or km/kWh) as a function of vehicle technology and size, *d* is the distance driven per year (km), and *f* the fuel emissions intensity (kg CO_2_e/L or kg CO_2_e/kWh). For electric vehicles, the electricity grid emissions intensity changes over time so that $F_{u}^{kz}=\underset{t}{\sum }\left(1/e^{kz}\right)df_{t}$ , where *f*_*t*_ is the grid emissions intensity in year *t* (see section 2.7.1 ). The vehicle requires additional materials for maintenance and other consumables over the lifetime, for example engine oil, brake fluid and tires. The impacts from producing these materials is $F_{\gamma }^{kz}=\underset{t}{\sum }\lambda_{mt}^{kz}\mathrm{qL}y_{m}$ , where **λ** is a binary matrix specifying whether material *m* in vehicle type *kz* requires replacement in year *t*. Lifetime impacts are the sum over production and use-phase emissions: *F* = *F*_ρ_ + *F*_*u*_ + *F*_γ_, with assumed vehicle lifetime of 150,000 km over 15 years. Fuel efficiency and emissions intensity by vehicle properties are given in SI-4. SI-3 shows the system boundary.

### 2.7 Future Scenario Sensitivity

The following scenarios were modeled to assess the sensitivity of the results to input parameter values and to represent uncertainty in the estimates of future parameter values:

#### 2.7.1 Grid Emissions Intensity

Three scenarios of future grid emissions intensity are modeled in this study: (i) IEEJ Reference (ref.), (ii) IEEJ Advanced technologies (AT)^62^ and (iii) no new climate policies beyond 2035 (static). For each electricity generation type, lifecycle emissions were estimated using^63^ emissions intensities. These effect of these scenarios on grid emissions intensity is shown in SI-3.2.

#### 2.7.2 Biofuel Blends

Biofuels, made from materials such as corn stover, wheat straw and sugar cane bagasse, have the potential to reduce emissions compared to standard petrol. Three fuel scenarios are modeled: i. “standard” petrol (with Scope 3 emissions intensity of 85 gCO_2_e/MJ), ii. E10 and iii. E20 bioethanol petrol blends. The emissions reductions from E10 and E20 are modeled as 4% and 8% respectively^64,65^ and it is assumed these fuels are available in Japan from 2025.

#### 2.7.3 PHEV Utilization

PHEV use-phase emissions are dependent on the fuel efficiency of both the electric and internal combustion (IC) powertrains, as well as the relative utilization of electric and IC modes. This can be expressed as *F*_*u*_ = *d*[(η (1/*e*_*v*_) *f*_*g*_ + (1 – η) (1/*e*_*i*_) *f*_*p*_)], where η is the electric motor utilization factor, *e*_*v*_ the electric motor intensity (km/kWh), *f*_*g*_ the grid emissions intensity (kg CO_2_e/kWh), *e*_*i*_ the internal combustion intensity (km/L) and *f*_*p*_ the emissions intensity of petrol (kg CO_2_e/L). Utilization η is modeled in the range 0.3–0.9, reflecting a mix of real-world driving conditions.^66,67^

#### 2.7.4 Lifetime Extension

In this scenario, vehicle lifetimes are extended from 15 to 22 years (section 3.2) and 30 years (section 3.3). The driving distance increases proportionally to 220,000 km and 300,000 km, respectively. Extending the vehicle lifetime requires additional materials, for example, replacing cracked windshields, spark plugs, brake pads, bearings and coolant fluids. For PHEVs and BEVs, lifetime extension includes a replacement traction battery. Total impacts become: *F* = *F*_ρ_ + *F*_*u*_ + *F*_γ_ + *F*_ϕ_, where *F*_ϕ_ is the footprint of the additional material requirements.

#### 2.7.5 Mobility Services

The following strategies deliver constant mobility services while reducing material and energy use and are evaluated at the vehicle cohort level. Here we follow one slice of the total passenger vehicle mobility task through time. While older vehicles leave the fleet, and newer vehicles join the fleet, the modeled cohort delivers a constant segment of the total mobility task (see SI-5.5). Total impacts from production and use over vehicle lifetimes in the cohort are *F́* = ∑_*kz*_ **N** ^*kz*^ ◦ (**F**_ρ_ + **F**_*u*_ + **F**_γ_ + **F**_ϕ_), where **N** ^*kz*^ contains counts of vehicles in each class. The mobility services provided by the fleet can be written M = ∑_*kz*_ σ*d* **N** ^*kz*^, where σ is the average vehicle occupancy and *M* has units of passenger-km.^68^ Two mobility strategies are evaluated:

(1) *Downsizing*: Prior to 2000, the fraction of large vehicles (large sedans and SUVs) sold in Japan was ≈18%, by 2024 this fraction increased to ≈34%.^43^ To model a policy of vehicle downsizing, large vehicles are swapped for medium sized vehicles to match historical pre-2000 levels. The total number of vehicles remains the same after swapping and we assume that medium-sized vehicles are capable of the same average occupancy as large vehicles. Note this implies the simplification that large vehicles have similar average occupancy to medium size vehicles.
(2) *Occupancy*: Average vehicle occupancy in Japan is ≈1.5 passengers per vehicle.^69^ Keeping total vehicles and mobility services constant (*M*_1_ = *M*_2_ and $\underset{kz}{\sum }N_{1}^{kz}=\underset{kz}{\sum }N_{2}^{kz}$ ), the change in driving distance resulting from a change in occupancy is *d*_2_ = (σ_1_ *d*_1_)/σ_2_. An increase in vehicle occupancy results in lower vehicle-km traveled, however increasing occupancy also marginally increases the total weight of the vehicle, which in-turn effects fuel economy. To account for this, a regression model was used to approximate the relationship between fuel economy and vehicle weight (including occupants) (see SI-4.1).

Supporting calculations for these mobility strategies are given in SI-5.

### 2.8 Structural Path Analysis and Input Substitution

Path analysis can identify hotspots in vehicle supply chains, such as glass production,^70^ which can assist companies in reducing upstream Scope 3 impacts. Companies may switch to more cleaner suppliers or exert pressure on existing suppliers to become more efficient. Vehicle production impacts can be unraveled into constituent supply chain paths using a power series expansion:^71^

11 $$F=\overset{n}{\underset{i,j=1}{\sum }}q_{i}\left(\delta_{ij}+\overset{n}{\underset{k=1}{\sum }}A_{ik}A_{kj}+\overset{n}{\underset{l=1}{\sum }}\overset{n}{\underset{k=1}{\sum }}A_{il}A_{lk}A_{kj}+...\right)y_{j}$$

where δ_*ij*_ is the Kronecker delta and δ_*ij*_ = 1 if *i* = *j* and zero otherwise (see SI-6 for more detail). To test the ability of Japanese vehicle manufacturers to reduce upstream 3 impacts, the substitution of high-intensity paths with lower impact paths is modeled. Consider the path $S=q_{i}^{\theta }A_{ij}^{\theta r}A_{jk}^{rr}y_{k}^{r}$ where impacts occurs in region θ resulting from consumption in region *r*. We search for a substitute supplier ω of commodity *i*: $S^{\prime }=q_{i}^{\omega }A_{ij}^{\theta r}A_{jk}^{rr}y_{k}^{r}$ such that *S*′ < *S*. The search space is constrained so that substitutions are permitted when (i) the second-last supply chain node is located in Japan, (ii) the exporter has sufficient export capacity and (iii) the export structure of ω is sufficiently similar to θ (see SI-6.2–6.3). While manufacturers can switch individual product supply chains to minimize upstream emissions, however there may not be enough capacity upstream to satisfy all demand for low-emissions production. Hence these examples of cleaner production show the potential of low-impact technology diffusion, rather than actual trade relocation.

## 3 Results

### 3.1 Vehicle Production Impacts

In Figure 2, panels (i) and (ii) show emissions and materials footprints associated with vehicle production by component. In panel (i), emissions footprints range from ICV: 5.1–8.4 t, HEV: 5.6–9.2 t, PHEV: 8.5–10.8 t, BEV: 6.3–13.6 t. Body and Powertrain are together are the largest contributors to the emissions footprints (ICV: 19%, HEV: 14%, PHEV: 9% and BEV: 9%), while the battery dominates BEV impacts (42%). Vehicle Assembly contributes 12–14% to vehicle footprints, where Assembly includes services inputs from services, construction, utilities and other inputs to production, such as machine tools. In panel (ii), the largest material contributions to component footprints are Battery cell (fossil fuels 11 t, metal ores 52 t, nonmetallic minerals 10 t), Powertrain (fossil fuels 8 t, metal ores 21 t, nonmetallic minerals 7 t), Body (fossil fuels 4 t, metal ores 9 t, nonmetallic minerals 5 t). The largest contributors to total material footprint are fossil fuels (20.4 MT), copper ores (14 MT), iron ores (13.7 MT) and sand, gravel and crushed rock (11.7 MT). By component, material footprints are comprised mostly of Powertrain (12.9 MT), Body (7.5 MT), Transmission system (7 MT), Chassis (4.3 MT) impacts (see SI-7.1). Panels (iii) and (iv) show the region of impact for emissions and materials, respectively. The majority of production emissions occur domestically in Japan (53%), with remaining emissions occurring in China (15%) and the rest of the world. Unlike emissions, most material impacts occur internationally, as Japan performs little material extraction. The largest material impacts occur in Australia (12%), China (11%), Indonesia (10%), Chile (10%), Brazil (8%) and USA (5%).

**Figure 2:**
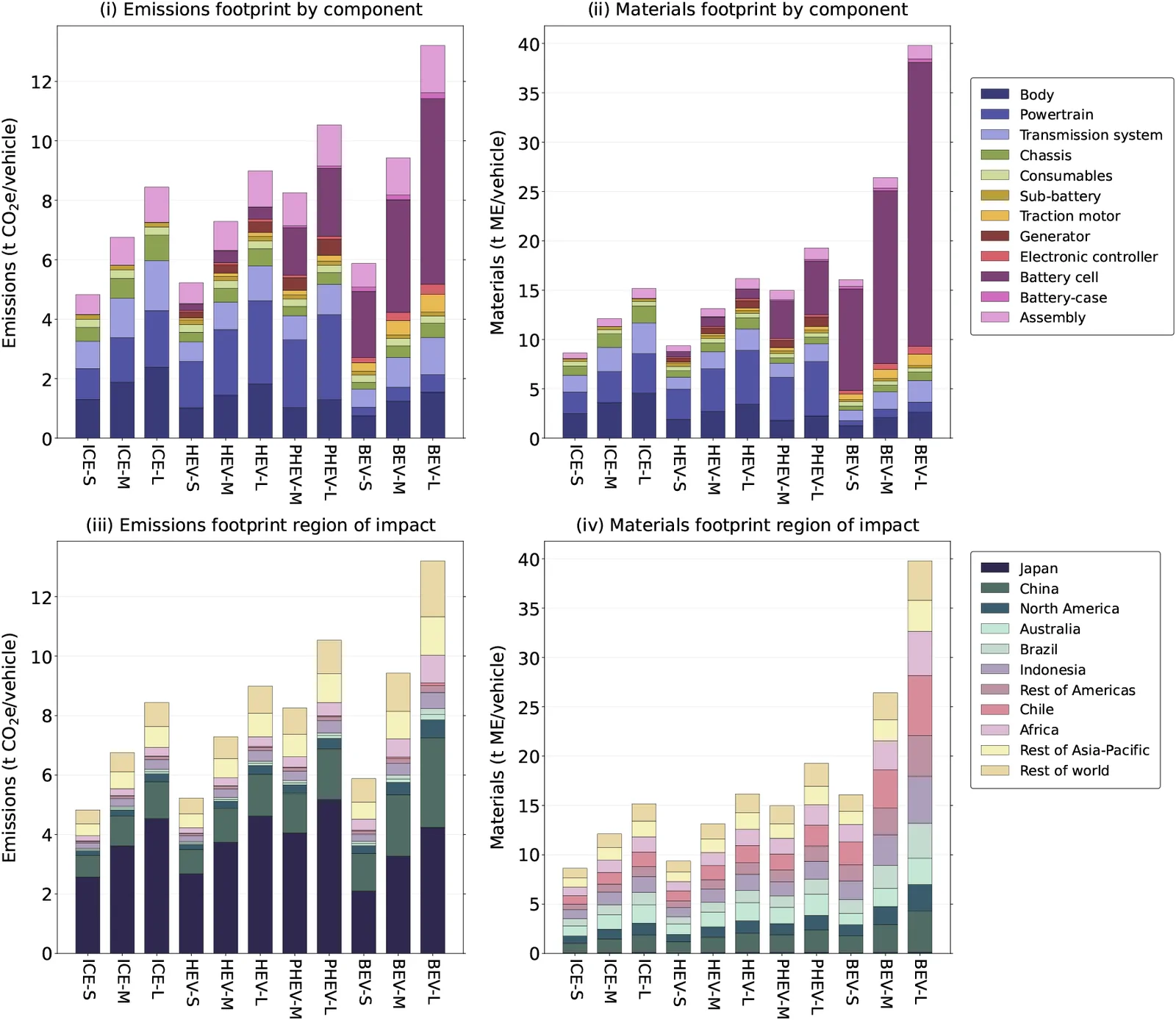
Production impacts by powertrain and vehicle size (S: small, M: medium, L: large). Panels (i) and (ii) emissions (t CO_2_e/vehicle) and material extraction (t/vehicle) footprints by component. Panels (iii) and (iv): emissions and material extraction footprint by region of impact.

In Figure 3, panels (i) and (ii) show impacts by production layer and input commodity for emissions and materials, respectively. Here *input commodity* refers to commodities entering Japanese vehicle production from both the domestic and global economy. Panels (i) and (ii) also show footprint completeness as the ratio between cumulative emissions and the complete footprint: ∑_*n*_ **qA** ^*n*^ **y**/*F*. These production layer decompositions (PLDs) were calculated to a depth of 8 production layers, accounting for 99% of total emissions and 97% of total material footprint. Panels (iii) and (iv) show cumulative emissions and material impacts by production layer and region of impact. The majority of emissions associated with electricity generation occurs in the first 3 layers (87%), consistent with electricity consumption by manufacturing industries in Japan. Service sectors impacts occur mainly in lower production layers, for example 75% of *repair and installation of machinery and equipment* impacts occur in the top 4 layers. Similarly, 80% of *professional, scientific and technical services* impacts occur by the fifth layer. Overall, vehicle parts and supporting industries contribute about 45% of total emissions, suggesting strong interlinkages and feedback loops within the vehicle sector itself and related suppliers. Approximately 5% of total emissions are generated by the *electrical equipment, precision instruments* and *machinery* sectors, occurring mainly in layers 2–6. These complex inputs are used in vehicles themselves as well as in manufacturing plants. See SI-7.3 for detailed PLD results by commodity. In panel (iv), material inputs from Chile (primarily metal ores) accrue relatively slowly, then become a leading contributor beyond layer 4. Similarly, while material extraction in Australia is significant in lower layers, this is surpassed by extraction in China beyond the fifth layer.

**Figure 3:**
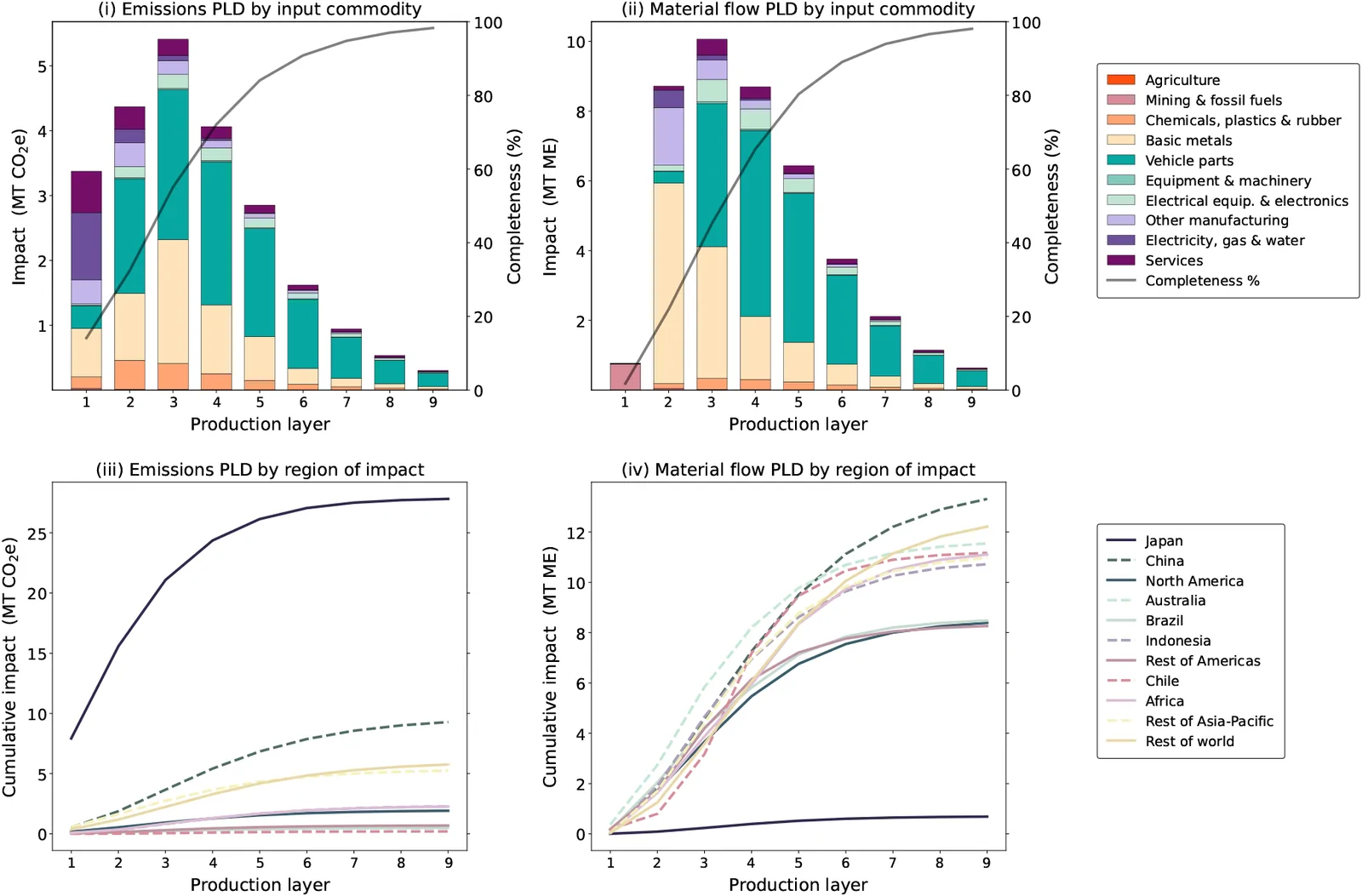
Production layer decompositions (PLDs) by (i) emissions by input commodity, (ii) material extraction (ME) by input commodity, (iii) emissions by region of impact, (iv) material extraction by region of impact. Panels (i) and (ii) also show cumulative footprint completeness with increasing production layer depth.

### 3.2 Lifecycle Impacts

Ranked lifecycle emissions by vehicle type and scenario are shown in Figure 4, with panels (i) and (ii) showing the contribution of each lifecycle stage to total emissions i.e., production, use (fuel or electricity grid) and maintenance. Panel (ii) shows the change in emissions resulting from lifetime extension, where maintenance impacts includes both regular repairs and additional maintenance. Panel (iii) plots emissions intensity (g CO_2_e/vehicle km) for each scenario. Future grid emissions intensity are shown for the no new policies (*static*) and IIEJ Advanced Technology (*AT*) scenarios. Two fuel scenarios are shown with upper and lower limits of *petrol* and biofuel blend *E20*. Two PHEV utilization factors are shown with lower bound of η = 0.75 and midpoint estimate η = 0.30.

**Figure 4:**
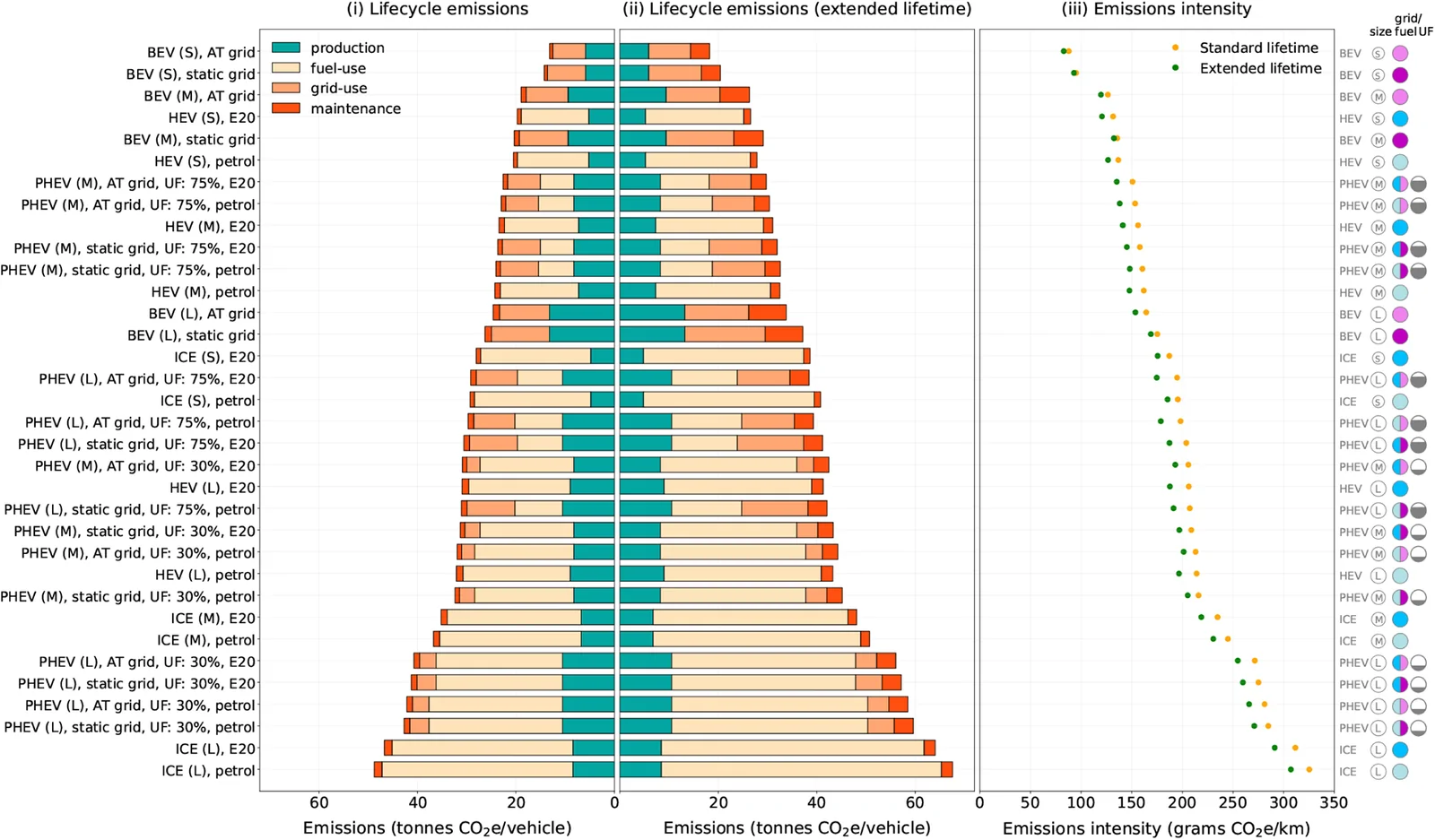
Ranked production and use-phase emissions by vehicle technology and use-phase scenario. Panel (i) shows lifecycle emissions, (ii) lifecycle emissions with extended lifetime and (iii) lifecycle emissions intensity. Lifetime extension: 15 to 22 years. E10 and E20: biofuel petrol blends, EM UF: electric motor utilization factor for PHEVs, grid scenarios: AT (IIEJ Advanced Technology) and static (no new policies). Vehicle sizes: small (S), medium (M), large (L). Note: all input parameter configurations are shown in SI-7.2. The scenario parameters are shown pictorially in the right-side legend, with UF shown in shaded gray circles and fuel/grid mix shown in shades of pink and blue, respectively.

Figure 4 shows top ranked vehicles are small and medium-sized BEVs and HEVs. Small-sized BEVs have lower impacts overall, regardless of the future grid emissions-intensity scenario. PHEVs with high UF approach the performance of BEVs, where other properties are equal. Similarly, low-UF PHEVs approach HEV performance though PHEVs must overcome higher production emissions than HEVs; therefore, to perform better than a HEV a low-UF PHEV must utilize low grid emissions intensity during their lifetime. Large-sized PHEVs also perform worse than medium sized ICVs, this is due to higher production emissions and vehicle weight. Vehicle size is a strong determinant of performance, for example large-size PHEVs, HEVs and ICVs all perform poorly, regardless of other scenario parameter values. Conversely, small-sized HEVs and ICVs perform better than many medium sized PHEV scenarios.

In panel (ii), vehicle lifetimes are extended from 15 to 22 years (150,000 km to 220,000 km). For ICVs, HEVs and PHEVs, lifetime extension adds 32–37% extra emissions to the lifecycle footprint, and 40% for BEVs. Lifetime extension decreases the emissions intensity (gCO_2_e/km) of all vehicles by an average 7% (ICV: 6.3%, HEV: 8.8%, PHEV: 10.2%, BEV: 4.5%), shown in panel (iii). Lifetime extension also causes a reordering of rankings, for example *HEV (S), petrol* and *BEV (M), static grid* switch places as the replacement battery disadvantages the BEV. For PHEVs and BEVs, lifetime extension improves the performance of BEVs and PHEVs size for the grid AT scenario (clean grid), since the longer lifetimes combine with progressively lower future grid emissions intensity. However, this effect is dampened by the required battery replacement, resulting in extra maintenance emissions and relatively lower intensity reductions. In the final year of the lifetime extension scenario (2041), AT grid emissions intensity is 20% lower than the static scenario, though the cumulative mean intensity differs by only 11% (this relatively slow divergence can be seen in SI-4.3). The implication of this is that the differences between electric and combustion powertrains will grow if the grid continues to decarbonize. A cleaner grid will also accentuate the higher performance of high-UF over low-UF PHEVs.

### 3.3 Mobility Service Interventions

The cumulative abatement potential of mobility service interventions are plotted in Figure 5. Strategies are grouped by color, with strategy variations shown in lighter shades. Total delivered mobility services (pkm), grid emissions intensity and PHEV utility factor are all held constant across the scenarios (UF: 0.6, grid: *IEEJ reference*). The standard lifetime is 15 years, with 10,000 km traveled per year, with the option to extend the vehicle life to either 22 or 30 years. Additional material requirements for repair and maintenance are spread over the vehicle lifetime. Fuel strategies are shown to contrast the impact of mobility strategies. Here strategies are applied cumulatively to control for interaction effects.

**Figure 5:**
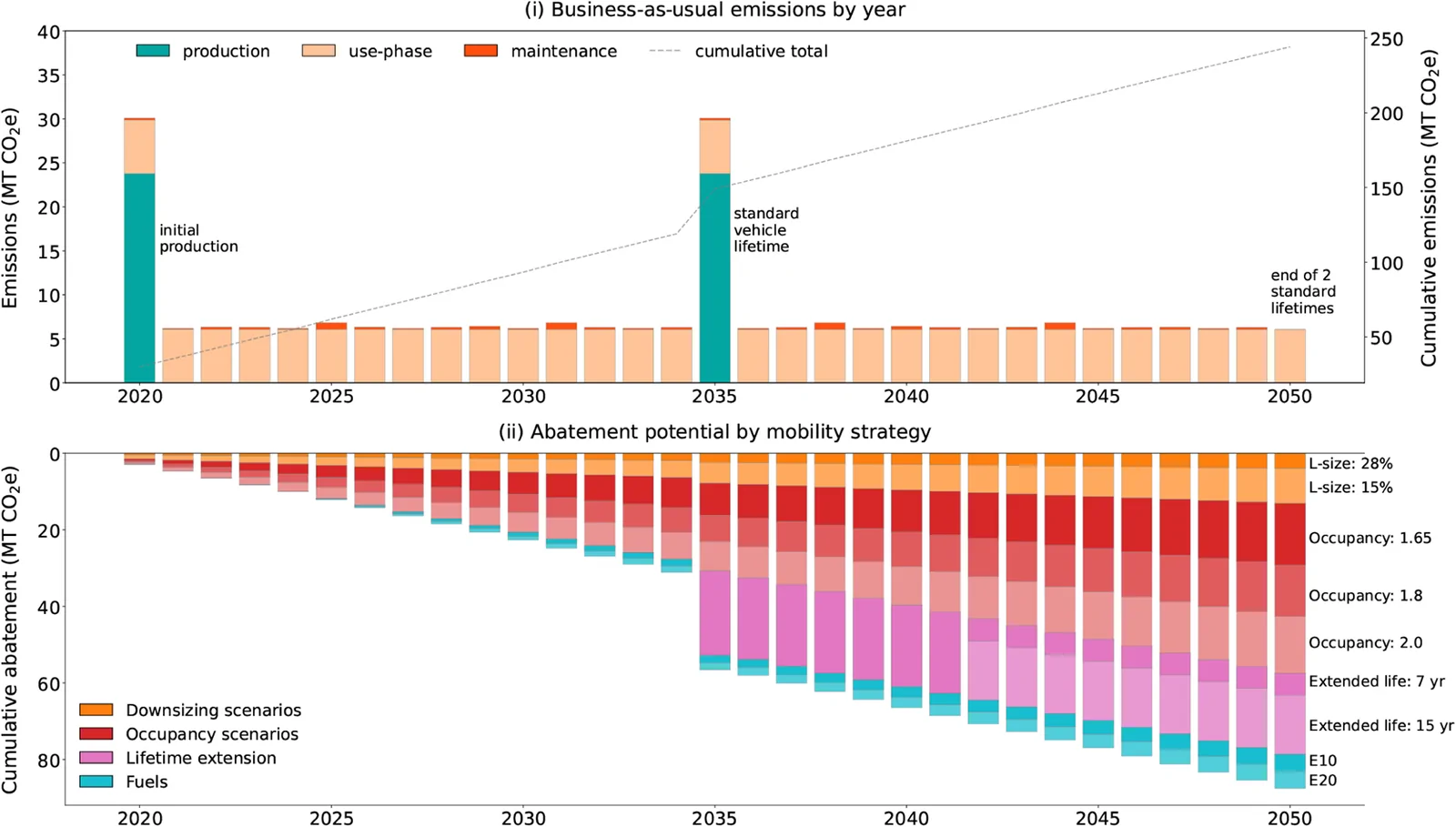
Panel (i) emissions by year for BAU case, with each emissions source in color and cumulative emissions plotted on the secondary axis. Use-phase includes both fuel and electricity emissions. Panel (ii) cumulative abatement potential by mobility service strategy. Mobility services (pkm) are constant across strategies. L-size: fraction of large-size vehicles in cohort, compared to 33% baseline. Occupancy: people per vehicle, compared to 1.5 persons/vehicle baseline. Extended lifetime: beyond standard lifetime of 15 years.

Total business-as-usual emissions from 2020 to 2050 are on the order of 237 MT CO_2_e. Applying all mobility strategies together represents an abatement potential of about 80 MT or 33%. Downsizing improves fuel economy and lowers production impacts though lower material requirements and reduced vehicle curb weight (e.g., a medium ICV is 20% lighter than the large size). The downsizing strategies show that if the fraction of large vehicles grows from current levels of 28% to 33%, then emissions increase by 1.6%. Conversely, reducing the fraction of large vehicles to 15% reduces emissions by 4%. Increasing vehicle occupancy decreases use-phase emissions by reducing vehicle-kms in addition to reducing vehicle production requirements. Increasing occupancy from 1.5 to 2.0 occupants per vehicle could result in emissions reductions of 20%.

Lifetime extension is also significant; extending vehicle lifetimes to 15 years results in a 15% emissions reduction by avoiding production of new vehicles. Lifetime extension requires further material inputs with associated impacts, however these impacts are repaid over time resulting in overall decarbonization. The main benefits of 7-year lifetime extension accrue in the first 7 years, after which further production is required to ensure in-use vehicles match the mobility requirements. However, gains from 7-year lifetime extension are not totally forfeited since a reduced number of vehicles is required to keep mobility constant (see SI-5.4 for more detail). The benefits of lifetime extension are most apparent when the temporal window is extended to include multiple vehicle lifetimes and the wider mobility task. Lifetime extension of 15 years fits within the interval 2035–2050 and therefore no further production of vehicles is required. Reductions from 15-year lifetime extension begin in 2035 however this is partially obscured in the figure.

### 3.4 Embodied Emissions Paths

The ≈150,000 top embodied emission paths into vehicle production were indexed, accounting for ≈51% of all emissions. Iron and steel paths into engines, vehicle body and chassis dominated these top paths, for example *Hot rolled steel (JPN)* < *Crude steel (converters) (JPN)* < *Pig iron (JPN)*. Among the top paths, Scope 1 and 2 emissions associated with vehicle assembly are also prominent, highlighting the opportunity to target these emissions through efficiency measures in vehicle factories and on-site renewable generation. Freight transport, glass production, and plastics manufacturing, also contribute to the top paths.

Top paths are shown by industry and region in Figure 6 panel (i). The paths are overlaid in a graph representation with each cell showing the cumulative impacts through that node. Here the impact values are summed along the path until terminating in the Japan vehicle sector. Approximately 12,000 paths were edited from the pool of top paths, resulting in reductions of 0.5 MT or 2.5% of all vehicle production emissions, though this search was not exhaustive. These edits are visualized in panel (ii), where reductions are shown in pink and increases in green. Note that the search constraints (section 2.8) result in no edited paths with international connections beyond one layer removed from Japan. Significant edits include moving aluminum smelting from China to Canada, where the energy supply is low emissions hydropower, and shifting nonferrous metals smelting from South Africa to Germany, where the grid emissions intensity is lower. The top 50 embodied emissions paths and substitutions are shown in SI-6.4.

**Figure 6:**
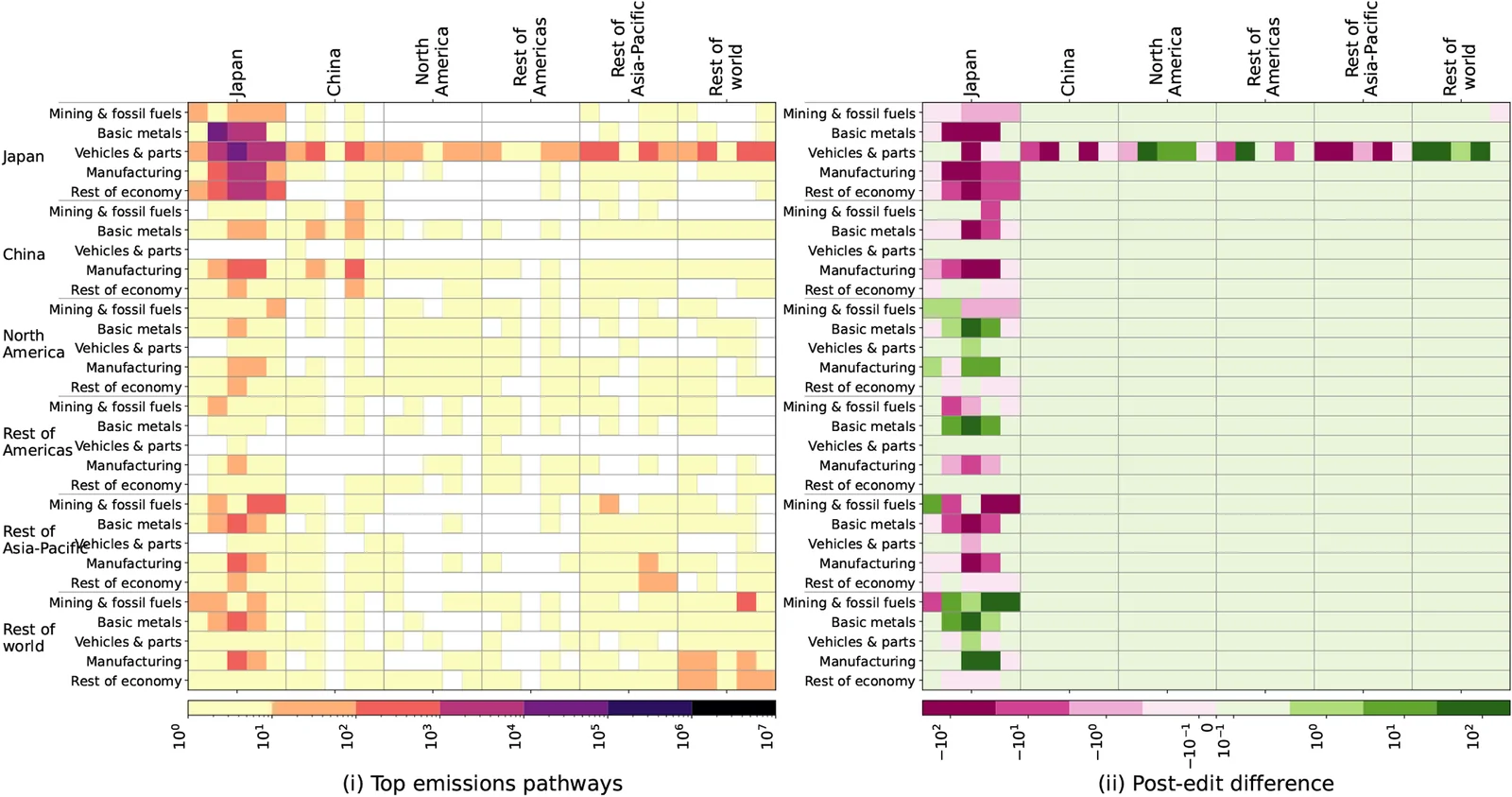
Graph representation of embodied emission paths. Panel (i) heatmap of SPA nodes where color is proportional to the path value (kg CO_2_e). Panel (ii) heatmap of path edits (kg CO_2_e), where reductions are shown in pink and increases in green. Paths are overlaid so values are the sum of embodied impacts through each node.

## 4 Discussion

This study assessed emissions and material footprints of vehicle production and consumption in Japan, considering both individual vehicles, the vehicle cohort and domestic vehicle production sector. For medium sized vehicles, we found production emissions, on the order of ICV: 6.8 t, HEV: 7.3 t, PHEV: 8.3 t, BEV: 9.4 t (CO_2_e). These findings are comparable with^14^ ICV: 6.5 t, HEV: 7.4 t, PHEV: 10 t, BEV: 9.8 t, though somewhat higher than:^72^ ICV: 4.1 t, HEV: 5.1 t, BEV: 8.6 t. This study found emissions intensity (gCO_2_e/km) for ICVs: 186–321, HEV: 132–206, PHEV: 150–215, BEV: 90–156, which are comparable with results of^73^ for ICVs: 240–310, HEV: 150–200, PHEV: 180–190, BEV: 130–170 (gCO_2_e/km). There are several possible sources for these differing estimates, for example differences in vehicle size definitions, whether auxiliary services to vehicle production were included, the domestic vehicle production sector efficiency and grid emissions intensity. The sensitivity of the production footprint to battery capacity for PHEVs and BEVs is on the order of ± 6–21% (95% CI). Commodity price sensitivity was also tested and found to alter the production footprint by ± 6–14% (95% CI) (see SI-8 for more detail).

A mixed-unit nested MRIO was employed in this study to estimate environmental impacts arising from vehicle production and consumption in Japan. Official Japanese IO tables for 2020 were used, the latest official tables at the time of writing. Our study results may therefore be improved through using the 2025 tables, once available, as well by capital endogenisation techniques, which capture variation in capital expenditure by the vehicle production industry over time (see for example ref 74). While global MRIOs account for variation in national impact intensities, these intensities can also vary within-countries.^75^ For example, emissions intensity varies by regional renewable energy availability, and biodiversity threats are often heterogeneous. This can be addressed by nesting a subnational MRIO within a global MRIO, so that spatial variance in impacts and consumption is better represented.^76^ Another approach is through spatially explicit LCA, for example linking LCA and high-resolution crop production databases to improve biodiversity footprints.^77^

Impacts from future materials production were estimated using existing technology, however Integrated Assessment Models (IAMs) could be used to calculate these impacts using future technology. Iron and steel production are significant contributors to vehicle production impacts and while alternative technologies exist, such as green hydrogen and carbon capture, it is not clear when, or if, these technologies will be deployed at scale. If decarbonization occurs in these and other industries, future impacts associated with maintenance and lifetime extension are likely overestimated. New battery chemistries, such sodium-ion, were not considered in this study but have the potential to reduce future battery impacts. In this study, vehicles imported to Japan and exported from Japan were not considered, neither were diesel or hydrogen fuel-cell vehicles (which represent ≈2% of new sales). While supply chain impacts from electricity generation were included, charging infrastructure for electric vehicles were not covered. Downstream impacts, i.e., from waste disposal and treating EV batteries, were also not included.

This study found that lifetime extension can reduce emissions by ≈15% over 15 years at the cohort level, and has the potential to reduce vehicle emissions intensity by 4.5–10%. This result is comparable to^78^ who found a 5% reduction from lifetime extension over 5 years. The material footprint of vehicle maintenance is 30–35% of the vehicle footprint for ICV and HEV vehicles, aligning with^79^ who found maintenance contributes 30% to total material requirements. The long-term availability of spare parts is important for the practical feasibility of lifetime extension. Repair programs can ensure spare parts are available to consumers long-term and provide ongoing revenue to vehicle manufacturers. Manufacturers should design-for-repair and avoid the planned obsolescence of components. Standardizing components across models, and between manufacturers, can reduce the cost of maintenance and the availability of spare parts.^80^ Given the material intensity of PHEVs and BEVs, applying circular economy principles to these vehicle lifecycles is critical. In this study, lifetime extension of PHEVs and BEVs was modeled as requiring traction battery replacement, though the lifetime of these batteries is currently uncertain.^81^ Once EVs reach end-of-life, battery recycling and cascading use can be employed, for example by repurposing EV batteries in community energy projects.

The combination of mobility strategies has the potential to reduce emissions by 30% and several demand-side policy options exist to realize these reductions. Japan has existing policy incentives to favor smaller vehicles, for example the *vehicle weight tax* scales with vehicle size,^43^ however the share of larger vehicles is increasing suggesting stronger incentives are required. The weight tax increases once vehicles reach 13 years old, which may partially explain the comparatively shorter vehicle lifetimes in Japan. In addition, consumers may prefer newer vehicles over higher repair and registration costs for older vehicles.^82^ This study focused on vehicle use in Japan, thus exports of secondhand vehicles were excluded, consistent with consumption-based accounting methods. However, many secondhand vehicles are exported to foreign markets from Japan, which may shorten vehicle lifetimes in Japan and stimulate demand for vehicles in the importing country. Raising export duties on vehicles below an age threshold, e.g., fifteen years, may discourage this. Other demand-side policies include subsidizing public transport (decreasing vehicle kilometers) and discouraging households from owning multiple vehicles (increasing occupancy).

Capturing all significant contributions to the footprint requires including higher-order production layers; this study found almost half of emissions and most material extraction impacts occur outside Japan. We searched embodied emissions pathways for decarbonization potential and discovered opportunities on the order of 2.5% of vehicle production emissions. This substitution of embodied emissions pathways should be interpreted carefully, since determining possible input substitutions is difficult, even using detailed HS commodity codes. Producers in Japan should target these overseas hot-spots and engage with suppliers to reduce upstream impacts.^83^ The material requirements of BEVs and PHEVs are much higher than conventional vehicles and to avoid the substitution of emissions for other impacts, such as biodiversity loss and deforestation, Japan could adopt legislation requiring responsible extraction, for example aligning with the EU Deforestation Regulation (EUDR).^84^ Vehicle production may also be exposed to instances of forced labor and modern slavery, hence producers should also audit upstream supplier labor practices (this can also be legislated.^85^

BEVs and PHEVs currently receive substantial financial support in Japan, such as purchase subsidies and tax incentives during vehicle inspections. Older BEVs and PHEVs also remain exempt from additional taxation as long as they meet fuel-efficiency standards. In addition, existing BEV subsidy criteria include energy efficiency and single-charge driving range. The effect of vehicle size on lifecycle impacts is strong, hence incentives should be focused on small and medium-size BEVs and PHEVs. PHEV impacts are sensitive to real world driving patterns, specifically the electric motor UF, hence incentives for PHEVs should be based on actual operation. Additional policy options for PHEVs include setting a floor for the range on a single charge, since a greater charge capacity enables higher UFs, and capping the real-world emissions intensity.^86^ The availability of charging infrastructure, for example in apartment buildings, workplaces and parking facilities, also affects EV uptake and should be encouraged.

While grid decarbonization may reduce production and in-use emissions for PHEVs and BEVs, Japan is heavily reliant on fossil fuels and it is unknown when the energy grid will decarbonize. Further decarbonization of the energy grid and vehicle electrification can reduce both reliance on fossil fuel imports and transport emissions. A conservative modeling approach was taken by this study to model biofuels by setting a maximum of 20% bioethanol blend (E20). While it is possible to burn higher blends in combustion engines, modeling biofuel consumption is fraught since biofuel demand impacts food crops, afforestation and competes with fuel use in other sectors. Nevertheless, domestic biofuel production may have a role in reducing fuel impacts and fuel import supply risk.

For Japan’s transport sector to achieve net-neutrality by 2050, strong action is required. Demand-side mobility strategies are particularly important since these strategies can reduce emissions without relying on technology, such as CCS and hydrogen, which may not arrive or scale. Furthermore, our path analysis shows that current upstream emissions reduction opportunities are limited, and no low-pollution production havens exist. Mobility strategies also link transport policy with social policy; access to mobility is social capital and a means of maintaining social networks, particularly in regional and depopulated areas.^87^ To maintain access in labor-constrained regions, mobility may need to be flexibly provided through smaller vehicles, demand-responsive and reservation-based services. Emergent technologies such as self-driving vehicles hold promise, for example to address driver shortages, though uncertainty remains about the effectiveness.^88^

## Supplementary Material



## Notes

MRIO model structure; vehicle component data; system boundary definition; use-phase parameters; mobility scenario detail; structural path analysis; extended results; sensitivity analysis (PDF)
