# Supplementary material for: Decomposing Vehicle Production and Consumption Impacts in Japan under Mobility and Production Network Interventions
Source: Environ Sci Technol. 2026 Sep 13;60(37):26357–69. doi: 10.1021/acs.est.6c06919 (PMC13614184; doi:10.1021/acs.est.6c06919)
Supplement: Supplementary file 1 [file es6c06919_si_001.pdf]

Supporting Information

Decomposing vehicle production and consumption impacts in Japan under mobility and production network interventions

Jacob Fry, Ziyan He, Hiroyuki Ishikawa, Takamichi Iwata, Minami Kito, Kenichi Nakajima, Keisuke Nansai

Contents

SI-1 Japan vehicle data **S2**
SI-1.1 Compiling component data . . . S2
SI-1.2 Vehicle component weight . . . S3
SI-1.3 Japan vehicle flows . . . S3

SI-2 MRIO model structure **S5**
SI-2.1 Nested MRIO . . . S5
SI-2.2 Hybrid process-MRIO interface . . . S6
SI-2.3 Non-ferrous metals disaggregation . . . S7
SI-2.4 Hybrid model structure . . . S8
SI-2.5 GLORIA country definitions . . . S9
SI-2.6 GLORIA sector definitions . . . S10

SI-3 System boundary **S11**

SI-4 Use-phase parameters **S12**
SI-4.1 Vehicle fuel economy . . . S12
SI-4.2 Other assumptions . . . S13
SI-4.3 Grid electricity emissions intensity . . . S14

SI-5 Mobility scenarios **S15**
SI-5.1 Mobility services and emissions . . . S15
SI-5.2 Occupancy . . . S15
SI-5.3 Downsizing . . . S16
SI-5.4 Lifetime extension . . . S16
SI-5.5 Mobility segment and vehicle age cohorts . . . S17

SI-6 Path analysis **S18**
SI-6.1 IO decompositions . . . S18
SI-6.2 Path substitution . . . S18
SI-6.3 Trade similarity . . . S18
SI-6.4 Embodied emissions and material structural paths . . . S20

SI-7 Extended results **S23**
SI-7.1 Material footprints . . . S23
SI-7.2 Lifecycle emissions . . . S24
SI-7.3 Production Layer Decompositions . . . S25
SI-7.4 GLORIA and nested MRIO comparison . . . S27

SI-8 Sensitivity analysis **S28**
SI-8.1 Battery capacity . . . S28
SI-8.2 Comtrade prices . . . S29

References **S32**

SI-1 Japan vehicle data

SI-1.1 Compiling component data

SI-1.1.1 Vehicle definitions

The powertrains of the vehicles covered are gasoline vehicles (ICEV), hybrid vehicles (HEV), plug-in hybrid vehicles (PHEV), and electric vehicles (BEV). Material composition data for each of the four powertrains listed above was prepared for large, medium, and small vehicles. Specifically, we assumed that the large vehicle would be a D-segment SUV, the medium-sized vehicle would be a C-segment sedan, and the small vehicle would be an A-segment minicar. Data for the small plug-in hybrid vehicle (PHEV) was not prepared because there are currently no vehicles for sale that fit its category.

SI-1.1.2 Vehicle material composition

The actual vehicle types that match the definitions of large, medium and small vehicles explained in (1) were picked out, and the weight of those vehicle types was used. From the actual weight of the battery for each vehicle type was excluded, and the weight of the vehicle excluding the battery was calculated. The weight of each part that makes up the vehicle and the proportion of each material that makes up each part (material composition ratio) was taken from the GREET 2023 database (Wang et al., 2023). We have confirmed that there is no significant difference between the weight of each material used in actual vehicles and the values calculated based on the material composition ratios in GREET.

In GREET, the entire vehicle (excluding the battery) is divided into the following parts: body, powertrain system, transmission system, chassis, traction motor, generator, and electronic controller. The weight ratio of the materials that make up each part is listed. The materials include iron, aluminum wrought products, aluminum extruded products, copper, glass, plastics, and so on. For example, the steel used in the body is often hot-dip galvanized steel, while the steel used in the powertrain is often ordinary steel. Even if it is the same type of steel, the manufacturing process and alloy components added to it differ. For this reason, we identified the industrial classification in the Japanese input-output table for each material used in each part. This work was carried out based on the knowledge of engineers.

SI-1.1.3 Battery material composition

For hybrid electric vehicles (HEVs), plug-in hybrid electric vehicles (PHEVs) and battery electric vehicles (BEVs), the weight of the battery cells used in actual vehicles and the weight of the entire battery pack were used as references to calculate the weight of each type of vehicle: large, medium and small. The type of battery was assumed to be NMC (111), and the weight ratio of the materials that make up the battery was set based on values published by Park et al. (2021) and engineering knowledge.

The weight of each element contained in the battery does not match the weight of the compounds used in manufacturing the materials, because the Ni, Mn, and Co used in the positive electrode are sulfides, and the Ni is lithium carbonate. Therefore, we estimated the weight of each compound used to manufacture the battery using the ratio of the atomic weight of each element to the molecular weight of each compound. However, the material yield rate was assumed to be 100%.

SI-1.1.4 Maintenance and repair

We identified materials and vehicle components likely requiring replacement during the vehicle lifetime, and the replacement intervals. The requirements of consumeables, such as oil, fluids and tyres, were also estimated. Sub-battery replacement was included for all vehicles, however EV traction batteries were not included in conventional maintenance, as these were deemed to last for the standard vehicle lifetime. The material requirements were estimated by powertrain and linearly scaled by vehicle size. Maintenance and repair settings are configured by referencing the maintenance information provided by the vehicle manufacturer for normal operating conditions (neither severe using conditions nor excessive driving mileage).

SI-1.1.5 Lifetime extension

In addition to the materials required for conventional maintenance, we also identified materials required to increase the lifespan of the vehicle beyond 15 years. The replacement interval for lifetime

extension materials was parameterised to enable scaling with various lifetime extension choices. Lifetime extension materials include vehicle components made of metal, glass, rubber and plastics in the vehicle body, powertrain, transmission and chassis. Also included are materials required to preserve the interior of the vehicle, such as plastics and rubber. The material requirements were estimated by powertrain and linearly scaled by vehicle size. Maintaining a vehicle's functionality and preserving its value extends the service life of passenger cars. The quantity of parts required to maintain a vehicle's functionality is calculated based on repair and replacement data held by the vehicle manufacturer. Parts replacement to maintain value focused on interior components that passengers might touch, specifically those containing a high proportion of resin—a material that degrades more quickly than metal—and the seats.

SI-1.2 Vehicle component weight

Vehicle component weights by powertrain and size:

Table 1: Vehicle component weights (kg)

technology	size	body	chassis	powertrain	transmission system	battery cell	battery-case	electronic controller	generator	traction motor	total
ICV	small	472	229	138	57	0	0	0	0	0	896
ICV	medium	682	330	199	82	0	0	0	0	0	1294
ICV	large	866	419	253	104	0	0	0	0	0	1642
HEV	small	414	201	209	46	10	10	16	20	19	945
HEV	medium	585	284	296	65	18	17	22	28	27	1343
HEV	large	741	359	375	83	18	17	28	35	34	1691
PHEV	medium	575	278	325	64	70	120	24	44	43	1542
PHEV	large	723	350	408	81	99	140	30	55	54	1940
BEV	small	430	208	39	48	99	140	49	0	78	1091
BEV	medium	710	344	65	79	169	160	81	0	129	1736
BEV	large	885	429	80	99	278	200	101	0	160	2232

SI-1.3 Japan vehicle flows

Estimates of Japanese vehicle production, imports, exports and domestic demand, estimated by combining IEA (2024); JAMA (2024); WTO (2024) datasets:

Domestic production:

	ICE	HEV	PHEV	BEV	FCV	CleanDiesel
**SUV**	590,806	244,567	12,321	15,199	84	33,882
**large**	1,477,014	413,883	20,850	22,352	188	75,943
**medium**	1,358,853	376,258	18,955	11,623	72	29,063
**small**	2,185,981	808,954	0	39,339	76	30,795

Table 2: Japanese passenger vehicle production, by technology and size (count of vehicles)

Imports:

	ICE	HEV	PHEV	BEV	FCV	CleanDiesel
**SUV**	21,106	8,737	440	543	3	1,210
**large**	52,766	14,786	745	799	7	2,713
**medium**	48,545	13,442	677	415	3	1,038
**small**	78,094	28,900	0	1,405	3	1,100

Table 3: Passenger vehicle imports to Japan, by technology and size (count of vehicles)

Exports:

	ICE	HEV	PHEV	BEV	FCV	CleanDiesel
**SUV**	531,396	198,926	9,613	11,867	65	26,188
**large**	1,328,490	336,643	16,268	17,451	145	58,698
**medium**	1,076,735	293,159	14,672	8,998	56	22,463
**small**	23,070	3,159	0	55	0.059	24

Table 4: Passenger vehicle exports from Japan, by technology and size (count of vehicles)

Domestic demand:

	ICE	HEV	PHEV	BEV	FCV	CleanDiesel
**SUV**	80,516	54,379	3,148	3,875	22	8,904
**large**	201,290	92,026	5,327	5,699	49	19,958
**medium**	330,663	96,540	4,960	3,040	19	7,638
**small**	2,241,005	834,694	0	40,689	79	31,871

Table 5: Japanese passenger vehicle domestic demand, by technology and size (count of vehicles)

SI-2 MRIO model structure

SI-2.1 Nested MRIO

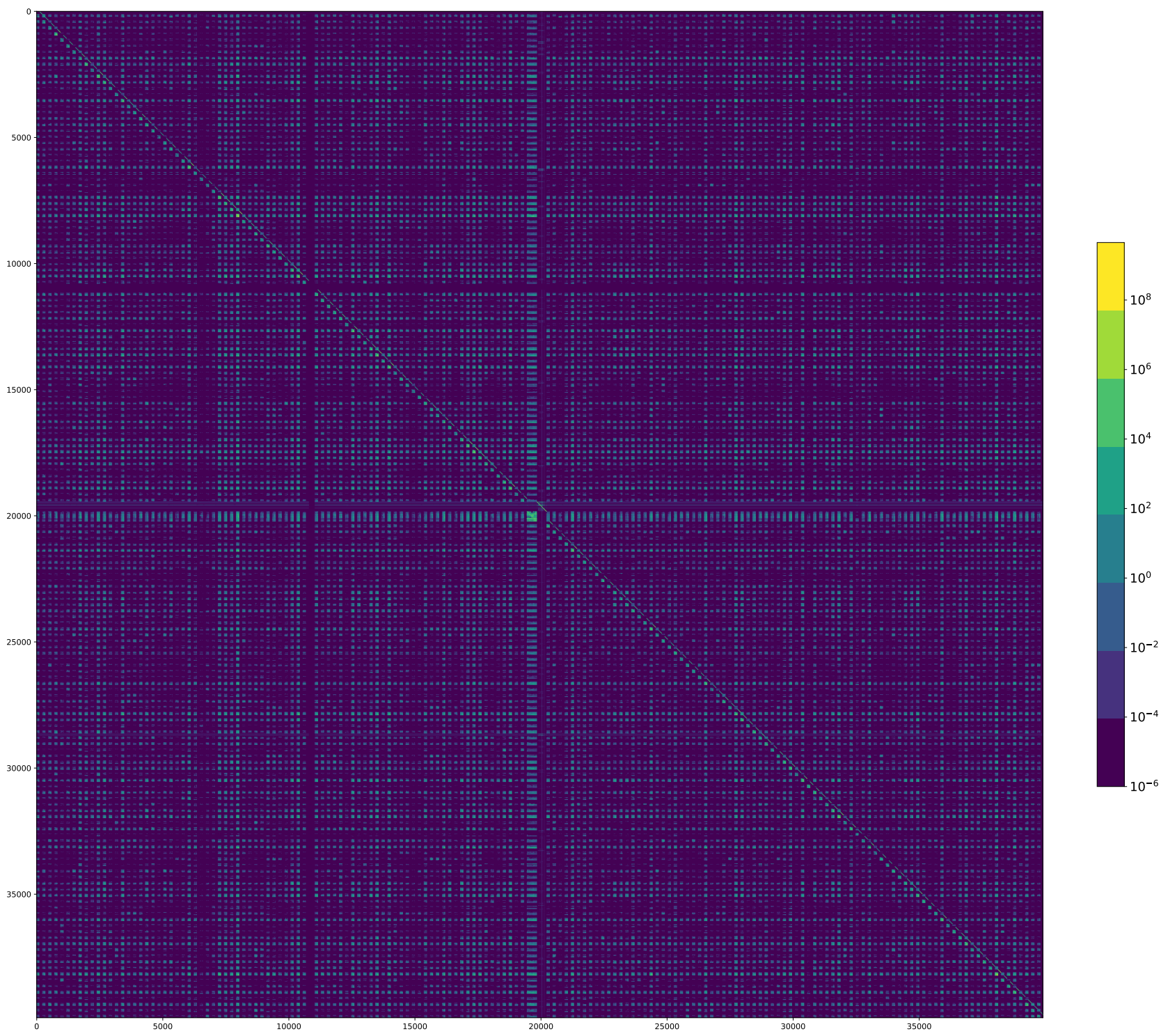

Figure 1: Japan-global nested MRIO (000s of USD). Colour scale is proportional to the log of 000s of USD.

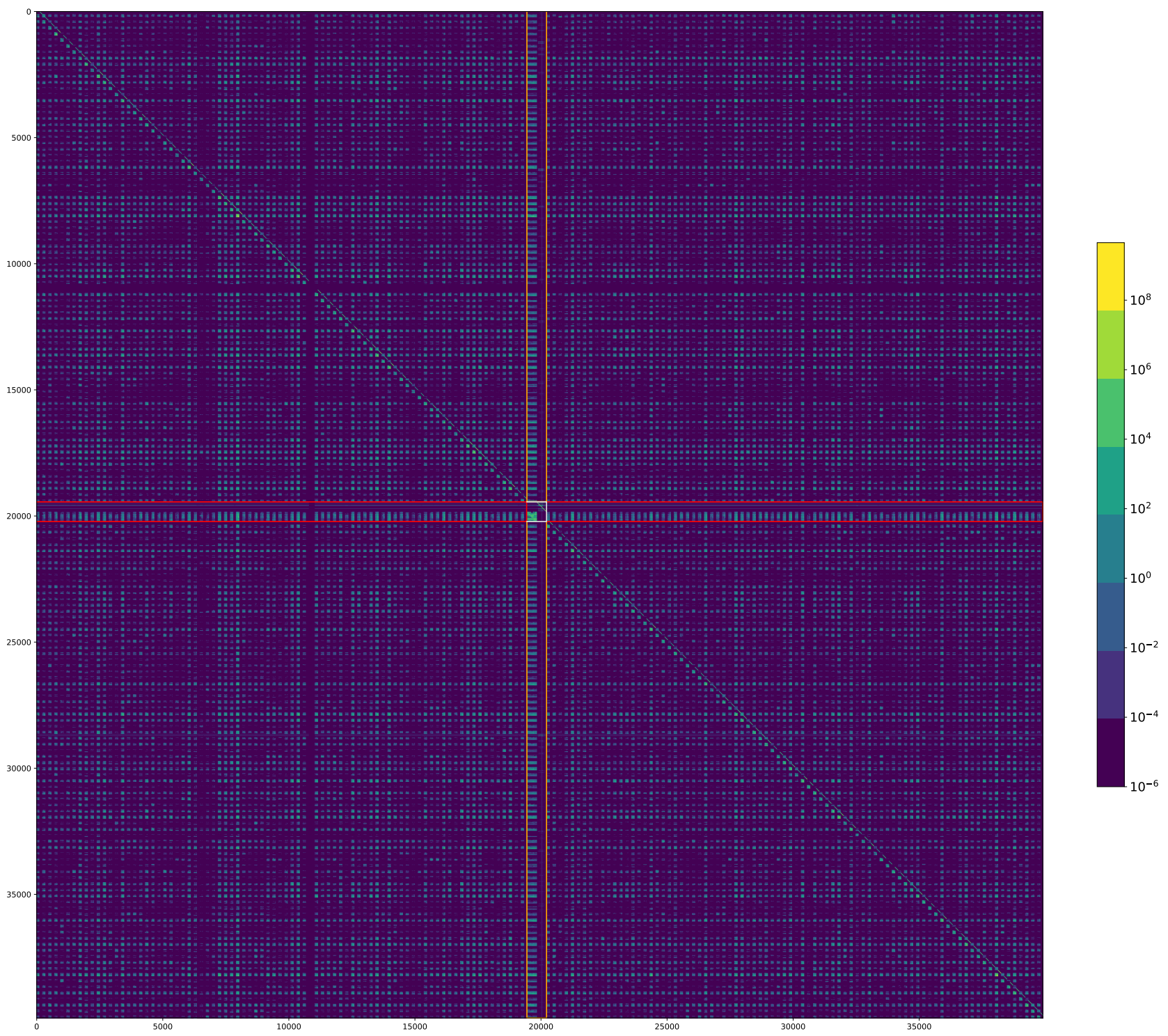

Figure 2: Japan-global nested MRIO (000s of USD). Imports to Japan are outlined in orange and exports from Japan outlined in red. Colour scale is proportional to the log of 000s of USD.

SI-2.2 Hybrid process-MRIO interface

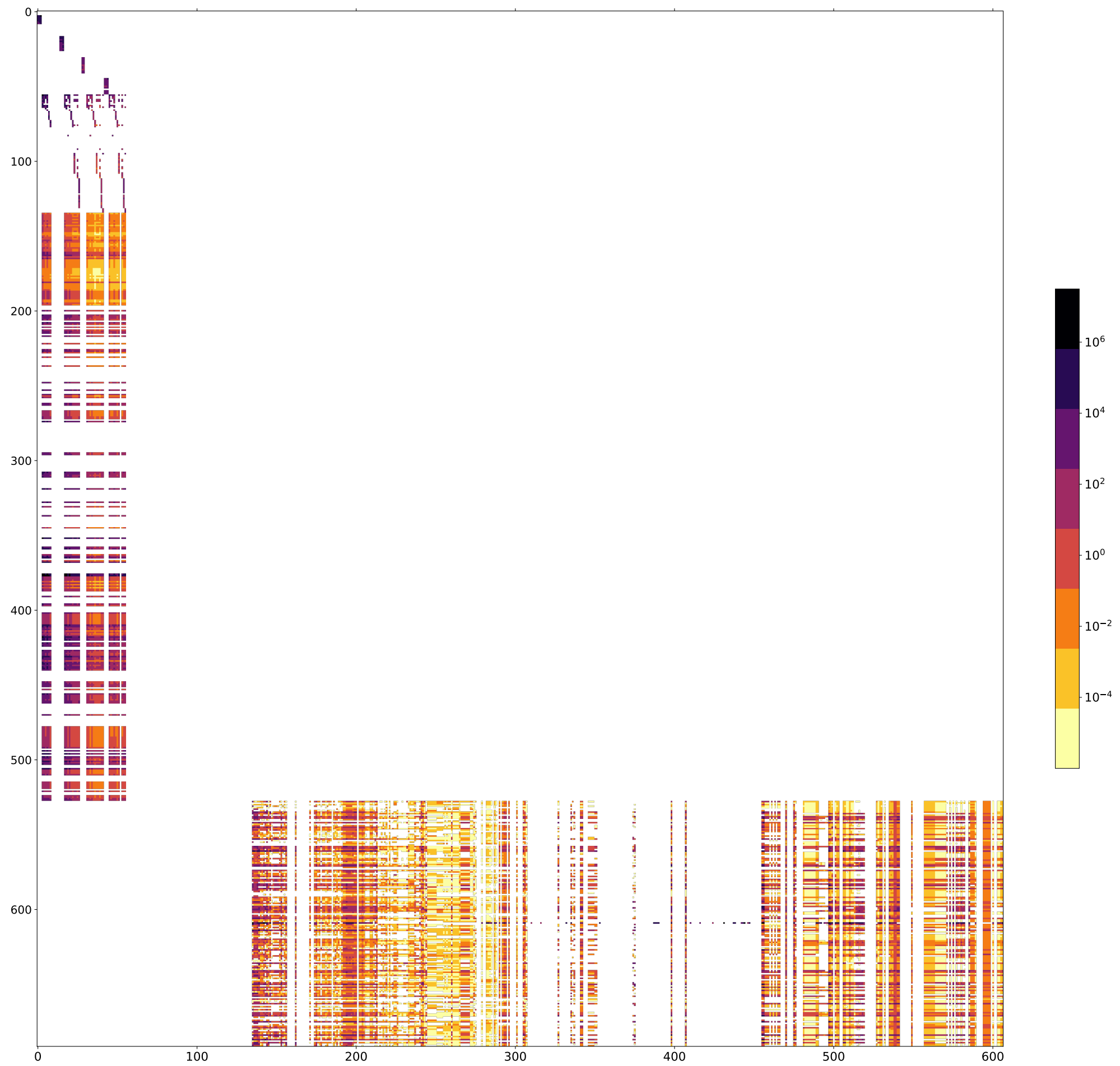

Figure 3: Hybrid MRIO simplified heatmap.

SI-2.3 Non-ferrous metals disaggregation

Non-ferrous metals are a significant input to the vehicle manufacturing industry, for example aluminium bodies and copper in electronics. However, there is poor alignment between the non-ferrous metal categories in the Japan IOT and GLORIA, for example *nickel*, *silver* and *tin* are aggregated in the Japan IOT. Therefore we disaggregate the Japanese non-ferrous metals category to prevent aggregation error at the interface between these two models. The supply of these non-ferrous metals is disaggregated using production data (METI, 2020) while use of metals is allocated using the WIO-MFA dataset (Nakamura and Nakajima, 2005; Ohno et al., 2016).

		GLORIA						
	**Pre-disaggregation**	Basic aluminium	Basic Copper	Basic Gold	Basic lead/zinc/silver	Basic nickel	Basic tin	Basic non-ferrous metals n.e.c.
**Japan**	Copper	0	1	0	0	0	0	0
	Lead and zinc (including regenerated lead)	0	0	0	1	0	0	0
	Aluminum (including regenerated aluminum)	0	0	0	0	0	0	0
	Miscellaneous non-ferrous metals	0	0	1	1	1	1	1

	**Post-disaggregation**	Basic aluminium	Basic Copper	Basic Gold	Basic lead/zinc/silver	Basic nickel	Basic tin	Basic non-ferrous metals n.e.c.
**Japan**	Copper	0	1	0	0	0	0	0
	Lead and zinc (including regenerated lead)	0	0	0	1	0	0	0
	Aluminum (including regenerated aluminum)	0	0	0	0	0	0	0
	Nickel	0	0	0	0	1	0	0
	Silver	0	0	0	1	0	0	0
	Tin	0	0	0	0	0	1	0
	Miscellaneous non-ferrous metals	0	0	1	0	0	0	1

Figure 4: Alignment between Japan and GLORIA non-ferrous metal categories, before and after disaggregation

metal	production	imports	exports
antimony	0	5027118	158320
cobalt	0	9165807	0
mercury	0	8	26082
molybdenium	692201	836659	272509
nickel	58778393	53544706	20896191
nickel-alloy-and-scrap	255893717	13003401	7482402
silver	1783098	1822405	4659095
tin	1547091	24283136	593307
tin-scrap	837692	46122	197514
tungsten	3904324	1133354	70140

Table 6: Non-ferrous metal physical flows from METI (kgs)

SI-2.4 Hybrid model structure

Table dimensions: vehicle technologies: k (*ICE, HEV, BEV, PHEV*), vehicle sizes: z (*small, medium, large*), components: h (e.g. *body, powertrain, transmission*, etc.), materials: m (e.g. *steel*, *glass*, *polyurethane*, etc.), regions r (Japan) and s (all remaining global regions defined in GLORIA), industries: i, commodities: c, a: vehicle manufacturing sector.

$$
\mathbf{y}_{\text{hyb}} =
\begin{array}{lll}
 & & \\
 & k_1 & z \\
 & & h \\
p & k_2 & z \\
 & & h \\
 & & c \\
r & & i \\
 & & c \\
s & & i \\
 & & c
\end{array}
\begin{array}{c}
\begin{array}{ccc} p & r & s \end{array} \\
\left[\begin{array}{cc}
\multicolumn{2}{c}{\mathbf{y}^{pr}_{zk_1}} \\
 & \\
\multicolumn{2}{c}{\mathbf{y}^{rp}_{zk_2}} \\
 & \\
 & \\
\hline
 & \\
\mathbf{y}^{rr} & \mathbf{y}^{rs} \\
\hline
 & \\
\mathbf{y}^{sr} & \mathbf{y}^{ss}
\end{array}\right]
\end{array}
\left[\begin{array}{c} t \\ t \\ t \\ t \\ \$ \\ \$ \\ \$ \\ \$ \\ \$ \end{array}\right]
\tag{1}
$$

$$
\mathbf{T}_{\text{hyb}} =
\begin{array}{lll}
 & & \\
 & & \\
 & & \\
 & k_1 & z \\
 & & h \\
p & k_2 & z \\
 & & h \\
 & a & c \\
r & & i \\
 & & c \\
s & & i \\
 & & c
\end{array}
\begin{array}{c}
\begin{array}{ccccccccc}
 & & p & & & r & & s & \\
k_1 & & k_2 & & a & & & & \\
z & h & z & h & i & i & c & i & c
\end{array} \\
\left[\begin{array}{ccccc|cc|cc}
 & & & & & & & & \\
\mathbf{T}^{k_1 z}_h & & & & & & & & \\
 & & & & & & & & \\
 & & \mathbf{T}^{k_2 z}_h & & & & & & \\
 & \dot{\mathbf{U}}^{k_1}_{ch} & & \dot{\mathbf{U}}^{k_2}_{ch} & & & & & \\
\hline
 & & & & & & \mathbf{V}^r & & \\
 & & & & \overset{*}{\mathbf{U}}^{rp}_{ci} & \bar{\mathbf{U}}^{rr} & & \mathbf{U}^{rs} & \\
\hline
 & & & & & & & & \mathbf{V}^s \\
 & & & & \overset{*}{\mathbf{U}}^{sp}_{ci} & \bar{\mathbf{U}}^{sr} & & \mathbf{U}^{ss} &
\end{array}\right]
\end{array}
\left[\begin{array}{c} t \\ t \\ t \\ t \\ \$ \\ \$ \\ \$ \\ \$ \\ \$ \end{array}\right]
\tag{2}
$$

SI-2.5 GLORIA country definitions

index	region acronym	region name	index	region acronym	region name
1	XAM	Rest of Americas	83	KAZ	Kazakhstan
2	XEU	Rest of Europe	84	KEN	Kenya
3	XAF	Rest of Africa	85	KGZ	Kyrgyzstan
4	XAS	Rest of Asia-Pacific	86	KHM	Cambodia
5	AFG	Afghanistan	87	KOR	South Korea
6	AGO	Angola	88	KWT	Kuwait
7	ALB	Albania	89	LAO	Laos
8	ARE	United Arab Emirates	90	LBN	Lebanon
9	ARG	Argentina	91	LBR	Liberia
10	ARM	Armenia	92	LBY	Libya
11	AUS	Australia	93	LKA	Sri Lanka
12	AUT	Austria	94	LTU	Lithuania
13	AZE	Azerbaijan	95	LUX	Luxembourg
14	BDI	Burundi	96	LVA	Latvia
15	BEL	Belgium	97	MAR	Morocco
16	BEN	Benin	98	MDA	Moldova
17	BFA	Burkina Faso	99	MDG	Madagascar
18	BGD	Bangladesh	100	MEX	Mexico
19	BGR	Bulgaria	101	MKD	Macedonia
20	BHR	Bahrain	102	MLI	Mali
21	BHS	Bahamas	103	MLT	Malta
22	BIH	Bosnia and Herzegovina	104	MMR	Myanmar
23	BLR	Belarus	105	MNG	Mongolia
24	BLZ	Belize	106	MOZ	Mozambique
25	BOL	Bolivia	107	MRT	Mauritania
26	BRA	Brazil	108	MWI	Malawi
27	BRN	Brunei Darussalam	109	MYS	Malaysia
28	BTN	Bhutan	110	NAM	Namibia
29	BWA	Botswana	111	NER	Niger
30	CAF	Central African Republic	112	NGA	Nigeria
31	CAN	Canada	113	NIC	Nicaragua
32	CHE	Switzerland	114	NLD	Netherlands
33	CHL	Chile	115	NOR	Norway
34	CHN	China	116	NPL	Nepal
35	CIV	Cote d'Ivoire	117	NZL	New Zealand
36	CMR	Cameroon	118	OMN	Oman
37	COD	DR Congo	119	PAK	Pakistan
38	COG	Rep Congo	120	PSE	Palestine
39	COL	Colombia	121	PAN	Panama
40	CRI	Costa Rica	122	PER	Peru
41	CUB	Cuba	123	PHL	Philippines
42	CYP	Cyprus	124	PNG	Papua New Guinea
43	CZE	CSSR/Czech Republic (1990/1991)	125	POL	Poland
44	DEU	Germany	126	PRK	North Korea
45	DJI	Djibouti	127	PRT	Portugal
46	DYE	DR Yemen (Aden)	128	PRY	Paraguay
47	DNK	Denmark	129	QAT	Qatar
48	DOM	Dominican Republic	130	ROU	Romania
49	DZA	Algeria	131	RUS	USSR/Russian Federation (1990/1991)
50	ECU	Ecuador	132	RWA	Rwanda
51	EGY	Egypt	133	SAU	Saudi Arabia
52	ERI	Eritrea	134	SDS	South Sudan
53	ESP	Spain	135	SEN	Senegal
54	EST	Estonia	136	SGP	Singapore
55	ETH	Ethiopia/DR Ethiopia (1992/1993)	137	SLE	Sierra Leone
56	FIN	Finland	138	SLV	El Salvador
57	FRA	France	139	SOM	Somalia
58	GAB	Gabon	140	SRB	Yugoslavia/Serbia (1991/1992)
59	GBR	United Kingdom	141	SDN	Sudan/North Sudan (2010/2011)
60	GEO	Georgia	142	SVK	Slovakia
61	GHA	Ghana	143	SVN	Slovenia
62	GIN	Guinea	144	SWE	Sweden
63	GMB	Gambia	145	SYR	Syria
64	GNQ	Equatorial Guinea	146	TCD	Chad
65	GRC	Greece	147	TGO	Togo
66	GTM	Guatemala	148	THA	Thailand
67	HND	Honduras	149	TJK	Tajikistan
68	HKG	Hong Kong	150	TKM	Turkmenistan
69	HRV	Croatia	151	TUN	Tunisia
70	HTI	Haiti	152	TUR	Turkey
71	HUN	Hungary	153	TZA	Tanzania
72	IDN	Indonesia	154	UGA	Uganda
73	IND	India	155	UKR	Ukraine
74	IRL	Ireland	156	URY	Uruguay
75	IRN	Iran	157	USA	United States of America
76	IRQ	Iraq	158	UZB	Uzbekistan
77	ISL	Iceland	159	VEN	Venezuela
78	ISR	Israel	160	VNM	Viet Nam
79	ITA	Italy	161	YEM	Yemen Arab Republic/Yemen (1990/1991)
80	JAM	Jamaica	162	ZAF	South Africa
81	JOR	Jordan	163	ZMB	Zambia
82	JPN	Japan	164	ZWE	Zimbabwe

SI-2.6 GLORIA sector definitions

index	sector name	index	sector name
1	Growing wheat	61	Printing
2	Growing maize	62	Coke oven products
3	Growing cereals n.e.c	63	Refined petroleum products
4	Growing leguminous crops and oil seeds	64	Nitrogenous fertilizers
5	Growing rice	65	Non-nitrogenous and mixed fertilizers
6	Growing vegetables, roots, tubers	66	Basic petrochemical products
7	Growing sugar beet and cane	67	Basic inorganic chemicals
8	Growing tobacco	68	Basic organic chemicals
9	Growing fibre crops	69	Pharmaceuticals and medicinal products
10	Growing crops n.e.c.	70	Dyes, paints, glues, detergents and other chemical products
11	Growing grapes	71	Rubber products
12	Growing fruits and nuts	72	Plastic products
13	Growing beverage crops (coffee, tea etc)	73	Clay building materials
14	Growing spices, aromatic, drug and pharmaceutical crops	74	Glass and other ceramics n.e.c.
15	Seeds and plant propagation	75	Cement, lime and plaster products
16	Raising of cattle	76	Other non-metallic mineral products n.e.c.
17	Raising of sheep and goats	77	Basic iron and steel
18	Raising of swine/pigs	78	Basic aluminium
19	Raising of poultry	79	Basic Copper
20	Raising of animals n.e.c.; services to agriculture	80	Basic Gold
21	Forestry and logging	81	Basic lead/zinc/silver
22	Fishing	82	Basic nickel
23	Crustaceans and molluscs	83	Basic tin
24	Hard coal	84	Basic non-ferrous metals n.e.c.
25	Lignite and peat	85	Fabricated metal products
26	Petroleum extraction	86	Machinery and equipment
27	Gas extraction	87	Motor vehicles, trailers and semi-trailers
28	Iron ores	88	Other transport equipment
29	Uranium ores	89	Repair and installation of machinery and equipment (service)
30	Aluminium ore	90	Computers; electronic products; optical and precision instruments
31	Copper ores	91	Electrical equipment
32	Gold ores	92	Furniture and other manufacturing n.e.c
33	Lead/zinc/silver ores	93	Electric power generation, transmission and distribution
34	Nickel ores	94	Distribution of gaseous fuels through mains
35	Tin ores	95	Water collection, treatment and supply; sewerage
36	Other non-ferrous ores	96	Waste collection, treatment, and disposal
37	Quarrying of stone, sand and clay	97	Materials recovery
38	Chemical and fertilizer minerals	98	Building construction
39	Extraction of salt	99	Civil engineering construction
40	Mining and quarrying n.e.c.; services to mining	100	Wholesale and retail trade; repair of motor vehicles and motorcycles
41	Beef meat	101	Road transport
42	Sheep meat	102	Rail transport
43	Pork	103	Transport via pipeline
44	Poultry meat	104	Water transport
45	Other meat products	105	Air transport
46	Fish products	106	Services to transport
47	Cereal products	107	Postal and courier services
48	Vegetable products	108	Hospitality
49	Fruit products	109	Publishing
50	Food products and feeds n.e.c.	110	Telecommunications
51	Sugar refining; cocoa, chocolate and confectionery	111	Information services
52	Animal oils and fats	112	Finance and insurance
53	Vegetable oils and fats	113	Property and real estate
54	Dairy products	114	Professional, scientific and technical services
55	Alcoholic and other beverages	115	Administrative services
56	Tobacco products	116	Government; social security; defence; public order
57	Textiles and clothing	117	Education
58	Leather and footwear	118	Human health and social work activities
59	Sawmill products	119	Arts, entertainment and recreation
60	Pulp and paper	120	Other services

SI-3 System boundary

Environmental impacts associated with passenger vehicles that are produced and then consumed in Japan are calculated in this study. Impacts arising from imported vehicles and vehicles produced for export are not considered here. Some vehicle types produced in negligible quantities are not considered, i.e. hydrogen fuel-cell and diesel vehicles. The assessment model accounts for upstream impacts, CO_2 emissions and material extraction, for vehicle production. Scope-3 emissions factors are used for fuel use (i.e. petrol) and electricity. Upstream impacts are also calculated for materials consumed during the vehicle use phase for maintenance and repairs (e.g. tyres, oil and batteries). Downstream impacts at vehicle end-of-life are not considered. Construction of infrastructure used by vehicle owners, such as roads and electric vehicle charging infrastructure is also outside the system boundary (though infrastructure is counted when it contributes to vehicle production). Figure 5 depicts of the system boundary used in this study. Use-phase impacts are counted for each year t in the vehicle lifetime, shown as a loop. Upstream impacts are shown as grey lines terminating in open circles.

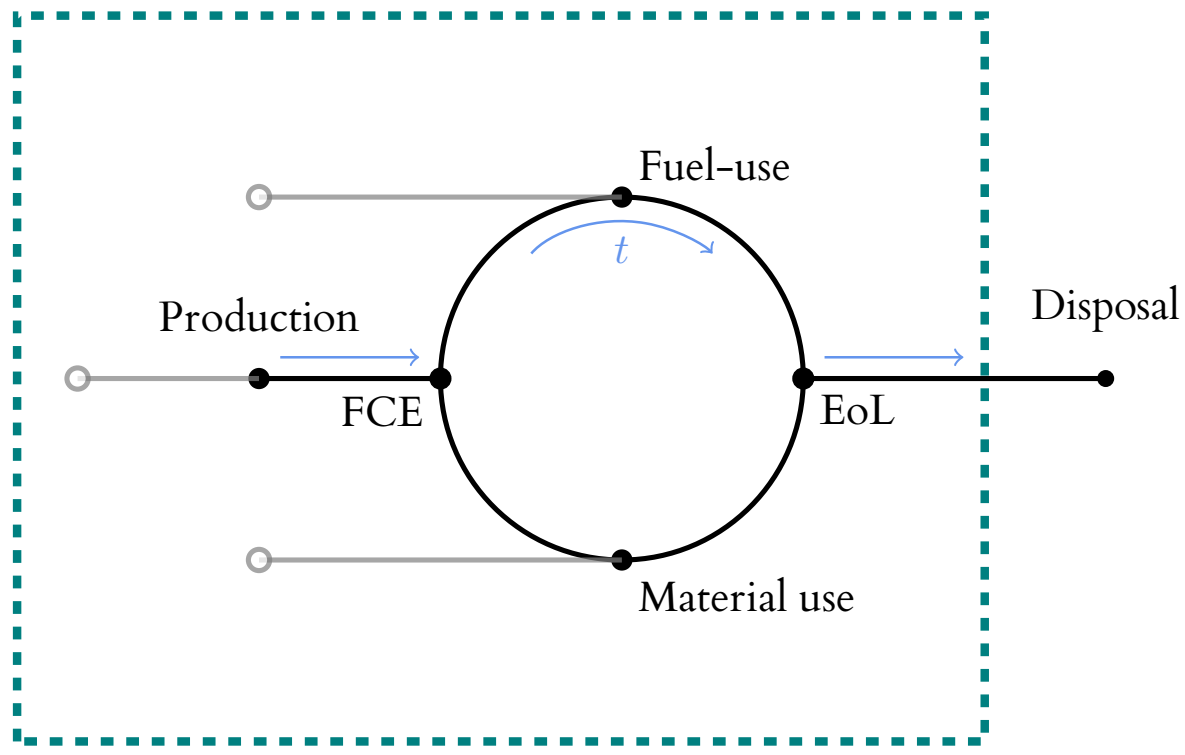

Figure 5: System boundary used in this study. The boundary is shown as the blue bounding box.

SI-4 Use-phase parameters

SI-4.1 Vehicle fuel economy

For ICV and HEV vehicles, the *e-nenpi* database of Japanese vehicles[1] was used to define relationships between vehicle weight and fuel efficiency. For PHEV vehicles, *e-nenpi* does not distinguish between petrol and electric motor efficiencies, therefore we used the WLTC catalog fuel economy from Toyota Motor Corporation[2]. The Toyota catalogue contains fuel efficiency for medium size (e.g. Prius) and large size (e.g. Crown, RAV4, Harrier, Alphard and Vellfire) PHEVs. For BEV vehicles we used European efficiencies (Weiss et al., 2024) and from Nissan[3] for small sized vehicles.

powertrain	size	value	units	source
ICE	small	18.5	km/L	e-nenpi
	medium	15.2	km/L	
	large	11.3	km/L	
HEV	small	30.2	km/L	e-nenpi
	medium	27.5	km/L	
	large	20.1	km/L	
PHEV	medium	26.0	km/L	Toyota
		6.6	km/kWh	
	large	19.2	km/L	
		5.0	km/kWh	
BEV	small	7.9	km/kWh	Weiss et al. (2024), Nissan
	medium	6.2	km/kWh	
	large	5.2	km/kWh	

Table 7: Vehicle efficiency parameters by vehicle type and size.

The *e-nenpi* database was used to construct a regression model of vehicle weight and efficiency, segmented by vehicle type. The model fit is shown in figures 6, 7 and 8 below. These data show the effect of vehicle kerb weight on fuel efficiency. However, we use these relationships to estimate the change in efficiency from an incremental change in vehicle weight i.e. from increasing the vehicle occupancy. We acknowledge this potential source of uncertainty in our approach.

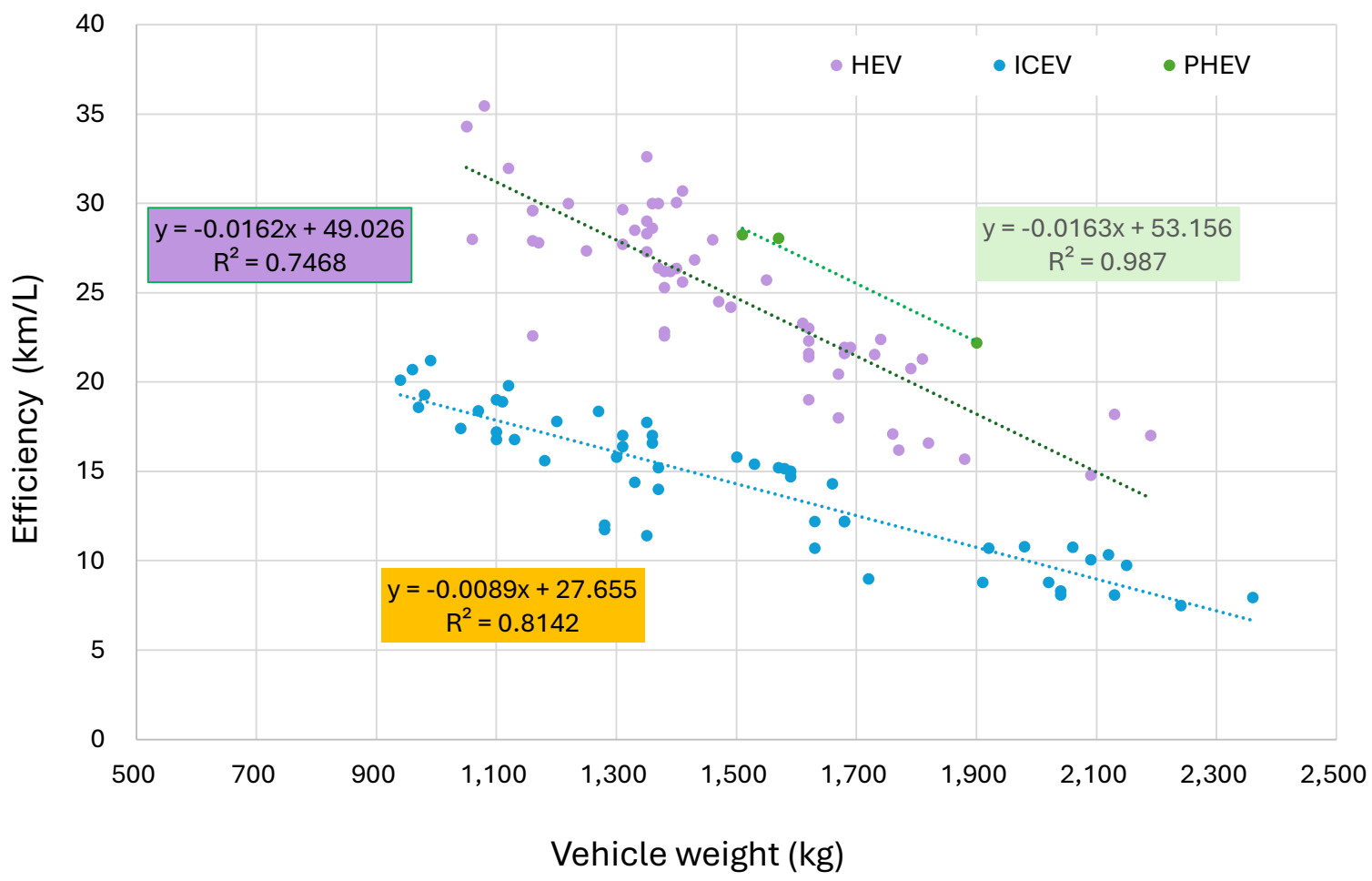

Figure 6: Fuel economy (km/L) and weight (kg) relationships for ICV, HEV and PHEV vehicles, from *e-nenpi*.

[1] `https://e-nenpi.com`
[2] `https://toyota.jp/info/e-toyota/phev/`
[3] `https://www3.nissan.co.jp/vehicles/new/sakura/specifications.html`

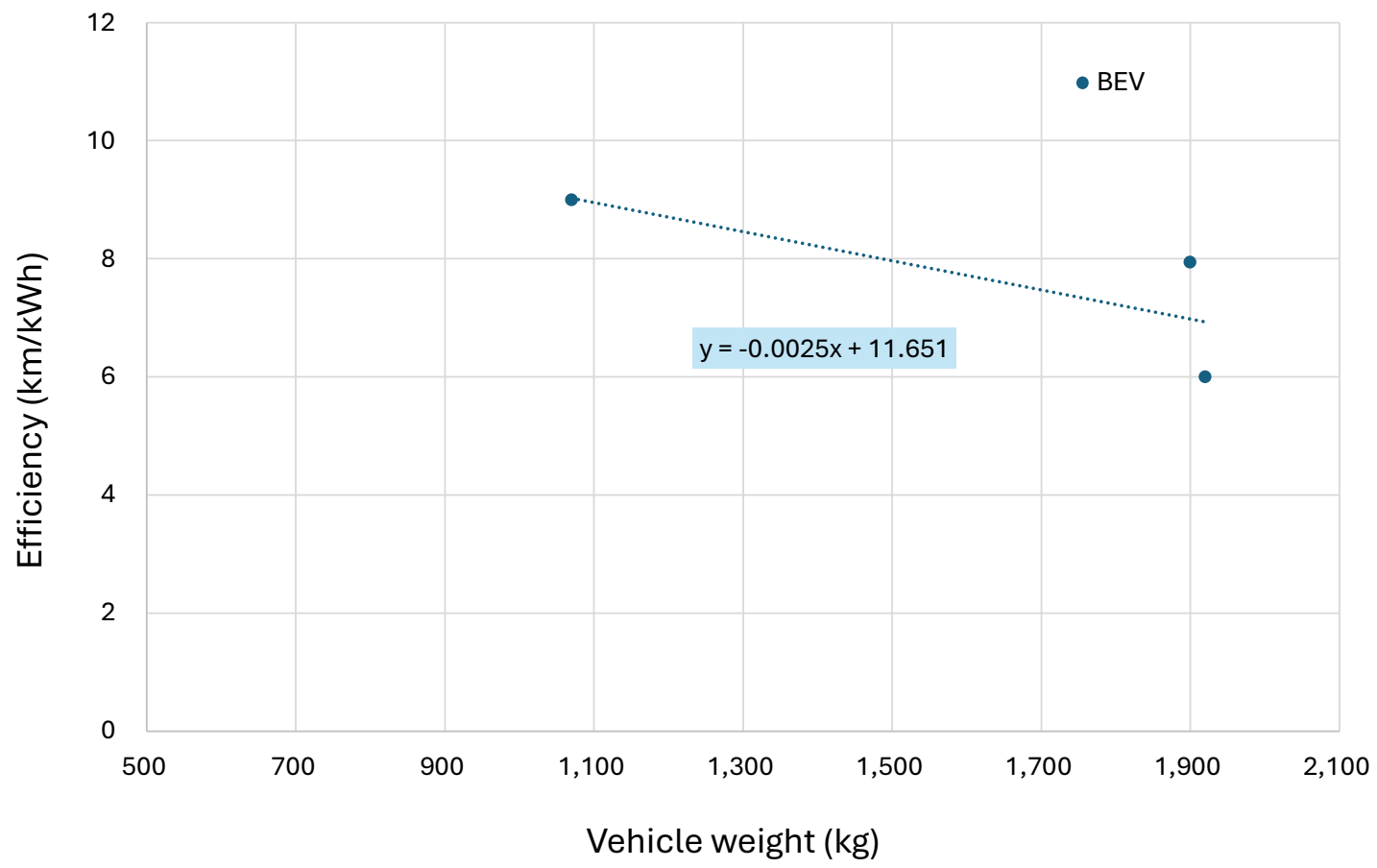

Figure 7: Fuel economy (km/kWh) and weight (kg) relationships for BEV vehicles from Nissan and Toyota.

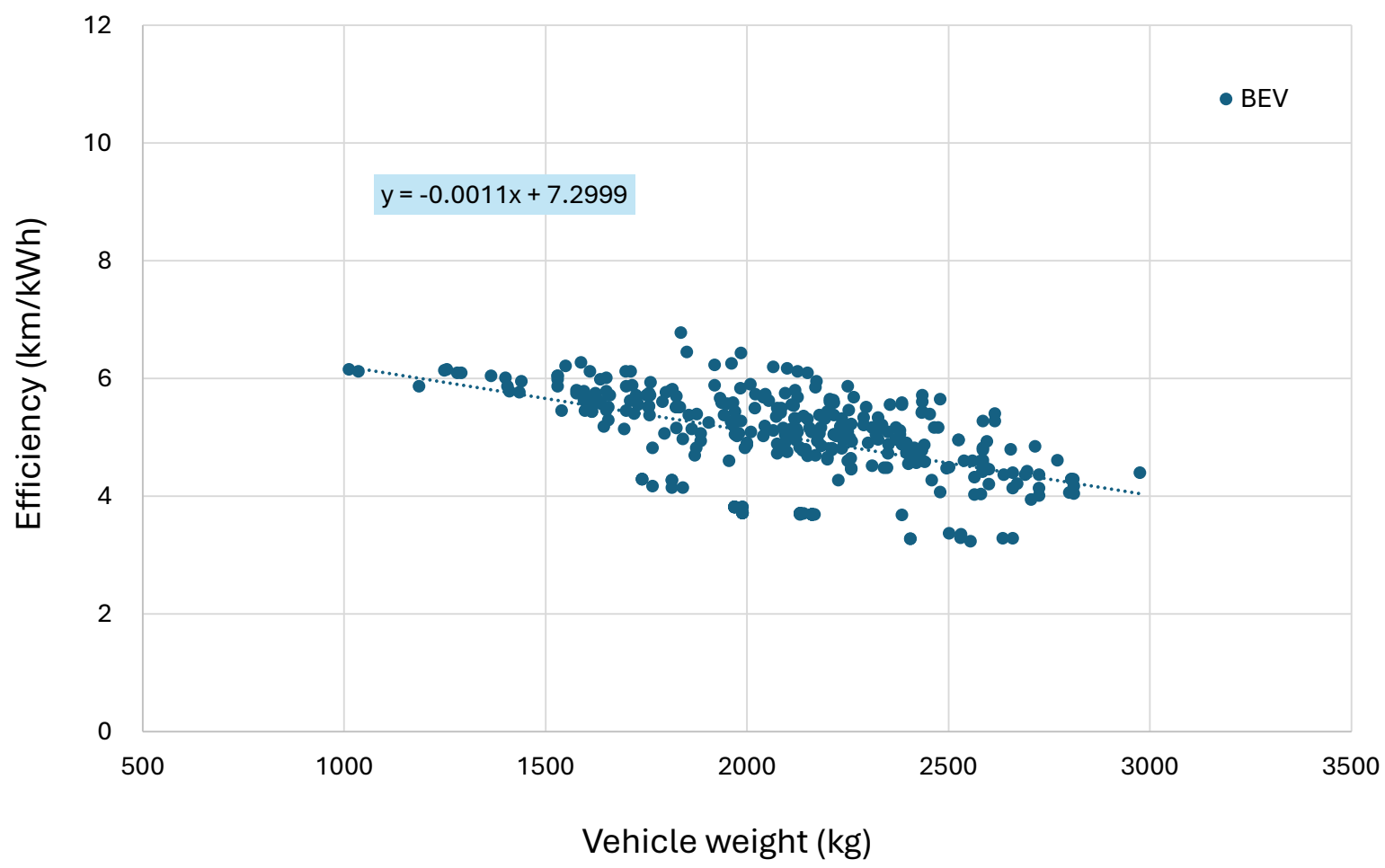

Figure 8: Fuel economy (km/kWh) and weight (kg) relationships for BEVs sold in Europe (Weiss et al., 2024).

SI-4.2 Other assumptions

Fuel emissions intensity (petrol) (DCCEEW, 2024):

- Energy density: 34.2 (GJ/kL)
- Emissions intensity: 0.2 + 67.8 + 17.2 = 85.2 (kg CO_2-e/GJ) (Scopes 1+2+3)
- Emissions intensity: 2.914 (kg CO2-e/L)
- PHEV use-phase emissions dimensions:
  $F_u = [\text{km}]\left([\text{kWh/km}][\text{kg CO}_2/\text{kWh}] + [\text{L/km}][\text{kg CO}_2/\text{L}]\right)$

Mobility service assumptions:

- Average vehicle occupancy Japan: 1.5 people/vehicle
- The vehicle catalogue weight includes one driver, with each additional passenger adding 60 kg.

SI-4.3 Grid electricity emissions intensity

Three scenarios of future grid emissions intensity are modelled in this study: IIEJ *Reference*, IEEJ *Advanced technologies* (IEEJ, 2024), as well as a 'No new policies' (static) scenario. The static scenario uses the *Reference* scenario until 2030, after which no further changes occur. For each electricity generation type, lifecycle emissions were estimated using NREL (2021) factors.

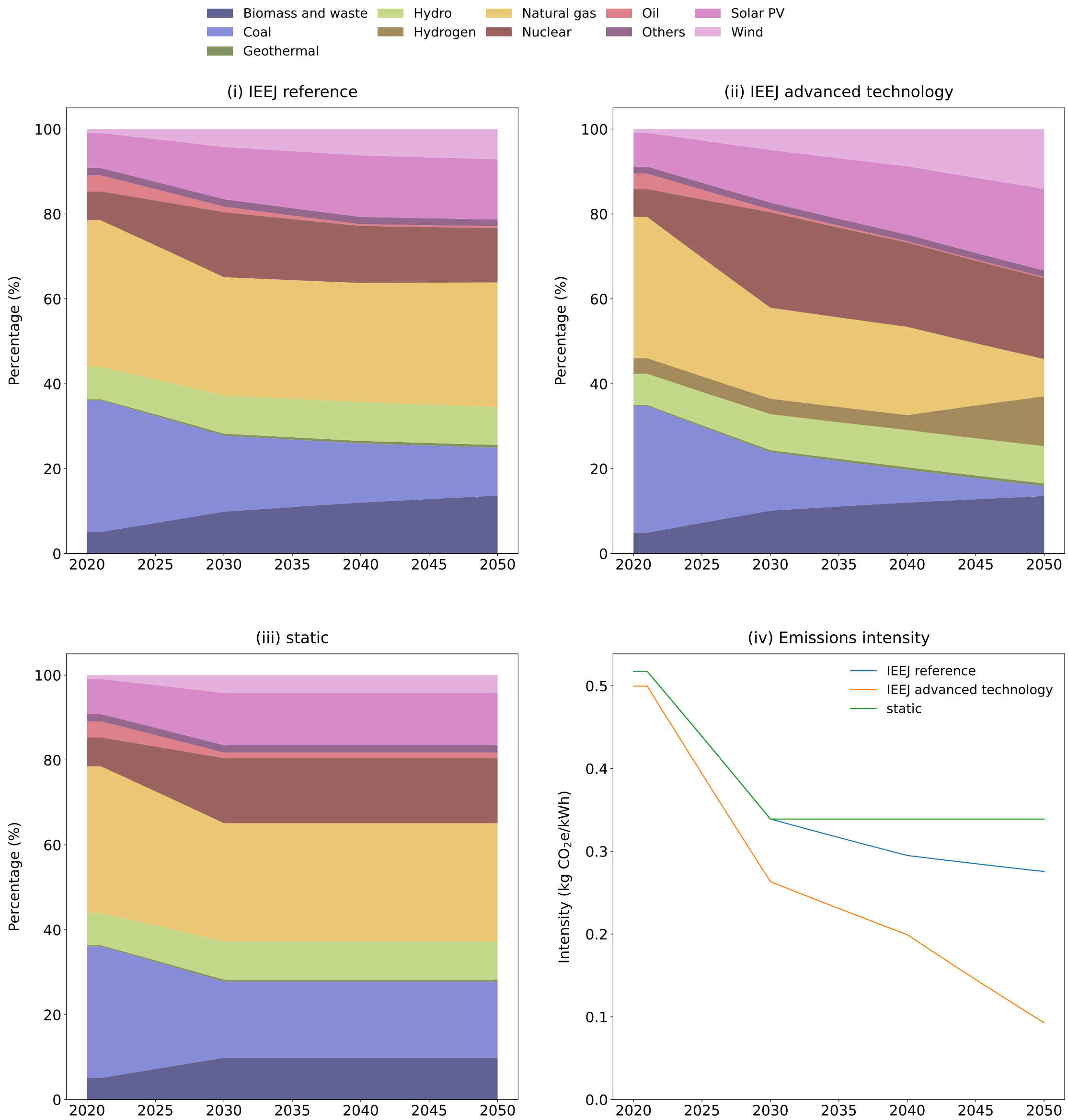

Figure 9: Forecasts of electricity grid generator mix and emissions intensity, by scenario.

SI-5 Mobility scenarios

SI-5.1 Mobility services and emissions

Let mobility services be defined:

$$M = \sum_{kz} \sigma d\, \mathbf{N}^{kz} \tag{3}$$

where σ is vehicle occupancy (p/vehicle), d is distance (km/vehicle), $\mathbf{N}^{kz}$ is counts of vehicles by powertrain k and size z (vehicles), and M has units passenger-km.

Mobility is related to lifecycle emissions through:

$$\acute{\mathbf{F}} = \mathbf{N}^{kz} \circ \mathbf{F}^{\rho} + \sum_t \mathbf{N}^{kz} \circ \mathbf{F}_t^u \tag{4}$$

$$\acute{F} = \sum_{kz} \acute{\mathbf{F}} \tag{5}$$

where $\mathbf{F}^{\rho}$ are production emissions and $\mathbf{F}^u$ are use-phase emissions in each year t.

Production emissions are a function of the counts of vehicles by powertrain and size:

$$F^{\rho} = \mathbf{qLy}^{kz} \tag{6}$$

where $\mathbf{y}_{kz}$ is constructed from the vehicle counts and material composition $\mathbf{G}$:

$$\mathbf{y}^{kz} = \mathbf{N}^{kz} \circ \sum_{mh} \mathbf{G}_{mh}^{kz} \tag{7}$$

$\mathbf{F}^u$ is also a function of the vehicle counts (since this influences the cohort fuel efficiency), distance travelled and passenger occupancy (since occupancy influences total weight):

$$F^u = (1/e)df \tag{8}$$

where e is the vehicle fuel efficiency (km/L or km/kWh) as a function of vehicle technology and size, d the distance driven per year (km), and f the fuel emissions intensity (kg CO_2/L or kg CO_2/kWh)

SI-5.2 Occupancy

One strategy is to increase the average vehicle occupancy, for example $\sigma_1 = 1.5 \rightarrow \sigma_2 = 2$. To model a change in vehicle occupancy, let's define the ratio:

$$\frac{M_1}{M_2} = \frac{\sigma_1 d_1 \mathbf{N}_1}{\sigma_2 d_2 \mathbf{N}_2} \tag{9}$$

Setting mobility services equal in both scenarios:

$$M_1 = M_2 \tag{10}$$

$$\frac{\sigma_2}{\sigma_1} = \frac{d_1 \mathbf{N}_1}{d_2 \mathbf{N}_2} \tag{11}$$

If the total number of vehicles is also constant, the yearly distance d increases by a factor of:

$$d_2 = \frac{d_1}{(\sigma_2/\sigma_1)} \tag{12}$$

SI-5.3 Downsizing

To model vehicle downsizing, a fraction of vehicles in the large category are shifted to the medium sized category. This decreases both production emissions, since smaller vehicles require less materials, and use phase emissions (lighter vehicles require less energy to move).

$$\mathbf{N}_2^{k,z=L} = \mathbf{N}_1^{k,z=L} - \delta \tag{13}$$
$$\mathbf{N}_2^{k,z=M} = \mathbf{N}_1^{k,z=M} + \delta \tag{14}$$

Sum over total vehicles is unchanged:

$$\sum_{kz} \mathbf{N}_1^{kz} = \sum_{kz} \mathbf{N}_2^{kz} \tag{15}$$

SI-5.4 Lifetime extension

Assume that mobility services delivered using 'standard' vehicle lifetimes is M_1 and delivered by extended vehicle lifetimes is M_2. Regardless of vehicle lifetime, we desire to total mobility services delivered to be unchanged, so that:

$$M_1 = M_2 \tag{16}$$
$$\sigma_1 d_1 \mathbf{N}_1 = \sigma_2 d_2 \mathbf{N}_2 \tag{17}$$

Here the vehicle lifetime must also be included. Let T represent the scenario vehicle lifetime and κ the number of years in the simulation. Then the equality becomes:

$$\frac{\kappa}{T_1}\sigma_1 d_1 \mathbf{N}_1 = \frac{\kappa}{T_2}\sigma_2 d_2 \mathbf{N}_2 \tag{18}$$

Extended vehicle lifetimes results in fewer vehicles in the total population. Rearranging for the updated vehicle counts:

$$\mathbf{N}_2 = \frac{T_2}{T_1}\mathbf{N}_1 \tag{19}$$

where occupancy and distance are both unchanged between the scenarios.

For standard vehicle lifetimes, total impacts for κ simulation steps can be written:

$$\acute{\mathbf{F}}_{T_1} = \frac{\kappa}{T}(\mathbf{N}_1 \circ \mathbf{F}^\rho) + \sum_t^\kappa \mathbf{N}_1 \circ \mathbf{F}^u \tag{20}$$

where $\mathbf{F}^u$ are use phase impacts. Now extending the vehicle lifetime from $T_1 \to T_2$:

$$\acute{\mathbf{F}}_{T_2} = \mathbf{N}_1 \circ \mathbf{F}^\rho + \left(\frac{\kappa}{T} - 1\right)\left[\mathbf{N}_2 \circ (\mathbf{F}^\rho + \mathbf{F}^\phi)\right] + \sum_t^\kappa \mathbf{N} \circ \mathbf{F}^u \tag{21}$$

where $\mathbf{N}_2$ is give by equation 19 and lifetime extension is possible with additional materials (i.e. for repair and maintenance) with associated impacts F^ϕ.

SI-5.5 Mobility segment and vehicle age cohorts

In this study we analyse one segment of the total mobility task through time. This means that while older vehicles leave the fleet, and newer vehicles join the fleet, the modelled cohort delivers a constant segment of the total mobility task. The modelled mobility strategies preserve delivered mobility, for example increasing occupancy requires less vehicles while delivered passenger-km are constant.

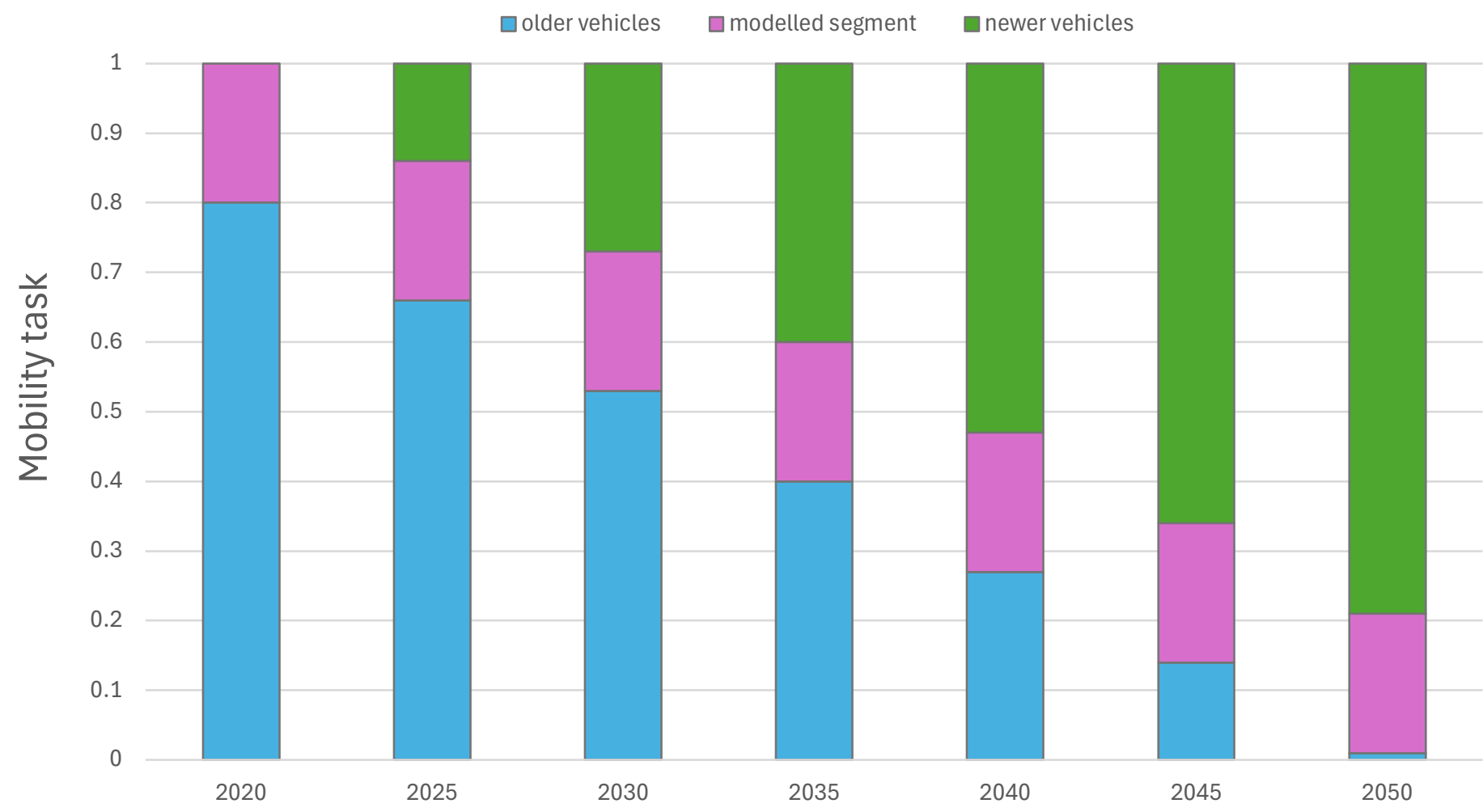

Figure 10: Following one segment of the mobility task while the larger vehicle fleet changes over time. Note segment sizes and timeseries are illustratitive.

SI-6 Path analysis

SI-6.1 IO decompositions

The Leontief inverse $\mathbf{L}$ can be represented by the power series expansion (Waugh, 1950):

$$\begin{aligned} F &= \mathbf{q}(\mathbf{I}-\mathbf{A})^{-1}\mathbf{y} \\ &= \mathbf{q}(\mathbf{I}+\mathbf{A}+\mathbf{A}^2+\mathbf{A}^3+\ldots)\mathbf{y} \\ &= \mathbf{qy}+\mathbf{qAy}+\mathbf{qA}^2\mathbf{y}+\mathbf{qA}^3\mathbf{y}+\ldots \end{aligned} \tag{22}$$

This can be further decomposed into the constituent supply chain paths using:

$$\begin{aligned} F &= \sum_{i,j=1}^{n} q_i(I-A)^{-1}_{ij}y_j \\ &= \sum_{i,j=1}^{n} q_i(\delta_{ij}+A_{ij}+A^2_{ij}+A^3_{ij}+\ldots)y_j \\ &= \sum_{i,j=1}^{n} q_i\left(\delta_{ij}+\sum_{k=1}^{n}A_{ik}A_{kj}+\sum_{l=1}^{n}\sum_{k=1}^{n}A_{il}A_{lk}A_{kj}+\ldots\right)y_j \end{aligned} \tag{23}$$

where δ_{ij} is the Kronecker delta and $\delta_{ij}=1$ if $i=j$ and zero otherwise. Indices (i,j,k,l) refer to industries and commodities, unique combinations of which reference specific paths. Taking the sum over all paths recovers the total footprint.

SI-6.2 Path substitution

Supply chains are edited under the following constraints:

(i) Swapping production inputs is only possible if the candidate trading partner produces a substitutable commodity. Subtle variations in quality and composition can determine whether a commodity is feasible to be used as a substitute production input. To account for this, a *substitutability* constraint is introduced to compare each exporter's production capacity with the importer's input requirements. This is done using a similarity metric to compare the trade vectors of the importer and candidate exporter, with editing performed if the similarity $\psi > 0.9$. See SI-6.3 for more detail.

(ii) While vehicle producer's have some ability to influence their supply chains, this influence likely decreases distant contributions to upstream suppliers. To model this, editing is permitted only if the second-last supply chain node is located in Japan.

(iii) Candidate trade partners are further filtered by total trade volume, to ensure these exporters have enough production capacity to meet the extra demand.

SI-6.3 Trade similarity

Swapping production inputs is only possible if the new trading partner produces a substitutable commodity. Therefore, *substitutability* is applied as an additional constraint to swapping by comparing each exporter's production capacity with the importer's input requirements. This comparison is performed at UN HS classification detail by defining each GLORIA sector in terms of HS in the binary concordance $\mathbf{C}_{j\nu}$, where each sector j is linked to a set of ν commodities. Export vectors $\mathbf{a}^{\theta}_{j\nu}$ and import vector $\mathbf{b}^{s}_{j\nu}$ can then be extracted from the BACI trade database (Gaulier and Zignago, 2022), for each GLORIA commodity j consisting of ν HS categories. Vectors $\mathbf{a}$ and $\mathbf{b}$ are reduced to binary representations, since the ability of region θ to produce commodity j is of interest, and not the magnitude of production. Similarity is measured by the set difference $\psi(\mathbf{a},\mathbf{b}) = 1-\frac{|\mathbf{a}-\mathbf{b}|}{|\mathbf{a}|}$ where $0 \leq \psi(\mathbf{a},\mathbf{b}) \leq 1$ and ψ approximates the substitutability of inputs. For alternate trade similarity metrics see the work of Finger and Kreinin (1979). Similarity between Japan import requirements and producer commodity exports is shown in Figure 11 below:

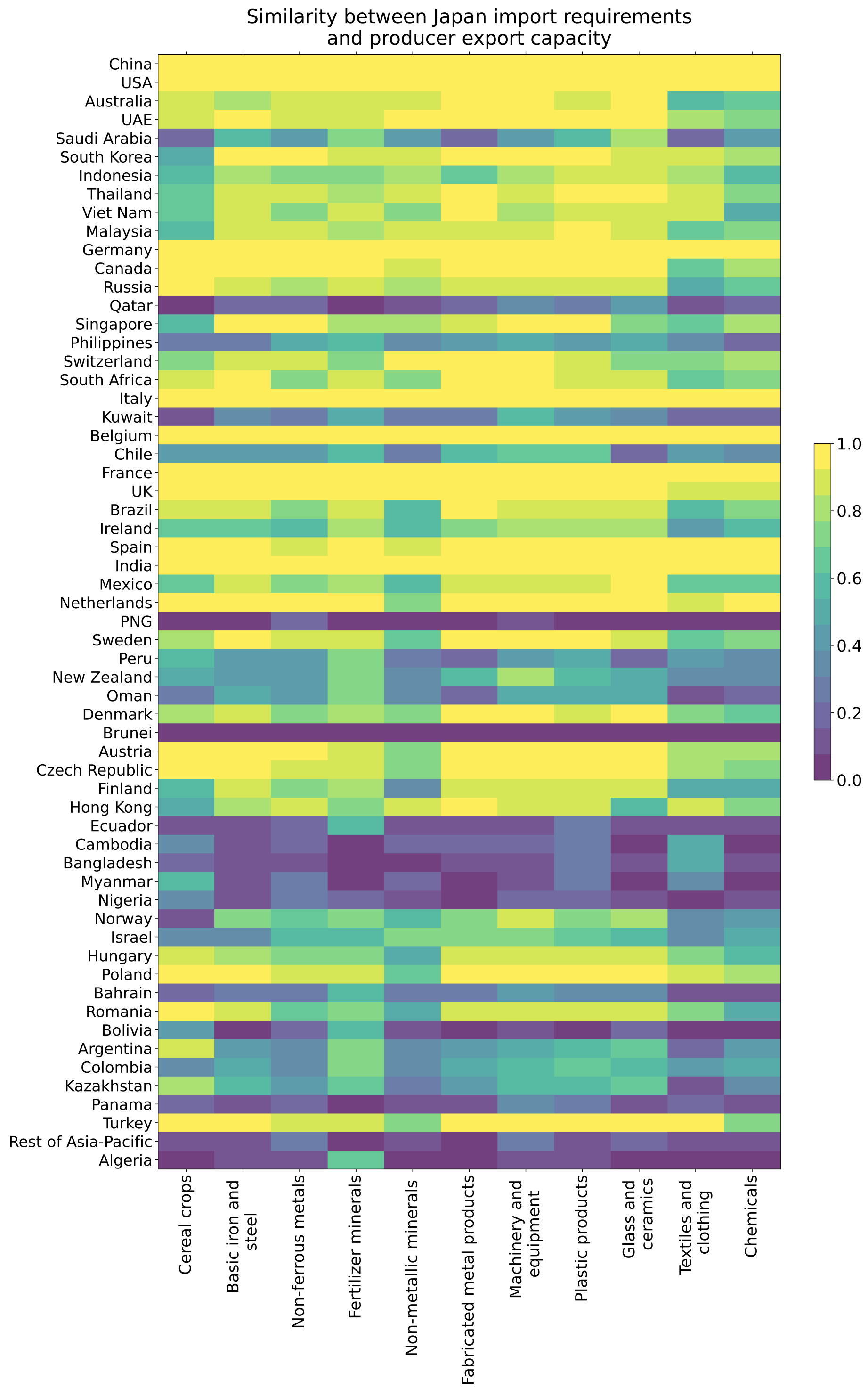

Figure 11: Similarity between Japan import requirements and producer export commodity mix, by region and selected commodities. Similarity of 1 indicates a better match between import requirements and export capacity.

SI-6.4 Embodied emissions and material structural paths

Table 8: Top 50 embodied emissions paths

rank	path	value kt CO_2e
1	ICE < Assembly < Electricity < Electricity (JPN)	676
2	ICE < Body < Hot rolled steel (JPN) < Crude steel (converters) (JPN) < Pig iron (JPN)	428
3	HEV < Assembly < Electricity < Electricity (JPN)	332
4	ICE < Chassis < Hot rolled steel (JPN) < Crude steel (converters) (JPN) < Pig iron (JPN)	249
5	ICE < Assembly < Freight transport < Freight transport (JPN)	249
6	ICE < Powertrain < Internal combustion engines (JPN) < Electricity (JPN)	182
7	ICE < Body < Cold-finished steel (JPN) < Hot rolled steel (JPN) < Crude steel (converters) (JPN) < Pig iron (JPN)	148
8	ICE < Assembly	144
9	ICE < Body < Sheet glass and safety glass < Sheet glass and safety glass (JPN)	143
10	HEV < Powertrain < Internal combustion engines (JPN) < Electricity (JPN)	137
11	HEV < Body < Hot rolled steel (JPN) < Crude steel (converters) (JPN) < Pig iron (JPN)	136
12	HEV < Assembly < Freight transport < Freight transport (JPN)	122
13	ICE < Powertrain < Internal combustion engines (JPN) < Hot rolled steel (JPN) < Crude steel (converters) (JPN) < Pig iron (JPN)	115
14	ICE < Transmission system < Hot rolled steel (JPN) < Crude steel (converters) (JPN) < Pig iron (JPN)	108
15	HEV < Powertrain < Internal combustion engines (JPN) < Hot rolled steel (JPN) < Crude steel (converters) (JPN) < Pig iron (JPN)	87
16	ICE < Transmission system < Motor vehicle parts (JPN) < Electricity (JPN)	86
17	ICE < Chassis < Cold-finished steel (JPN) < Hot rolled steel (JPN) < Crude steel (converters) (JPN) < Pig iron (JPN)	86
18	ICE < Powertrain < Internal combustion engines (JPN) < Cast and forged materials (iron) (JPN) < Pig iron (JPN)	84
19	ICE < Body < Coated steel (JPN) < Cold-finished steel (JPN) < Hot rolled steel (JPN) < Crude steel (converters) (JPN) < Pig iron (JPN)	79
20	ICE < Body < Plastic products (JPN) < Electricity (JPN)	75
21	HEV < Assembly	70
22	ICE < Powertrain < Hot rolled steel (JPN) < Crude steel (converters) (JPN) < Pig iron (JPN)	70
23	HEV < Battery cell < Hot rolled steel (JPN) < Crude steel (converters) (JPN) < Pig iron (JPN)	68
24	ICE < Assembly < Electricity (JPN) < Electricity (JPN)	65
25	HEV < Powertrain < Internal combustion engines (JPN) < Cast and forged materials (iron) (JPN) < Pig iron (JPN)	63
26	HEV < Chassis < Hot rolled steel (JPN) < Crude steel (converters) (JPN) < Pig iron (JPN)	58
27	ICE < Transmission system < Iron and steel shearing and slitting (JPN) < Hot rolled steel (JPN) < Crude steel (converters) (JPN) < Pig iron (JPN)	58
28	ICE < Body < Plastic products < Plastic products (JPN)	54
29	ICE < Transmission system < Iron and steel shearing and slitting < Iron and steel shearing and slitting (JPN)	52
30	ICE < Powertrain < Internal combustion engines < Internal combustion engines (JPN)	49
31	HEV < Body < Cold-finished steel (JPN) < Hot rolled steel (JPN) < Crude steel (converters) (JPN) < Pig iron (JPN)	47
32	ICE < Assembly < Coastal and inland water transport < Coastal and inland water transport (JPN)	47
33	ICE < Chassis < Coated steel (JPN) < Cold-finished steel (JPN) < Hot rolled steel (JPN) < Crude steel (converters) (JPN) < Pig iron (JPN)	46
34	HEV < Body < Sheet glass and safety glass < Sheet glass and safety glass (JPN)	45
35	HEV < Powertrain < Hot rolled steel (JPN) < Crude steel (converters) (JPN) < Pig iron (JPN)	43
36	ICE < Body < Hot rolled steel < Hot rolled steel (JPN)	43
37	ICE < Powertrain < Internal combustion engines (JPN) < Cast and forged materials (iron) (JPN) < Electricity (JPN)	43
38	ICE < Powertrain < Internal combustion engines (JPN) < Internal combustion engines (JPN) < Electricity (JPN)	41
39	ICE < Transmission system < Iron and steel shearing and slitting (JPN) < Cold-finished steel (JPN) < Hot rolled steel (JPN) < Crude steel (converters) (JPN) < Pig iron (JPN)	41
40	ICE < Assembly < Electricity (JPN) < Hard coal (IDN)	40
41	ICE < Transmission system < Motor vehicle parts (JPN) < Hot rolled steel (JPN) < Crude steel (converters) (JPN) < Pig iron (JPN)	40
42	ICE < Consumables < Motor vehicle parts (JPN) < Electricity (JPN)	40
43	HEV < Transmission system < Motor vehicle parts (JPN) < Electricity (JPN)	38
44	ICE < Transmission system < Cold-finished steel (JPN) < Hot rolled steel (JPN) < Crude steel (converters) (JPN) < Pig iron (JPN)	37
45	HEV < Powertrain < Internal combustion engines < Internal combustion engines (JPN)	37
46	HEV < Transmission system < Hot rolled steel (JPN) < Crude steel (converters) (JPN) < Pig iron (JPN)	37
47	ICE < Body < Sheet glass and safety glass (JPN) < Electricity (JPN)	37
48	ICE < Powertrain < Internal combustion engines (JPN) < Cast and forged materials (iron) (JPN)	34
49	ICE < Sub-battery < Motor vehicle parts (JPN) < Electricity (JPN)	34
50	HEV < Powertrain < Internal combustion engines (JPN) < Cast and forged materials (iron) (JPN) < Electricity (JPN)	32

Table 9: Top material extraction paths

rank	path	value<br>t ME
1	HEV-large < Powertrain (PRO) < Internal combustion engines (PRO) < Internal combustion engines (JPN) < Electric cables (JPN) < Copper (JPN) < Copper ores (CHL)	427,993
2	ICE-medium < Powertrain (PRO) < Internal combustion engines (PRO) < Internal combustion engines (JPN) < Electric cables (JPN) < Copper (JPN) < Copper ores (CHL)	376,435
3	ICE-small < Powertrain (PRO) < Internal combustion engines (PRO) < Internal combustion engines (JPN) < Electric cables (JPN) < Copper (JPN) < Copper ores (CHL)	333,524
4	ICE-medium < Body (PRO) < Electric cables (PRO) < Electric cables (JPN) < Copper (JPN) < Copper ores (CHL)	325,469
5	ICE-medium < Powertrain (PRO) < Electric cables (PRO) < Electric cables (JPN) < Copper (JPN) < Copper ores (CHL)	290,945
6	ICE-small < Body (PRO) < Electric cables (PRO) < Electric cables (JPN) < Copper (JPN) < Copper ores (CHL)	288,368
7	ICE-small < Powertrain (PRO) < Electric cables (PRO) < Electric cables (JPN) < Copper (JPN) < Copper ores (CHL)	257,779
8	ICE-large < Powertrain (PRO) < Internal combustion engines (PRO) < Internal combustion engines (JPN) < Electric cables (JPN) < Copper (JPN) < Copper ores (CHL)	239,306
9	ICE-large < Body (PRO) < Electric cables (PRO) < Electric cables (JPN) < Copper (JPN) < Copper ores (CHL)	206,906
10	ICE-large < Powertrain (PRO) < Electric cables (PRO) < Electric cables (JPN) < Copper (JPN) < Copper ores (CHL)	184,958
11	HEV-large < Body (PRO) < Electric cables (PRO) < Electric cables (JPN) < Copper (JPN) < Copper ores (CHL)	156,172
12	HEV-small < Powertrain (PRO) < Internal combustion engines (PRO) < Internal combustion engines (JPN) < Electric cables (JPN) < Copper (JPN) < Copper ores (CHL)	145,908
13	HEV-large < Battery cell (PRO) < Electric cables (PRO) < Electric cables (JPN) < Copper (JPN) < Copper ores (CHL)	144,188
14	HEV-medium < Powertrain (PRO) < Internal combustion engines (PRO) < Internal combustion engines (JPN) < Electric cables (JPN) < Copper (JPN) < Copper ores (CHL)	140,986
15	ICE-medium < Chassis (PRO) < Electric cables (PRO) < Electric cables (JPN) < Copper (JPN) < Copper ores (CHL)	128,660
16	ICE-small < Chassis (PRO) < Electric cables (PRO) < Electric cables (JPN) < Copper (JPN) < Copper ores (CHL)	113,993
17	ICE-medium < Body (PRO) < Nickel (PRO) < Basic nickel (IDN) < Nickel ores (IDN)	101,158
18	HEV-large < Powertrain (PRO) < Internal combustion engines (PRO) < Internal combustion engines (JPN) < Internal combustion engines (JPN) < Electric cables (JPN) < Copper (JPN) < Copper ores (CHL)	97,349
19	ICE-medium < Transmission system (PRO) < Motor vehicle parts (PRO) < Motor vehicle parts (JPN) < Electric cables (JPN) < Copper (JPN) < Copper ores (CHL)	92,072
20	HEV-large < Powertrain (PRO) < Electric cables (PRO) < Electric cables (JPN) < Copper (JPN) < Copper ores (CHL)	91,848
21	ICE-medium < Powertrain (PRO) < Nickel (PRO) < Basic nickel (IDN) < Nickel ores (IDN)	90,428
22	ICE-small < Body (PRO) < Nickel (PRO) < Basic nickel (IDN) < Nickel ores (IDN)	89,627
23	ICE-medium < Powertrain (PRO) < Internal combustion engines (PRO) < Internal combustion engines (JPN) < Internal combustion engines (JPN) < Electric cables (JPN) < Copper (JPN) < Copper ores (CHL)	85,622
24	ICE-large < Chassis (PRO) < Electric cables (PRO) < Electric cables (JPN) < Copper (JPN) < Copper ores (CHL)	81,791
25	ICE-small < Transmission system (PRO) < Motor vehicle parts (PRO) < Motor vehicle parts (JPN) < Electric cables (JPN) < Copper (JPN) < Copper ores (CHL)	81,577
26	ICE-small < Powertrain (PRO) < Nickel (PRO) < Basic nickel (IDN) < Nickel ores (IDN)	80,120
27	BEV-large < Battery cell (PRO) < Electric cables (PRO) < Electric cables (JPN) < Copper (JPN) < Copper ores (CHL)	77,089
28	ICE-small < Powertrain (PRO) < Internal combustion engines (PRO) < Internal combustion engines (JPN) < Internal combustion engines (JPN) < Electric cables (JPN) < Copper (JPN) < Copper ores (CHL)	75,862
29	HEV-large < Powertrain (PRO) < Internal combustion engines (PRO) < Internal combustion engines (JPN) < Rolled and drawn copper and copper alloys (JPN) < Copper (JPN) < Copper ores (CHL)	70,984
30	HEV-large < Transmission system (PRO) < Rolled and drawn copper and copper alloys (PRO) < Rolled and drawn copper and copper alloys (JPN) < Copper (JPN) < Copper ores (CHL)	69,897
31	ICE-medium < Transmission system (PRO) < Aluminum (PRO) < Basic aluminium (AUS) < Quarrying of stone, sand and clay (BRA)	68,055
32	ICE-medium < Assembly (PRO) < Electricity (PRO) < Electricity (JPN) < Hard coal (IDN)	66,690
33	ICE-large < Body (PRO) < Nickel (PRO) < Basic nickel (IDN) < Nickel ores (IDN)	64,308
34	ICE-medium < Powertrain (PRO) < Internal combustion engines (PRO) < Internal combustion engines (JPN) < Rolled and drawn copper and copper alloys (JPN) < Copper (JPN) < Copper ores (CHL)	62,433
35	HEV-large < Transmission system (PRO) < Motor vehicle parts (PRO) < Motor vehicle parts (JPN) < Electric cables (JPN) < Copper (JPN) < Copper ores (CHL)	60,621
36	ICE-small < Assembly (PRO) < Electricity (PRO) < Electricity (JPN) < Hard coal (IDN)	60,599
37	ICE-small < Transmission system (PRO) < Aluminum (PRO) < Basic aluminium (AUS) < Quarrying of stone, sand and clay (BRA)	60,298
38	HEV-medium < Battery cell (PRO) < Electric cables (PRO) < Electric cables (JPN) < Copper (JPN) < Copper ores (CHL)	60,139
39	ICE-large < Transmission system (PRO) < Motor vehicle parts (PRO) < Motor vehicle parts (JPN) < Electric cables (JPN) < Copper (JPN) < Copper ores (CHL)	58,532
40	ICE-large < Powertrain (PRO) < Nickel (PRO) < Basic nickel (IDN) < Nickel ores (IDN)	57,487
41	ICE-medium < Body (PRO) < Sheet glass and safety glass (PRO) < Sheet glass and safety glass (JPN) < Quarrying of stone, sand and clay (USA)	56,203
42	ICE-small < Powertrain (PRO) < Internal combustion engines (PRO) < Internal combustion engines (JPN) < Rolled and drawn copper and copper alloys (JPN) < Copper (JPN) < Copper ores (CHL)	55,316
43	ICE-large < Powertrain (PRO) < Internal combustion engines (PRO) < Internal combustion engines (JPN) < Internal combustion engines (JPN) < Electric cables (JPN) < Copper (JPN) < Copper ores (CHL)	54,431
44	HEV-small < Body (PRO) < Electric cables (PRO) < Electric cables (JPN) < Copper (JPN) < Copper ores (CHL)	53,241
45	HEV-medium < Body (PRO) < Electric cables (PRO) < Electric cables (JPN) < Copper (JPN) < Copper ores (CHL)	51,445
46	HEV-large < Powertrain (PRO) < Internal combustion engines (PRO) < Internal combustion engines (JPN) < Electric cables (JPN) < Copper (JPN) < Copper ores (PAN)	51,428
47	ICE-small < Body (PRO) < Sheet glass and safety glass (PRO) < Sheet glass and safety glass (JPN) < Quarrying of stone, sand and clay (USA)	49,796
48	ICE-small < Consumables (PRO) < Motor vehicle parts (PRO) < Motor vehicle parts (JPN) < Electric cables (JPN) < Copper (JPN) < Copper ores (CHL)	49,178
49	HEV-large < Assembly (PRO) < Electricity (PRO) < Electricity (JPN) < Hard coal (IDN)	49,175
50	ICE-medium < Body (PRO) < Hot rolled steel (PRO) < Hot rolled steel (JPN) < Crude steel (converters) (JPN) < Pig iron (JPN) < Coal products (JPN) < Hard coal (IDN)	48,892

Table 10: Path emissions intensity and reduction potential. Path intensity shown as emissions per vehicle (kg CO_2e/vehicle). Reduction potential shown as both emissions per vehicle (kg CO_2e/vehicle) and as a percentage (%) of the total vehicle footprint. PRO: process model.

rank	path	trade partner substitution	reduction (kt CO_2e)	footprint (Δ%)
1	BEV < Battery cell (PRO) < Aluminum (PRO) < Basic aluminium (CAN)	CHN -> CAN	1.42	0.91
2	BEV < Battery cell (PRO) < Basic Copper (IDN)	CHN -> IDN	0.40	0.25
3	BEV < Battery cell (PRO) < Aluminum (PRO) < Basic aluminium (AUS)	RUS -> AUS	0.35	0.22
4	PHEV < Battery cell (PRO) < Aluminum (PRO) < Basic aluminium (CAN)	CHN -> CAN	0.33	0.18
5	BEV < Battery cell (PRO) < Aluminum (PRO) < Basic aluminium (BHR)	AUS -> BHR	0.18	0.11
6	BEV < Battery cell (PRO) < Aluminum (PRO) < Basic aluminium (BHR)	ARE -> BHR	0.16	0.10
7	BEV < Battery cell (PRO) < Miscellaneous non-ferrous metals (PRO) < Basic non-ferrous metals n.e.c. (CHN)	RUS -> CHN	0.15	0.10
8	BEV < Battery cell (PRO) < Aluminum (PRO) < Basic aluminium (AUS)	BRA -> AUS	0.15	0.09
9	BEV < Battery cell (PRO) < Miscellaneous non-ferrous metals (PRO) < Basic non-ferrous metals n.e.c. (ZAF)	USA -> ZAF	0.13	0.08
10	BEV < Battery cell (PRO) < Basic Copper (ISR)	THA -> ISR	0.09	0.06
11	BEV < Battery cell (PRO) < Miscellaneous non-ferrous metals (PRO) < Basic non-ferrous metals n.e.c. (DEU)	ZAF -> DEU	0.09	0.06
12	BEV < Battery cell (PRO) < Miscellaneous industrial organic chemicals (PRO) < Basic organic chemicals (USA)	CHN -> USA	0.08	0.05
13	ICE < Transmission system (PRO) < Aluminum (PRO) < Basic aluminium (CAN)	CHN -> CAN	13.03	0.08
14	PHEV < Battery cell (PRO) < Aluminum (PRO) < Basic aluminium (AUS)	RUS -> AUS	0.08	0.05
15	PHEV < Battery cell (PRO) < Basic Copper (IDN)	CHN -> IDN	0.08	0.04
16	PHEV < Powertrain (PRO) < Internal combustion engines (PRO) < Motor vehicles, trailers and semi-trailers (BIH)	VNM -> BIH	0.07	0.04
17	HEV < Powertrain (PRO) < Internal combustion engines (PRO) < Motor vehicles, trailers and semi-trailers (BIH)	VNM -> BIH	4.05	0.05
18	ICE < Chassis (PRO) < Aluminum (PRO) < Basic aluminium (CAN)	CHN -> CAN	9.02	0.06
19	BEV < Battery cell (PRO) < Miscellaneous industrial inorganic chemicals (PRO) < Basic inorganic chemicals (CAN)	CHN -> CAN	0.05	0.03
20	BEV < Battery cell (PRO) < Miscellaneous non-ferrous metals (PRO) < Basic non-ferrous metals n.e.c. (BEL)	CHN -> BEL	0.05	0.03
21	BEV < Battery cell (PRO) < Aluminum (PRO) < Basic aluminium (BHR)	KOR -> BHR	0.05	0.03
22	BEV < Battery cell (PRO) < Basic Copper (POL)	KOR -> POL	0.04	0.03
23	BEV < Battery cell (PRO) < Lead and zinc (including regenerated lead) (PRO) < Basic lead/zinc/silver (BGR)	KOR -> BGR	0.04	0.03
24	HEV < Battery cell (PRO) < Aluminum (PRO) < Basic aluminium (CAN)	CHN -> CAN	2.95	0.04
25	BEV < Battery cell (PRO) < Aluminum (PRO) < Basic aluminium (SVN)	USA -> SVN	0.04	0.02
26	HEV < Powertrain (PRO) < Internal combustion engines (PRO) < Motor vehicles, trailers and semi-trailers (IDN)	THA -> IDN	2.83	0.03
27	HEV < Powertrain (PRO) < Internal combustion engines (JPN) < Basic aluminium (CAN)	CHN -> CAN	2.56	0.03
28	BEV < Battery cell (PRO) < Nickel (PRO) < Basic nickel (FIN)	CAN -> FIN	0.03	0.02
29	ICE < Powertrain (PRO) < Aluminum (PRO) < Basic aluminium (CAN)	CHN -> CAN	5.39	0.03
30	ICE < Powertrain (PRO) < Internal combustion engines (PRO) < Motor vehicles, trailers and semi-trailers (BIH)	VNM -> BIH	5.38	0.03
31	HEV < Chassis (PRO) < Aluminum (PRO) < Basic aluminium (CAN)	CHN -> CAN	2.09	0.03
32	PHEV < Powertrain (PRO) < Internal combustion engines (PRO) < Motor vehicles, trailers and semi-trailers (HUN)	CHN -> HUN	0.03	0.02
33	ICE < Body (PRO) < Sheet glass and safety glass (JPN) < Other non-metallic mineral products n.e.c. (ZAF)	CHN -> ZAF	4.21	0.03
34	PHEV < Powertrain (PRO) < Internal combustion engines (JPN) < Motor vehicles, trailers and semi-trailers (BIH)	VNM -> BIH	0.03	0.02
35	ICE < Transmission system (PRO) < Aluminum (PRO) < Basic aluminium (AUS)	RUS -> AUS	4.09	0.03
36	HEV < Powertrain (PRO) < Internal combustion engines (JPN) < Motor vehicles, trailers and semi-trailers (BIH)	VNM -> BIH	1.70	0.02
37	ICE < Powertrain (PRO) < Internal combustion engines (PRO) < Motor vehicles, trailers and semi-trailers (IDN)	THA -> IDN	3.76	0.02
38	HEV < Powertrain (PRO) < Aluminum (PRO) < Basic aluminium (CAN)	CHN -> CAN	1.55	0.02
39	HEV < Transmission system (PRO) < Aluminum (PRO) < Basic aluminium (CAN)	CHN -> CAN	1.47	0.02
40	ICE < Body (PRO) < Basic Copper (IDN)	CHN -> IDN	3.41	0.02
41	ICE < Powertrain (PRO) < Internal combustion engines (JPN) < Basic aluminium (CAN)	CHN -> CAN	3.40	0.02
42	BEV < Battery cell (PRO) < Basic Copper (PRT)	MYS -> PRT	0.02	0.01
43	HEV < Powertrain (PRO) < Internal combustion engines (PRO) < Motor vehicles, trailers and semi-trailers (HUN)	CHN -> HUN	1.40	0.02
44	ICE < Body (PRO) < Plastic products (JPN) < Basic organic chemicals (USA)	CHN -> USA	3.20	0.02
45	HEV < Body (PRO) < Sheet glass and safety glass (JPN) < Other non-metallic mineral products n.e.c. (ZAF)	CHN -> ZAF	1.34	0.02
46	ICE < Powertrain (PRO) < Basic Copper (IDN)	CHN -> IDN	3.05	0.02
47	PHEV < Powertrain (PRO) < Internal combustion engines (JPN) < Motor vehicles, trailers and semi-trailers (IDN)	THA -> IDN	0.02	0.01
48	ICE < Chassis (PRO) < Aluminum (PRO) < Basic aluminium (AUS)	RUS -> AUS	2.83	0.02
49	HEV < Powertrain (PRO) < Internal combustion engines (JPN) < Motor vehicles, trailers and semi-trailers (IDN)	THA -> IDN	1.19	0.01
50	HEV < Powertrain (PRO) < Internal combustion engines (JPN) < Non-ferrous metal castings and forgings (JPN) < Aluminum (JPN) < Basic aluminium (AUS)	RUS -> AUS	1.09	0.01

SI-7 Extended results

SI-7.1 Material footprints

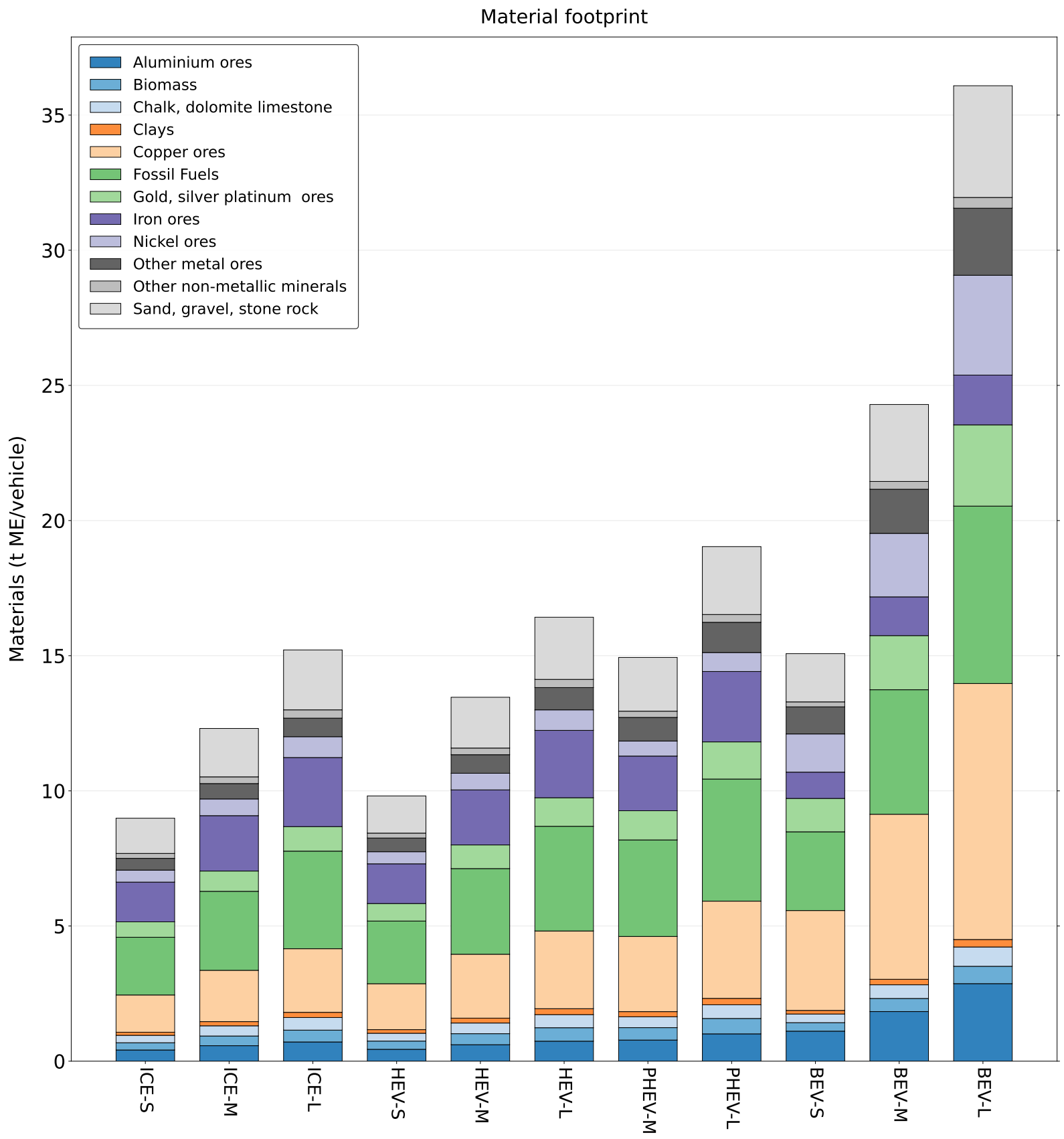

Figure 12: Material extraction footprints by vehicle size and technology.

SI-7.2 Lifecycle emissions

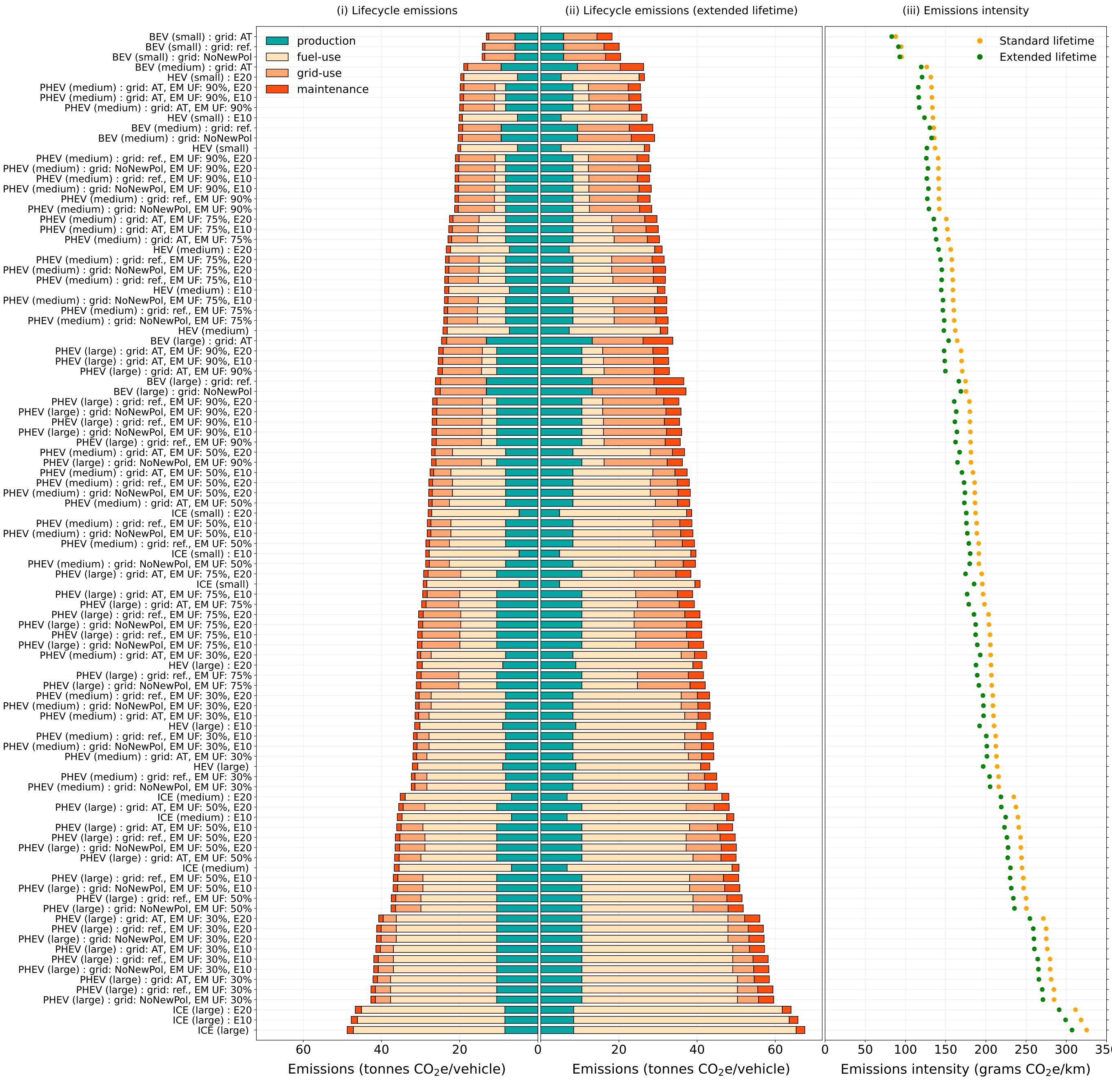

Figure 13: Ranked production and use-phase emissions by vehicle technology and use-phase scenario.

SI-7.3 Production Layer Decompositions

Table 11: Emissions production layer decomposition by commodity (kilotonnes)

commodity	layer 1	2	3	4	5	6	7	8	9
Growing wheat	0.01	0	0	0	0	0	0	0	0
Growing maize	0.01	0	0	0	0	0	0	0	0
Growing cereals n.e.c	0.04	0	0	0	0	0	0	0	0
Growing leguminous crops and oil seeds	0.1	0.01	0	0	0	0	0	0	0
Growing rice	0	0	0	0	0	0	0	0	0
Growing vegetables, roots, tubers	0.01	0	0	0	0	0	0	0	0
Growing sugar beet and cane	0	0	0	0	0	0	0	0	0
Growing tobacco	0.07	0	0	0	0	0	0	0	0
Growing fibre crops	0.01	0	0	0	0	0	0	0	0
Growing crops n.e.c.	0	0	0	0	0	0	0	0	0
Growing grapes	0.01	0	0	0	0	0	0	0	0
Growing fruits and nuts	0.04	0.01	0	0	0	0	0	0	0
Growing beverage crops (coffee, tea etc)	0.07	0	0	0	0	0	0	0	0
Growing spices, aromatic, drug and pharmaceutical crops	0.06	0	0	0	0	0	0	0	0
Seeds and plant propagation	0.06	0.01	0	0	0	0	0	0	0
Raising of cattle	0	0	0	0	0	0	0	0	0
Raising of sheep and goats	0.42	0.18	0.08	0.04	0.02	0.01	0.01	0	0
Raising of swine/pigs	0.09	0.01	0.01	0	0	0	0	0	0
Raising of poultry	0	0	0	0	0	0	0	0	0
Raising of animals n.e.c.; services to agriculture	0.07	0	0	0	0	0	0	0	0
Forestry and logging	0.02	0	0	0	0	0	0	0	0
Fishing	0	0.03	0.01	0.01	0	0	0	0	0
Crustaceans and molluscs	0.02	0.01	0	0	0	0	0	0	0
Hard coal	0.06	0.18	0.05	0.03	0.02	0.01	0.01	0	0
Lignite and peat	0.55	0.2	0.05	0.03	0.02	0.01	0.01	0	0
Petroleum extraction	0.4	0.44	0.18	0.11	0.06	0.03	0.02	0.01	0.01
Gas extraction	0.02	0.18	0.04	0.03	0.02	0.01	0.01	0	0
Iron ores	1.56	1.02	0.39	0.21	0.12	0.07	0.04	0.02	0.01
Uranium ores	0.37	0.19	0.03	0.01	0.01	0	0	0	0
Aluminium ore	0.05	0.08	0.03	0.02	0.01	0	0	0	0
Copper ores	1.51	0.25	0.11	0.06	0.03	0.02	0.01	0.01	0
Gold ores	0.67	0.91	0.88	0.59	0.36	0.21	0.12	0.07	0.04
Lead/zinc/silver ores	1.55	0.06	0.02	0.01	0	0	0	0	0
Nickel ores	1.07	1.31	0.85	0.37	0.24	0.14	0.08	0.05	0.03
Tin ores	0.03	0.01	0	0	0	0	0	0	0
Other non-ferrous ores	5.66	0.57	0.22	0.12	0.06	0.03	0.02	0.01	0.01
Quarrying of stone, sand and clay	1.5	0.31	0.14	0.08	0.05	0.03	0.02	0.01	0.01
Chemical and fertilizer minerals	0.92	0.06	0.03	0.02	0.01	0.01	0	0	0
Extraction of salt	1.2	0.22	0.1	0.05	0.02	0.01	0.01	0	0
Mining and quarrying n.e.c.; services to mining	0.04	0.02	0.01	0	0	0	0	0	0
Beef meat	0	0.01	0	0	0	0	0	0	0
Sheep meat	0.01	0	0.01	0	0	0	0	0	0
Pork	0	0	0	0	0	0	0	0	0
Poultry meat	0	0	0	0	0	0	0	0	0
Other meat products	0.01	0	0	0	0	0	0	0	0
Fish products	0.01	0	0	0	0	0	0	0	0
Cereal products	0	0	0	0	0	0	0	0	0
Vegetable products	0	0	0	0	0	0	0	0	0
Fruit products	0	0	0	0	0	0	0	0	0
Food products and feeds n.e.c.	0.01	0.01	0.01	0	0	0	0	0	0
Sugar refining; cocoa, chocolate and confectionery	0.04	0.18	0.04	0.02	0.01	0.01	0	0	0
Animal oils and fats	0.15	1.59	0.13	0.06	0.03	0.01	0.01	0	0
Vegetable oils and fats	0.26	0.5	0.12	0.06	0.03	0.02	0.01	0.01	0
Dairy products	0.11	0.82	0.29	0.14	0.07	0.04	0.02	0.01	0.01
Alcoholic and other beverages	0.03	0.18	0.07	0.04	0.03	0.02	0.01	0.01	0
Tobacco products	0.81	1.8	1.74	1.4	1.02	0.69	0.44	0.28	0.18
Textiles and clothing	4	36.8	18.59	11.75	7.34	4.58	2.88	1.79	1.1
Leather and footwear	0.59	2.35	1.2	0.71	0.42	0.25	0.15	0.09	0.05
Sawmill products	0.38	1.65	1.4	1.03	0.87	0.5	0.26	0.14	0.07
Pulp and paper	0.73	0.65	0.45	0.24	0.13	0.07	0.04	0.02	0.01
Printing	0.96	3.01	2.51	1.26	0.7	0.4	0.23	0.13	0.07
Coke oven products	0.04	0	0	0	0	0	0	0	0
Refined petroleum products	2.68	1.74	1.85	0.75	0.4	0.21	0.11	0.06	0.04
Nitrogenous fertilizers	0.03	0.07	0.05	0.02	0.01	0.01	0	0	0
Non-nitrogenous and mixed fertilizers	4.16	3.71	1.7	0.83	0.42	0.22	0.12	0.07	0.04

Basic petrochemical products	4.94	4.92	2.63	1.34	0.68	0.36	0.2	0.11	0.06
Basic inorganic chemicals	3.12	2.2	1.09	0.5	0.26	0.14	0.08	0.04	0.02
Basic organic chemicals	6.18	3.25	2.31	1.26	0.67	0.37	0.21	0.12	0.07
Pharmaceuticals and medicinal products	8.57	11.52	9.6	6.43	3.99	2.33	1.34	0.77	0.45
Dyes, paints, glues, detergents and other chemical products	16.26	42.06	35.12	21.51	12.29	7.02	3.96	2.17	1.21
Rubber products	23.52	100.8	62.06	34.85	20.24	11.65	6.56	3.66	2.05
Plastic products	114.04	282.09	290.05	183.93	109.62	65.89	37.51	21.07	11.77
Clay building materials	2.71	0.54	0.19	0.1	0.05	0.03	0.02	0.01	0.01
Glass and other ceramics n.e.c.	48.06	20.77	10.33	5.52	2.99	1.7	0.98	0.56	0.32
Cement, lime and plaster products	16.22	22.34	11.56	5.6	2.91	1.6	0.91	0.52	0.3
Other non-metallic mineral products n.e.c.	294.44	266.49	155.34	82.51	47.3	27.28	15.6	8.9	5.1
Basic iron and steel	488.78	666.67	1598.93	855.02	558.88	178.1	94.49	45.89	25.15
Basic aluminium	86.88	75.26	46.6	24.99	14.12	7.99	4.57	2.64	1.53
Basic Copper	33.39	19.45	9.46	5.21	2.84	1.56	0.87	0.49	0.28
Basic Gold	4.57	11.92	4.71	2.46	1.38	0.78	0.45	0.26	0.15
Basic lead/zinc/silver	10.78	21.67	7.25	3.98	2.28	1.3	0.75	0.43	0.25
Basic nickel	35.18	42.27	20.74	10.31	5.8	3.18	1.77	0.99	0.57
Basic tin	6.7	15.3	12.73	8.22	4.75	2.67	1.5	0.85	0.49
Basic non-ferrous metals n.e.c.	45.44	76.73	83.76	40.5	21.54	11.74	6.51	3.66	2.08
Fabricated metal products	38.03	105.34	128.98	109.5	66.09	39.91	22.97	13.35	7.73
Machinery and equipment	8.76	12.64	11.47	9.52	5.78	3.44	1.99	1.15	0.67
Motor vehicles, trailers and semi-trailers	347.19	1762.71	2305.72	2206.23	1662.55	1062.59	629.84	362.38	206.33
Other transport equipment	1.06	7.31	8.95	7.84	5.85	3.96	2.53	1.56	0.95
Repair and installation of machinery and equipment (service)	24.14	63.37	64.74	45.66	28.46	16.9	9.86	5.73	3.33
Computers; electronic products; optical and precision instruments	1.92	53.12	65.22	49.29	32.06	19.29	11.41	6.62	3.84
Electrical equipment	20.15	116.01	152.75	147.6	118.56	71.96	40.99	22.74	12.61
Furniture and other manufacturing n.e.c	0.98	7.75	6.93	5.05	3.25	1.95	1.15	0.66	0.38
Electric power generation, transmission and distribution	1021.87	190.17	63.95	23.36	11.9	6.87	4	2.29	1.28
Distribution of gaseous fuels through mains	7.43	11.87	13.1	4.92	2.85	1.66	0.97	0.57	0.32
Water collection, treatment and supply; sewerage	6.67	3.57	1.26	0.71	0.37	0.23	0.14	0.08	0.05
Waste collection, treatment, and disposal	2.24	0.72	0.19	0.1	0.05	0.03	0.02	0.01	0.01
Materials recovery	18.73	0.23	0.1	0.06	0.03	0.02	0.01	0.01	0
Building construction	0.41	8.75	8.32	5.28	3.78	2.31	1.35	0.77	0.45
Civil engineering construction	2.69	0.9	0.6	0.47	0.3	0.19	0.12	0.07	0.04
Wholesale and retail trade; repair of motor vehicles and motorcycles	14.57	107.28	61.45	41.12	26.9	16.14	9.73	5.73	3.36
Road transport	449.73	46.98	25.99	21.31	11.19	6.86	4.14	2.46	1.42
Rail transport	14.6	9.58	4.16	2.3	1.3	0.81	0.48	0.28	0.16
Transport via pipeline	3.33	0.69	0.46	0.34	0.19	0.11	0.06	0.04	0.02
Water transport	74.25	11.74	9.66	6.67	4.46	2.57	1.55	0.89	0.51
Air transport	2.75	1.56	1.19	0.88	0.5	0.3	0.17	0.1	0.06
Services to transport	19.08	30.59	10.9	5.93	3.33	2.01	1.19	0.69	0.4
Postal and courier services	0.08	0.47	0.15	0.09	0.06	0.04	0.02	0.01	0.01
Hospitality	0	0.05	0.06	0.03	0.02	0.01	0.01	0	0
Publishing	0.01	1.17	1.38	0.92	0.59	0.36	0.22	0.13	0.08
Telecommunications	0.01	0.54	0.59	0.43	0.29	0.19	0.12	0.07	0.04
Information services	0.09	5.06	3.85	2.59	1.73	1.11	0.7	0.42	0.25
Finance and insurance	0.51	10.48	8.03	5.66	3.73	2.37	1.47	0.89	0.53
Property and real estate	0.66	4.96	2.6	1.68	1.03	0.64	0.39	0.24	0.14
Professional, scientific and technical services	5.76	39.8	43.04	37.2	27.37	18.12	11.1	6.54	3.79
Administrative services	0.02	3.8	3.05	1.99	1.18	0.7	0.41	0.24	0.14
Government; social security; defence; public order	0	0.11	0.08	0.05	0.03	0.02	0.01	0.01	0
Education	0.51	0.39	0.17	0.13	0.07	0.05	0.03	0.02	0.01
Human health and social work activities	0	0.04	0.03	0.02	0.01	0.01	0	0	0
Arts, entertainment and recreation	0	0.08	0.06	0.04	0.02	0.01	0.01	0	0
Other services	1.21	2.18	2.13	1.37	0.8	0.48	0.28	0.16	0.09

SI-7.4 GLORIA and nested MRIO comparison

This model expansion results in the disaggregation of the single GLORIA sector *Motor vehicles, trailers and semi trailers* into *Passenger motor cars (hybrid cars)*, *Passenger motor cars (except hybrid cars)*, *Trucks, buses and miscellaneous cars*, *Two-wheel motor vehicles*, *Internal combustion engines for motor vehicles* and *Motor vehicle parts and accessories*.

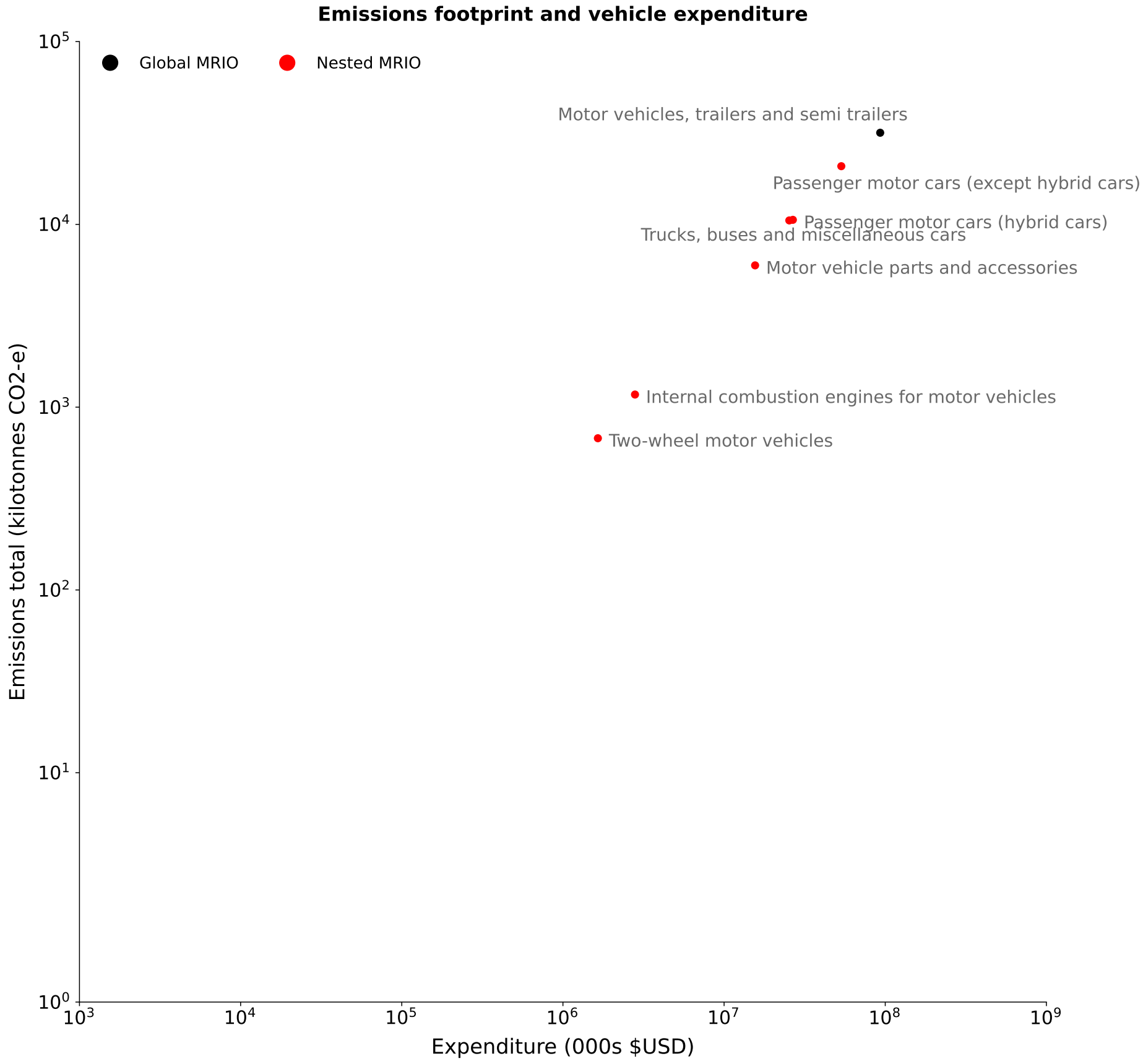

Figure 14: Effect of emissions footprints of nesting process

SI-8 Sensitivity analysis

SI-8.1 Battery capacity

To assess the effect of variation in battery capacity on production footprints, battery capacity data was compiled from Japanese vehicle manufacturers, as well as from China and the EU (Mai et al., 2025). Figure 15 shows the distribution of these data by powertrain and vehicle size, overlaid with a normal distribution. To the test the propagation of sensitivity from the input battery capacity to the final production footprint, we took a Monte Carlo approach. Random samples were taken from the battery capacity distributions over 100 simulations, with each simulation randomly perturbing the battery capacity. Footprints were recalculated for each simulation run. We assumed battery weight was linearly proportional to capacity and perturbed the battery component weight by the same factor. The sensitivity of the production footprint to changes in battery capacity are shown in Figure 16.

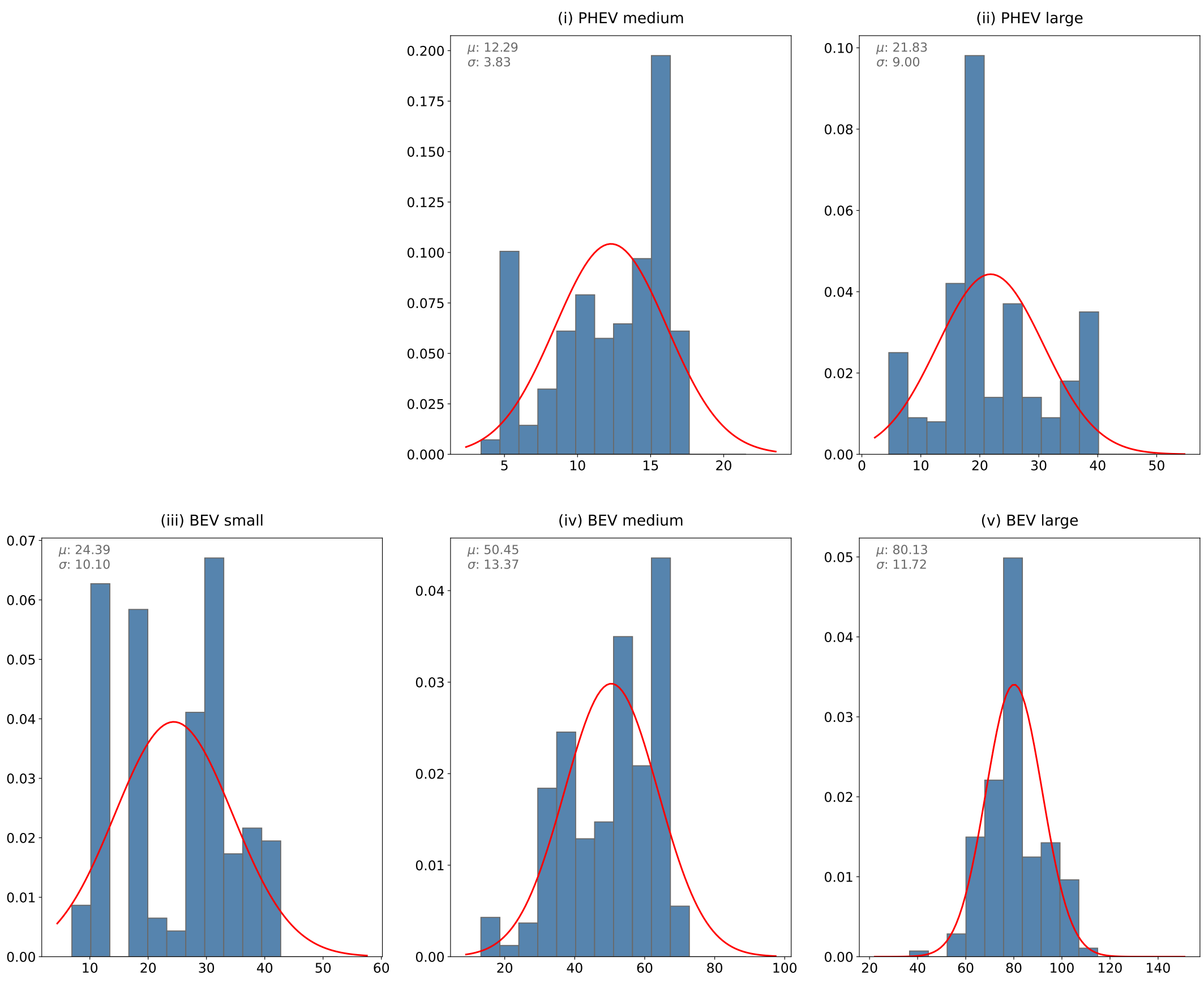

Figure 15: Battery capacity distribution by powertrain and size

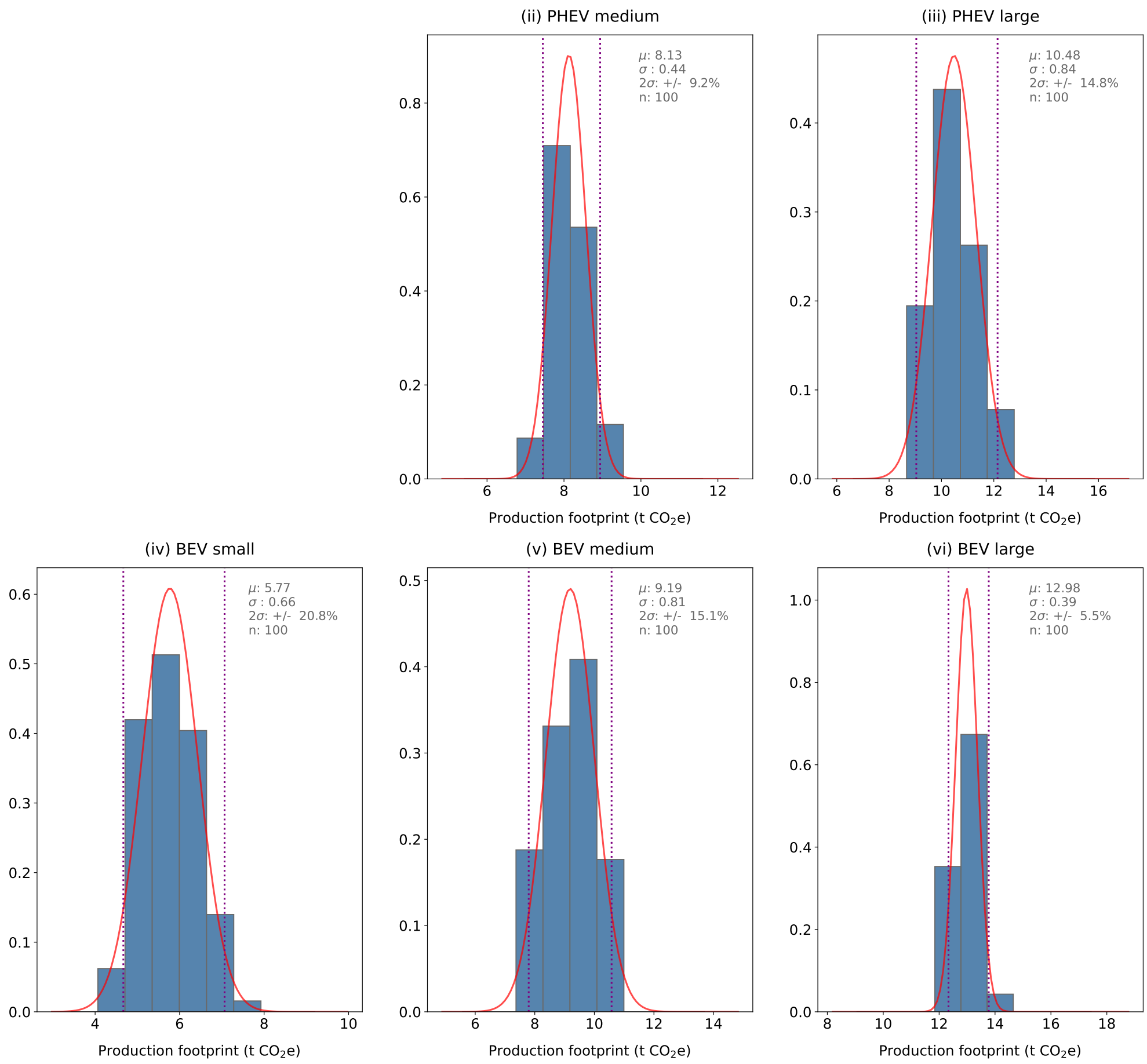

Figure 16: Sensitivity of production footprints to changes in battery capacity. The dashed purple lines indicate the 95% confidence interval around the mean.

SI-8.2 Comtrade prices

Prices derived from the Comtrade database were compiled to test the sensitivity of the final vehicle production footprints to variation in these prices. Commodity prices are used in model construction when linking physical components to the monetary blocks. The distribution of prices is shown in Figure 17, for the subset of commodities important to vehicle production, and approximated using a log-normal distribution. A Monte Carlo approach was taken to test the propagation of sensitivity from the input commodity price to the final production footprint. Random samples were taken from the distribution of prices over 100 simulations, with each simulation randomly perturbing the price used in the model. Footprints were recalculated for each simulation run. The results of this analysis are shown in Figure 18.

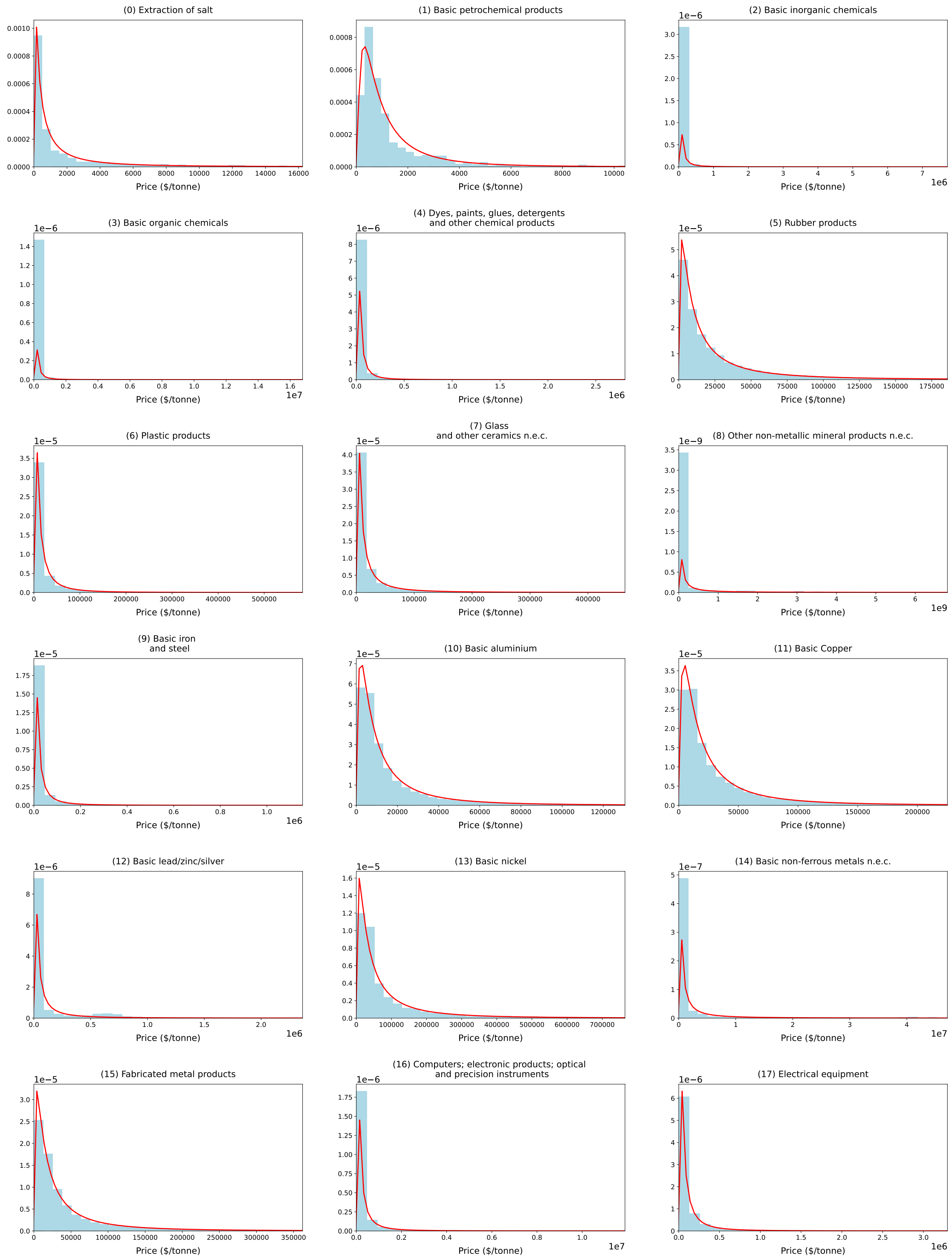

Figure 17: Distribution of material prices, derived from the UN Comtrade database. Log-normal distributions are overlaid in red.

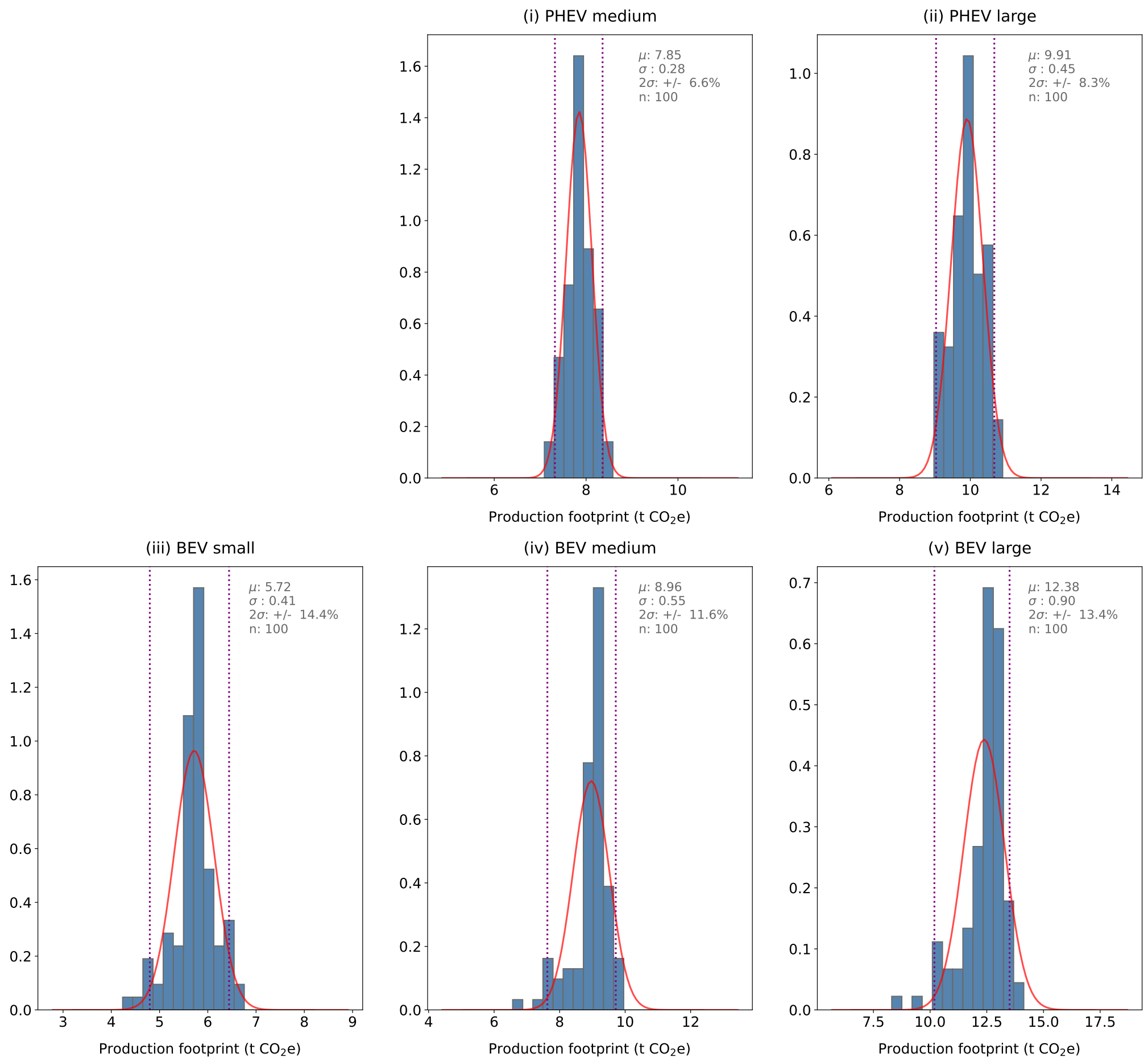

Figure 18: Sensitivity of production footprints to changes in commodity prices. The dashed purple lines indicate the 95% confidence interval around the mean.

References

DCCEEW (2024). Australian National Greenhouse Accounts Factors 2024. *Department of Climate Change, Energy, the Environment and Water (DCCEEW), Commonwealth of Australia.* https://www.dcceew.gov.au/sites/default/files/documents/national-greenhouse-account-factors-2024.pdf.

Finger, J. M. and Kreinin, M. E. (1979). A Measure of 'Export Similarity' and Its Possible Uses. *The Economic Journal*, 89(356):905–912.

Gaulier, G. and Zignago, S. (2022). BACI: International Trade Database at the Product-Level. The 1994-2007 Version. *CEPII.* http://www.cepii.fr/CEPII/fr/publications/wp/abstract.asp?NoDoc=2726.

IEA (2024). Global EV Outlook 2024. *International Energy Agency.* https://www.iea.org/reports/global-ev-outlook-2024.

IEEJ (2024). IEEJ Outlook 2024. *The Institute of Energy Economics, Japan.* https://eneken.ieej.or.jp/data/11456.pdf.

JAMA (2024). The Motor Industry of Japan 2024. *Japan Automobile Manufacturers Association (JAMA).* https://www.jama.org/the-motor-industry-of-japan-2024/.

Mai, L., Liu, M., Hao, H., Sun, X., Meng, F., Geng, Y., Wadud, Z., Tate, J. E., Dong, Z., Li, H., Geng, J., Dou, H., Deng, Y., Bai, F., Liu, Z., and Zhao, F. (2025). A high-resolution dataset on electric passenger vehicle characteristics in China and the European Union. *Scientific Data*, 12(1):1449.

METI (2020). Statistics on supply and demand of non-ferrous metals, etc. *Ministry of Economy, Trade and Industry, Agency for Natural Resources and Energy, Resources and Fuel Department, Mineral Resources Division.* https://www.enecho.meti.go.jp/statistics/coal_and_minerals/cm002/pdf/2019/r01nenpou.pdf.

Nakamura, S. and Nakajima, K. (2005). Waste Input–Output Material Flow Analysis of Metals in the Japanese Economy. *Materials Transactions*, 46(12):2550–2553.

NREL (2021). Life Cycle Greenhouse Gas Emissions from Electricity Generation: Update. *National Renewable Energy Laboratory (NREL).* https://docs.nrel.gov/docs/fy21osti/80580.pdf.

Ohno, H., Matsubae, K., Nakajima, K., Nansai, K., Fukushima, Y., and Nagasaka, T. (2016). Consumption-based accounting of steel alloying elements and greenhouse gas emissions associated with the metal use: the case of Japan. *Journal of Economic Structures*, 5(1):28.

Park, K., Yu, J., Coyle, J., Dai, Q., Frisco, S., Zhou, M., and Burrell, A. (2021). Direct Cathode Recycling of End-Of-Life Li-Ion Batteries Enabled by Redox Mediation. *ACS Sustainable Chemistry & Engineering*, 9(24):8214–8221.

Wang, M., Elgowainy, A., Lee, U., Baek, K. H., Balchandani, S., Benavides, P. T., Burnham, A., Cai, H., Chen, P., Gan, Y., Gracida-Alvarez, U. R., Hawkins, T. R., Huang, T.-Y., Iyer, R. K., Kar, S., Kelly, J. C., Kim, T., Kolodziej, C. P., Lee, K., Liu, X., Lu, Z., Masum, F. H., Morales, M., Ng, C., Ou, L., Poddar, T. K., Reddi, K., Shukla, S., Singh, U., Sun, L., Sun, P., Vyawahare, P., and Zhang, J. (2023). Summary of Expansions and Updates in R&D GREET 2023. Technical Report ANL/ESIA-23/10, Argonne National Laboratory (ANL), Argonne, IL (United States).

Waugh, F. V. (1950). Inversion of the Leontief matrix by power series. *Econometrica*, 18(2):142–154.

Weiss, M., Winbush, T., Newman, A., and Helmers, E. (2024). Energy Consumption of Electric Vehicles in Europe. *Sustainability*, 16(17):7529.

WTO (2024). Trade data reveal changing patterns in electric vehicles market. *World Trade Organization.* https://www.wto.org/english/blogs_e/data_blog_e/blog_dta_14may24_e.htm.
